# Supplementary material for: Thioglycoligase derived from fungal GH3 β-xylosidase is a multi-glycoligase with broad acceptor tolerance
Source: Nat Commun. 2020 Sep 25;11:4864. doi: 10.1038/s41467-020-18667-3 (PMC7519651; doi:10.1038/s41467-020-18667-3)
Supplement: Supplementary file 1 — Supplementary Information [file 41467_2020_18667_MOESM1_ESM.pdf]

SUPPLEMENTARY INFORMATION

Thioglycoligase derived from fungal GH3 β-xylosidase is a multi-glycoligase with broad acceptor tolerance

Manuel Nieto-Domínguez[1]*, Beatriz Fernández de Toro[2], Laura I. de Eugenio[1], Andrés G. Santana[3], Lara Bejarano-Muñoz[1], Zach Armstrong[4], Juan Antonio Méndez-Líter[1], Juan Luis Asensio[3], Alicia Prieto[1], Stephen G. Withers[4], Francisco Javier Cañada[2] and María Jesús Martínez[1]*

[1]Biotechnology for Lignocellulosic Biomass Group, Centro de Investigaciones Biológicas Margarita Salas (CSIC), C/Ramiro de Maeztu 9, 28040, Madrid, Spain.

[2]NMR and Molecular Recognition Group, Centro de Investigaciones Biológicas Margarita Salas (CSIC), C/Ramiro de Maeztu 9, 28040, Madrid, Spain.

[3]Glycochemistry and Molecular recognition group. Instituto de Química Orgánica General (CSIC), C/Juan de la Cierva, 3, 28006 Madrid, Spain.

[4]Department of Chemistry, Centre for High-Throughput Biology, University of British Columbia, Canada.

*Corresponding authors: mjmartinez@cib.csic.es (M.J. Martínez); manunieto@cib.csic.es (M. Nieto-Domínguez)

TABLE OF CONTENTS

SUPPLEMENTARY TABLES 3
SUPPLEMENTARY FIGURES 8
SUPPLEMENTARY DISCUSSION 125
SUPPLEMENTARY METHODS 129
SUPPLEMENTARY REFERENCES 137

SUPPLEMENTARY TABLES

Supplementary Table 1. Summary of pH profile, family, and source of the thioglycoligases reported up to date.

Enzyme	Organism	Family	pH[a]	Reference
β-Xylosidase (BxTW1)	*Talaromyces amestolkiae*	GH3	Optimum: 3<br>pH ≤ 3: Active	Wild-type[1]<br>Thioglycoligase: This work
β-Glucosidase (Abg)	*Agrobacterium* sp.	GH1	Optimum: 6-7<br>pH ≤ 3: Inactive	Wild-type[2]<br>Thioglycoligase[3]
β-Mannosidase (Man2A)	*Cellulomonas fimi*	GH2	Optimum: 7<br>pH ≤ 3: Unstable < 5	Wild-type[4,5]<br>Thioglycoligase[3]
β-Glucuronidase (TMGUA)	*Thermotoga maritima*	GH2	Optimum: 6<br>pH ≤ 3: Inactive	Wild-type[6]<br>Thioglycoligase[7]
Endoxylanase (Bcx)	*Bacillus circulans*	GH11	Optimum: 6<br>pH ≤ 3: Residual	Wild-type[8]<br>Thioglycoligase[9]
Cyclodextrin glucanotransferase (Cgt)	*Bacillus* sp. I-5	GH13	Optimum: 6<br>pH ≤ 3: No data	Wild-type[10,11]<br>Thioglycoligase[12]
β-Hexosaminidase (SpHex)	*Streptomyces plicatus*	GH20	Optimum: 3<br>pH ≤ 2.5: Residual	Wild-type[13]<br>Thioglycoligase[14]
α-Glucosidase (MalA)	*Sulfolobus solfataricus*	GH31	Optimum: 4.5<br>pH ≤ 3: Residual	Wild-type[15]<br>Thioglycoligase[16]
α-Xylosidase (YicI)	*Escherichia coli*	GH31	Optimum: 7<br>pH ≤ 3: Inactive	Wild-type[17]<br>Thioglycoligase[16]
β-Galactosidase (BgaX)	*Xanthomonas manihotis*	GH35	Optimum: 4.5<br>pH ≤ 3: No data	Wild-type[18]<br>Thioglycoligase[19]
α-L-Arabinofuranosidase (Araf51)	*Ruminiclostridium thermocellum*	GH51	Optimum: 7-8<br>pH ≤ 3: No data	Wild-type[20]<br>Thioglycoligase[21]
α-*N*-Acetyl-glucosaminidase (CpGH89)	*Clostridium perfringens*	GH89	Optimum: 7.3<br>pH ≤ 3: Inactive	Wild-type[22]<br>Thioglycoligase[23]

[a] pH data are referred to the wild-type glycosidases.

Supplementary Table 2. Kinetic parameters of acid/base mutants from rBxTW1 against *p*NPX.

Buffer	Mutant	K_m (mM)	V_{max} ($\mu mol \cdot min^{-1} \cdot mg^{-1}$)	k_{cat} (s^{-1})	k_{cat}/K_m (mM^{-1} s^{-1})
Sodium acetate	E495A	0.67±0.03	0.72±0.01	2.41±0.03	3.6±0.1
	E495G	0.23±0.01	0.288±0.003	0.96±0.01	4.2±0.1
	E495Q	0.20±0.01	0.266±0.003	0.89±0.01	4.5±0.3
Sodium citrate	E495A	0.16±0.01	0.094±0.002	0.314±0.005	2.0±0.1
	E495G	0.10±0.01	0.060±0.001	0.201±0.002	2.0±0.1
	E495Q	0.16±0.01	0.084±0.001	0.280±0.004	1.8±0.1

[a] Rate of catalysis was determined in triplicate for each *p*NPX concentration. Values of the kinetic parameters are shown together with the corresponding standard error (n = 3 independent experiments). Source data are provided as a Source Data file.

Supplementary Table 3. Maximal production of xylosides from hydroxycinnamic acids: optimal conditions, xyloside concentrations (predicted and experimental) and conversion yields.

p-Coumaroyl xyloside	[*p*NPX] (mM)	T (ºC)	[Enzyme] (g/L)	[NaOH] (mM)	Time (min)	[Xyloside]$_{pred}$ (mM)	[Xyloside]$_{exp}$ (mM)	Yield (%)[a] 35/36/37
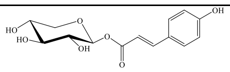 (35)	50	50	2.0	5.0	30	8.18	8.17	**32.7**/3.9/1.1
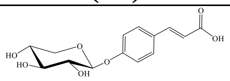 (36)	50	30	8.0	15	120	18.24	19.82	7.6/**79.3**/7.7
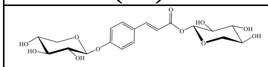 (37)	50	30	7.4	5.0	119	2.78	2.28	10.0/62.6/**9.1**
Feruloyl xyloside	[*p*NPX] (mM)	T (ºC)	[Enzyme] (g/L)	[NaOH] (mM)	Time (min)	[Xyloside]$_{pred}$ (mM)	[Xyloside]$_{exp}$ (mM)	Yield (%)[a] 39/40/41
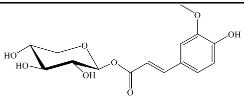 (39)	50	50	3.4	0.0	30	6.90	5.47	**21.9**/21.7/8.2
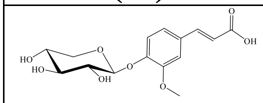 (40)	50	30	6.0	15	60	21.53	21.28	7.0/**82.7**/11.1
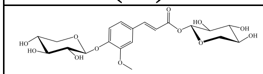 (41)	50	30	6.0	0.3	60	4.88	4.32	15.8/50.5/**17.3**

[a] Bold value indicates the xyloside whose production was optimized.

Supplementary Table 4. Analysis of the glycosylation yields: Reaction conditions.

				Conditions		
				No controlled pH	Controlled pH	
Acceptor	Co-solvent	[Acceptor]	[*p*NPX]	Initial	pH	Buffer
trans-Cinnamic acid	Ethanol	20 mM	40 mM	~3	3.0	Glycine-HCl
Vanillin	-			~5	5.5	MES
EGCG	-			~5	5.5	MES
N-Hydroxybenzotriazole	Ethanol			~4	3.5	Glycine-HCl
3,5-Dibromo-1,2,4-triazole	Ethanol			~5	5.0	MES
Thiophenol	Methanol			~5	5.5	MES
Benzenesulfínic acid	-			~2	2.2	Glycine-HCl
Benzeneselenol	Methanol			~3	3.0	Glycine-HCl
Phosphoric acid	-	80 mM		~2	2.2	Glycine-HCl

Supplementary Table 5. CCD experimental design for production of xylosides from *p*-coumaric acid.

[*p*NPX] (mM)	T (ºC)	[Enzyme] (g/L)	[NaOH] (mM)	Time (min)	[*p*Coumaroyl xyloside 35] (mM)	[*p*Coumaroyl xyloside 36] (mM)	[*p*Coumaroyl xyloside 37] (mM)
30	16	5	10	75	3.25	7.30	1.09
10	30	2	5	30	1.61	3.31	0.26
10	30	2	15	30	1.49	4.31	0.20
50	30	2	5	30	8.08	2.14	0.93
50	30	2	15	30	7.24	3.29	0.91
10	30	8	5	30	0.41	4.97	0.11
10	30	8	15	30	0.32	6.28	0.06
50	30	8	5	30	5.57	7.48	2.59
50	30	8	15	30	4.33	10.59	2.12
10	30	2	5	120	0.57	5.33	0.15
10	30	2	15	120	0.52	6.70	0.11
50	30	2	5	120	6.77	7.28	2.77
50	30	2	15	120	5.37	6.89	1.59
10	30	8	5	120	0.09	2.92	0.00
10	30	8	15	120	0.08	4.04	0.00
50	30	8	5	120	2.56	15.84	2.60
50	30	8	15	120	1.99	18.90	2.10
30	40	5	0	75	1.94	10.44	0.99
30	40	5	22	75	1.84	11.91	0.77
0	40	5	10	75	0.00	0.00	0.00
70	40	5	10	75	4.52	13.52	3.54
30	40	0	10	75	0.00	0.00	0.00
30	40	12	10	75	0.63	12.34	0.33
30	40	5	10	15	4.24	6.47	1.12
30	40	5	10	180	0.75	13.53	0.40
0	40	5	10	75	1.60	13.23	0.95
30	40	5	10	75	1.69	14.06	0.96
30	40	5	10	75	1.45	13.60	0.90
30	40	5	10	75	1.60	13.62	0.92
30	40	5	10	75	1.58	13.71	0.95
30	40	5	10	75	1.63	13.55	0.98
30	40	5	10	75	1.51	13.60	0.93
30	40	5	10	75	1.51	13.41	0.91
10	50	2	5	30	2.75	1.59	0.14
10	50	2	15	30	1.49	3.86	0.18
50	50	2	5	30	7.78	1.28	0.49
50	50	2	5	30	6.25	1.02	0.27
10	50	8	5	30	0.80	3.39	0.09
10	50	8	15	30	0.32	4.70	0.04
50	50	8	5	30	7.45	3.64	1.56
50	50	8	15	30	4.92	5.10	1.18
10	50	2	5	120	2.61	2.21	0.19

10	50	2	15	120	1.31	4.65	0.16
50	50	2	5	120	9.19	2.16	1.02
50	50	2	15	120	7.50	2.39	0.73
10	50	8	5	120	0.39	1.28	0.01
10	50	8	15	120	0.14	2.04	0.00
50	50	8	5	120	7.29	3.77	1.61
50	50	8	15	120	5.51	9.19	1.97
30	64	5	10	75	2.59	0.23	0.01

Supplementary Table 6. CCD experimental design for production of xylosides from ferulic acid.

[*p*NPX] (mM)	T (ºC)	[Enzyme] (g/L)	[NaOH] (mM)	Time (min)	[Feruloyl xyloside 39] (mM)	[Feruloyl xyloside 40] (mM)	[Feruloyl xyloside 41] (mM)
35	16	4	7.5	45	4.92	7.29	1.65
20	30	6	15	60	1.38	15.03	0.88
50	30	6	0	60	4.69	13.48	4.91
50	30	6	15	60	2.12	21.53	3.45
20	30	2	15	60	1.89	13.22	1.04
20	30	2	15	30	2.26	10.37	0.92
50	30	6	0	30	5.94	10.79	4.38
50	30	2	15	30	3.94	11.38	2.26
50	30	2	0	60	6.56	7.73	3.32
20	30	2	0	30	3.02	4.95	1.00
50	30	6	15	30	2.71	18.78	3.26
20	30	2	0	60	2.80	6.69	1.48
20	30	6	0	60	2.07	7.93	1.25
20	30	6	15	30	1.64	14.33	1.01
50	30	2	0	30	6.77	4.66	1.62
50	30	2	15	60	3.25	16.51	3.06
20	30	6	0	30	2.52	7.84	1.35
35	40	4	7.5	45	3.01	14.36	2.85
35	40	4	7.5	45	3.04	14.11	2.77
35	40	4	7.5	45	3.04	14.08	2.80
35	40	4	7.5	9	4.88	7.23	1.79
35	40	0	7.5	45	0.00	0.00	0.00
35	40	4	7.5	45	3.00	13.96	2.70
35	40	4	7.5	45	3.00	13.33	2.63
35	40	9	7.5	45	2.17	13.17	1.94
35	40	4	25	45	1.05	7.14	0.52
0	40	4	7.5	45	0.00	0.00	0.00
35	40	4	7.5	45	2.91	14.00	2.70
35	40	4	7.5	45	2.95	13.97	2.69
35	40	4	0	45	4.02	11.57	2.95
71	40	4	7.5	45	4.32	14.92	5.66
35	40	4	7.5	81	2.75	15.40	2.50

35	40	4	7.5	45	2.84	13.28	2.55
20	50	6	0	60	2.68	7.45	0.87
20	50	2	15	60	1.73	11.59	0.81
50	50	6	15	30	3.76	11.31	2.31
20	50	2	0	60	3.32	5.36	1.04
50	50	6	0	30	6.82	6.60	2.77
20	50	6	0	30	2.23	6.57	1.07
50	50	2	15	30	5.01	5.78	1.43
20	50	2	0	30	3.49	4.18	0.76
20	50	6	5	30	1.70	10.74	0.74
50	50	2	0	60	6.90	3.90	1.23
20	50	6	15	60	1.30	10.75	0.57
50	50	2	0	30	6.51	3.27	0.90
50	50	6	15	60	3.58	12.15	2.41
50	50	2	15	60	4.73	6.91	1.76
50	50	6	0	60	6.73	8.08	3.46
20	50	2	15	30	2.47	8.27	0.80
35	64	4	7.5	45	3.93	1.61	0.21

Supplementary Table 7. Summary of ANOVA reports from the multiparametric models for the synthesis of each xyloside.

Source	Sum of Squares	df	Mean Square	F-value	p-value
Model (35)	295.49	20	14.77	8.85	< 0.0001
Model (36)	1043.99	20	52.20	7.24	< 0.0001
Model (37)	33.73	20	1.69	28.35	< 0.0001
Model (39)	127.28	20	6.36	9.96	< 0.0001
Model (40)	924.34	20	46.22	7.92	< 0.0001
Model (41)	72.41	20	3.62	31.74	< 0.0001

[a] *p*-values less than 0.05 indicate that developed equation model is significant.

SUPPLEMENTARY FIGURES

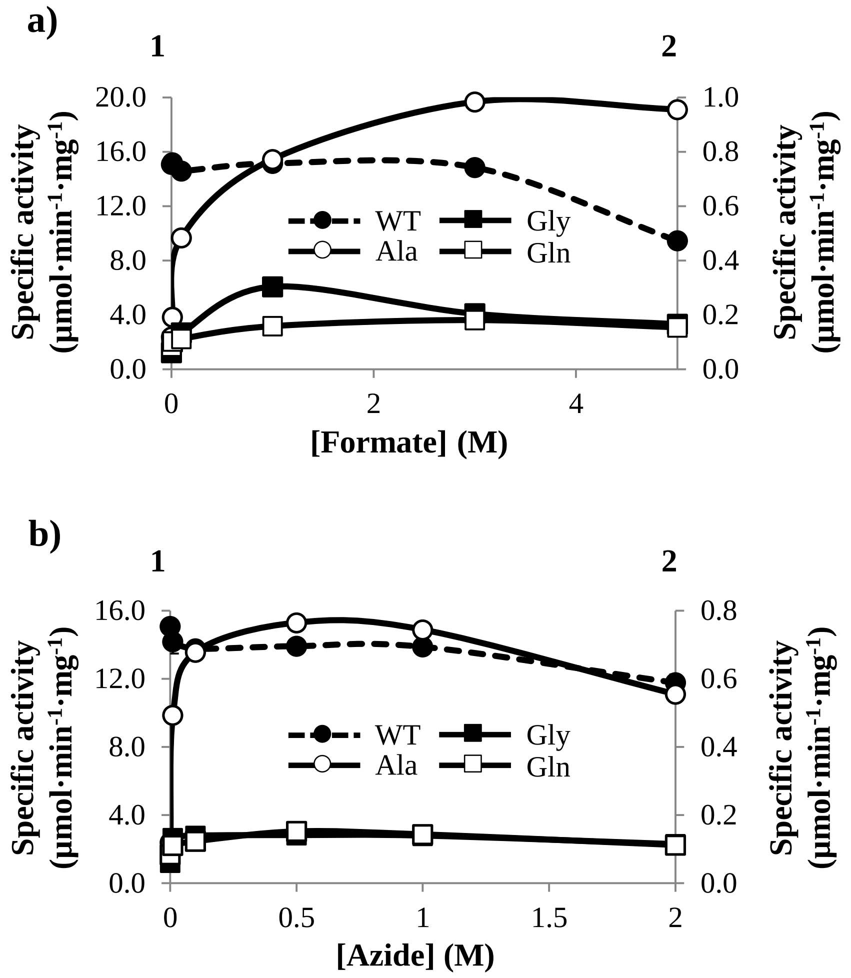

Supplementary Figure 1. Ligase activity response assays against small nucleophiles. Evolution of specific activities of rBxTW1 and acid/base mutants in a range of sodium formate (a) and azide (b) concentrations. Activity was calculated spectrophotometrically against *p*NPX. Values for wild type enzyme (WT) are referred to y-axis-1 while y-axis-2 corresponds to the mutants. Activity profiles are represented by a dashed line and black circles for wild type enzyme, by a continuous line and white circles for rBxTW1-E495A (Ala), by a continuous line and black squares for rBxTW1-E495G (Gly) and by a continuous line and white squares for rBxTW1-E495Q (Gln). Mean values are shown together with the corresponding standard error (n = 2 independent experiments). Source data are provided as a Source Data file.

a)
b)

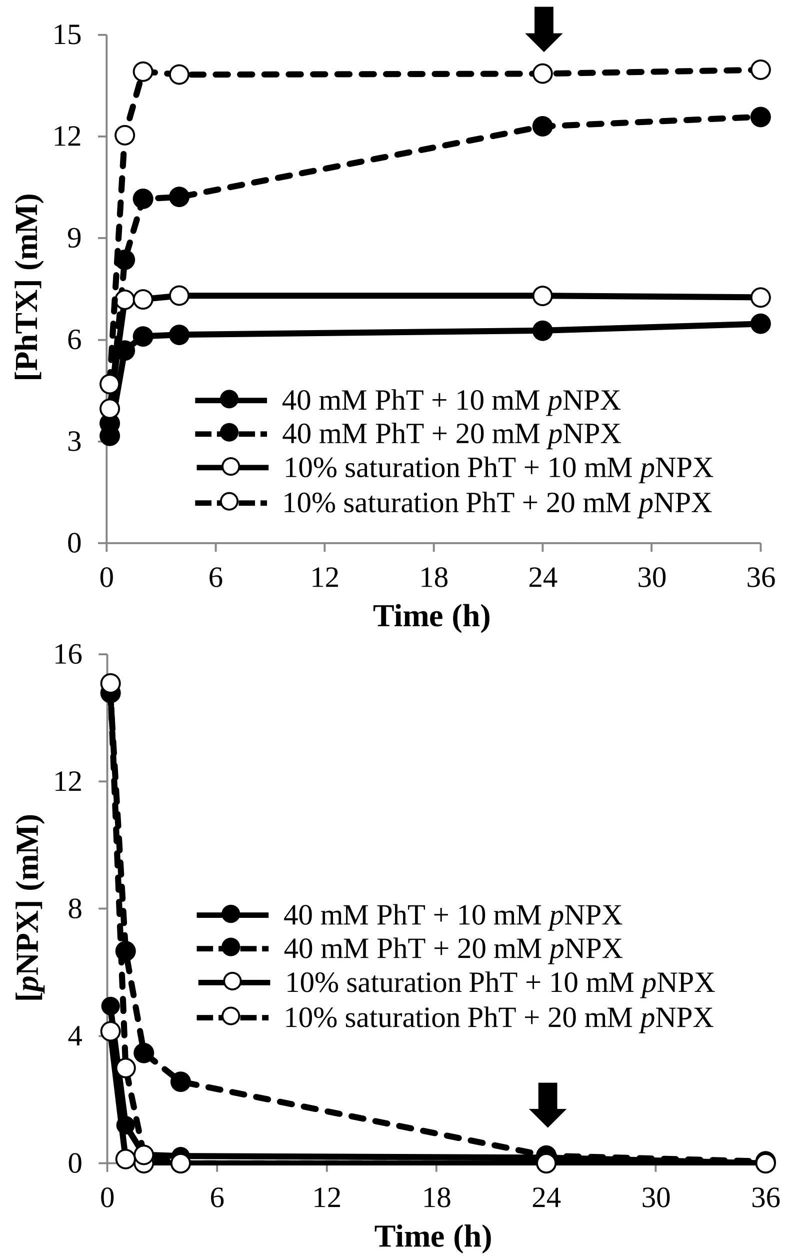

Supplementary Figure 2. Glycosylation of thiophenol. Formation of phenyl-β-D-thioxylopyranoside (a) and simultaneous consumption of 4-nitrophenyl β-D-xylopyranoside (b). Samples were analyzed by HPLC and concentrations of both PhTX and *p*NPX were determined by integrating peak areas and interpolating using the appropriate calibration curve. Black arrows indicate the addition of an aliquot of fresh enzyme. PhT: thiophenol; PhTX: phenyl-β-D-thioxylopyranoside. Determined profiles are represented by a continuous line and black circles for 40 mM PhT and 10 mM *p*NPX, by a dashed line and black circles for 40 mM PhT and 20 mM *p*NPX, by a continuous line and white circles for saturated PhT and 10 mM *p*NPX and by a dashed line and white circles for saturated PhT and 20 mM *p*NPX. Mean values are shown together with the corresponding standard error (n = 2 independent experiments). Source data are provided as a Source Data file.

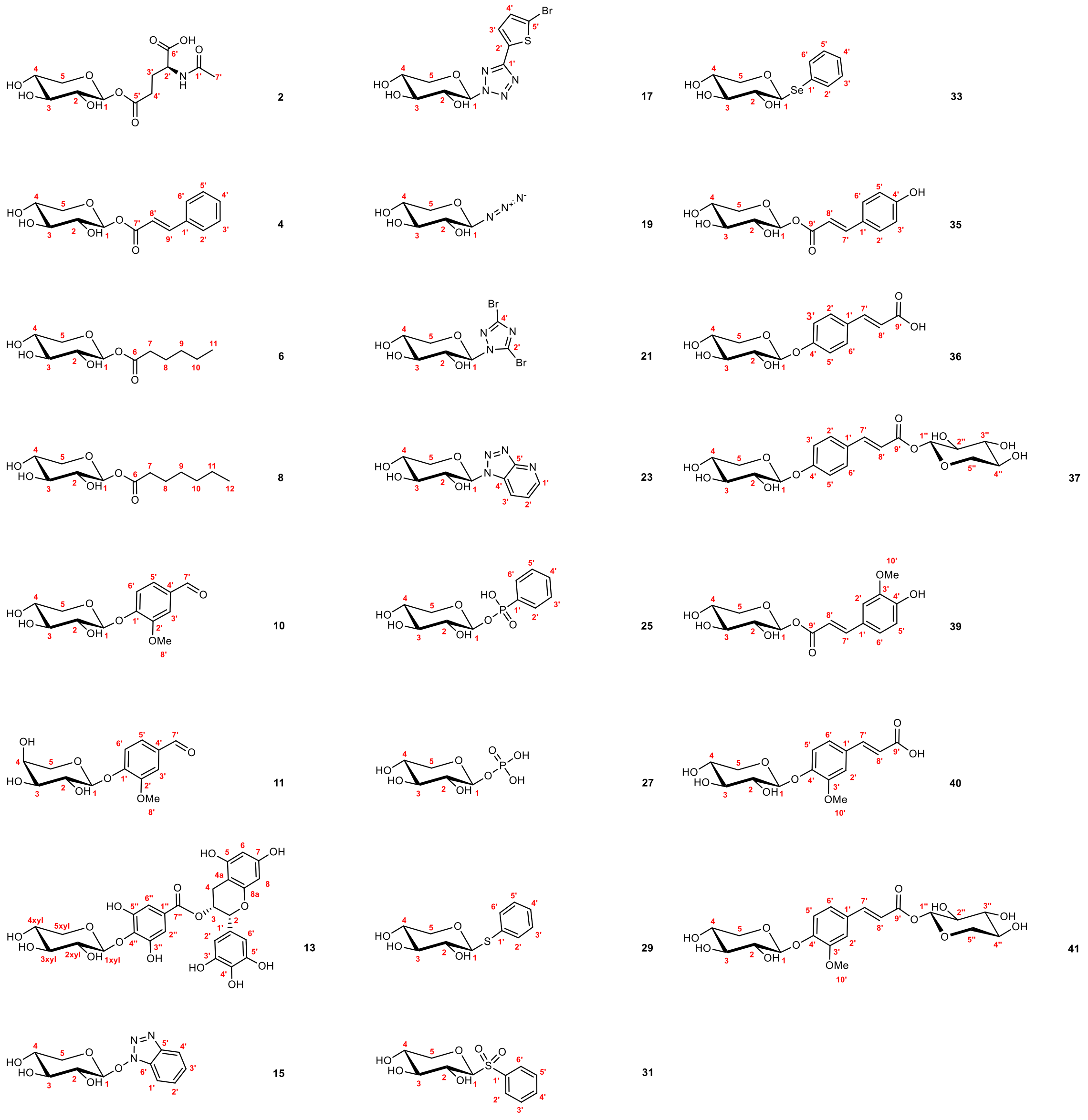

Supplementary Figure 3. Carbon numbering of identified products.

a)

(2)

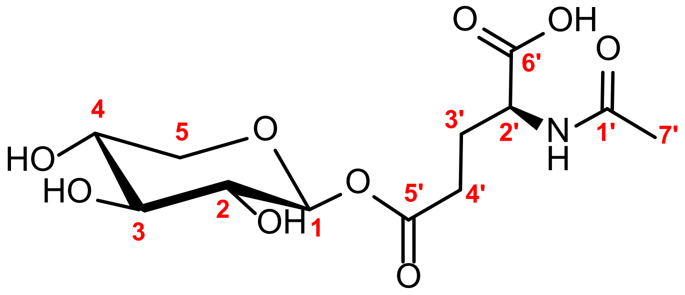
HO
HO
OH
O
1
2
3
4
5
O
5'
4'
3'
2'
N
H
1'
7'
6'
O
OH
O
O

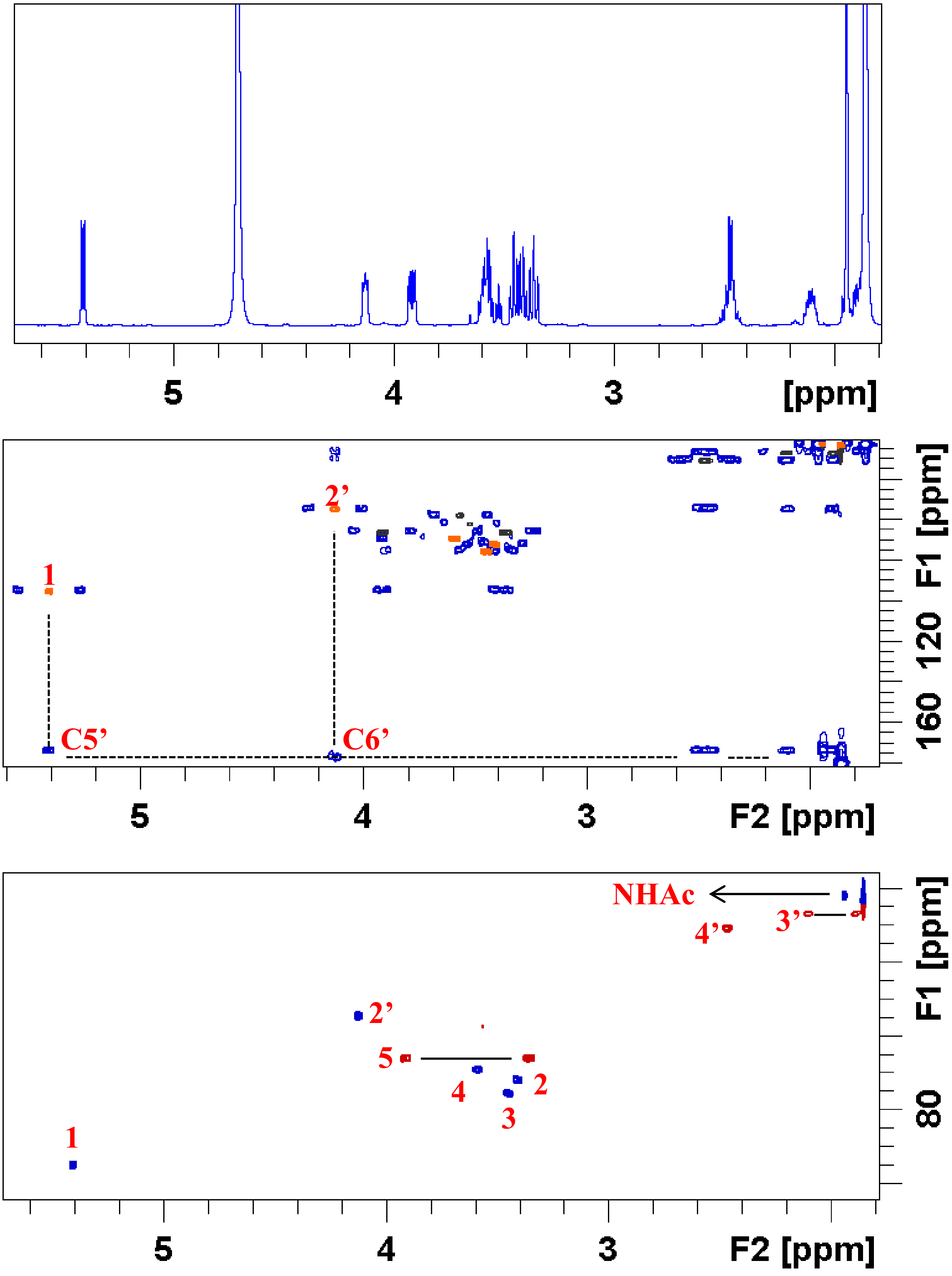
[ppm]
5
4
3
1
2'
C5'
C6'
120
160
F1 [ppm]
F2 [ppm]
NHAc
3'
4'
2'
5
4
3
2
1
80

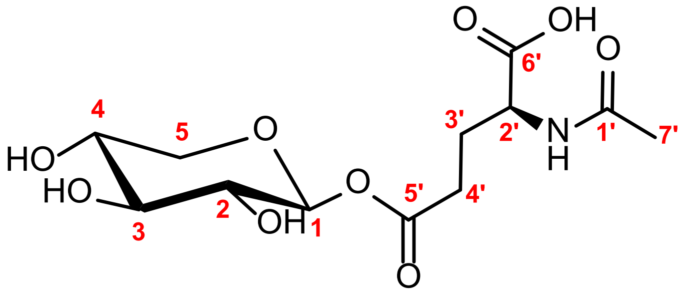

	^{1}H Chemical shift	^{13}C Chemical shift
**1**	5.41	94.74
**2**	3.41	71.70
**3**	3.44	75.41
**4**	3.59	68.83
**5**	3.36	65.77
**5**	3.90	
**1’**	------	173.92
**2’**	4.13	54.32
**3’**	1.89	26.53
**3’**	2.10	
**4’**	2.46	30.51
**5’**	------	173.92
**6’**	------	177.39
**7’**	1.94	21.61

b)

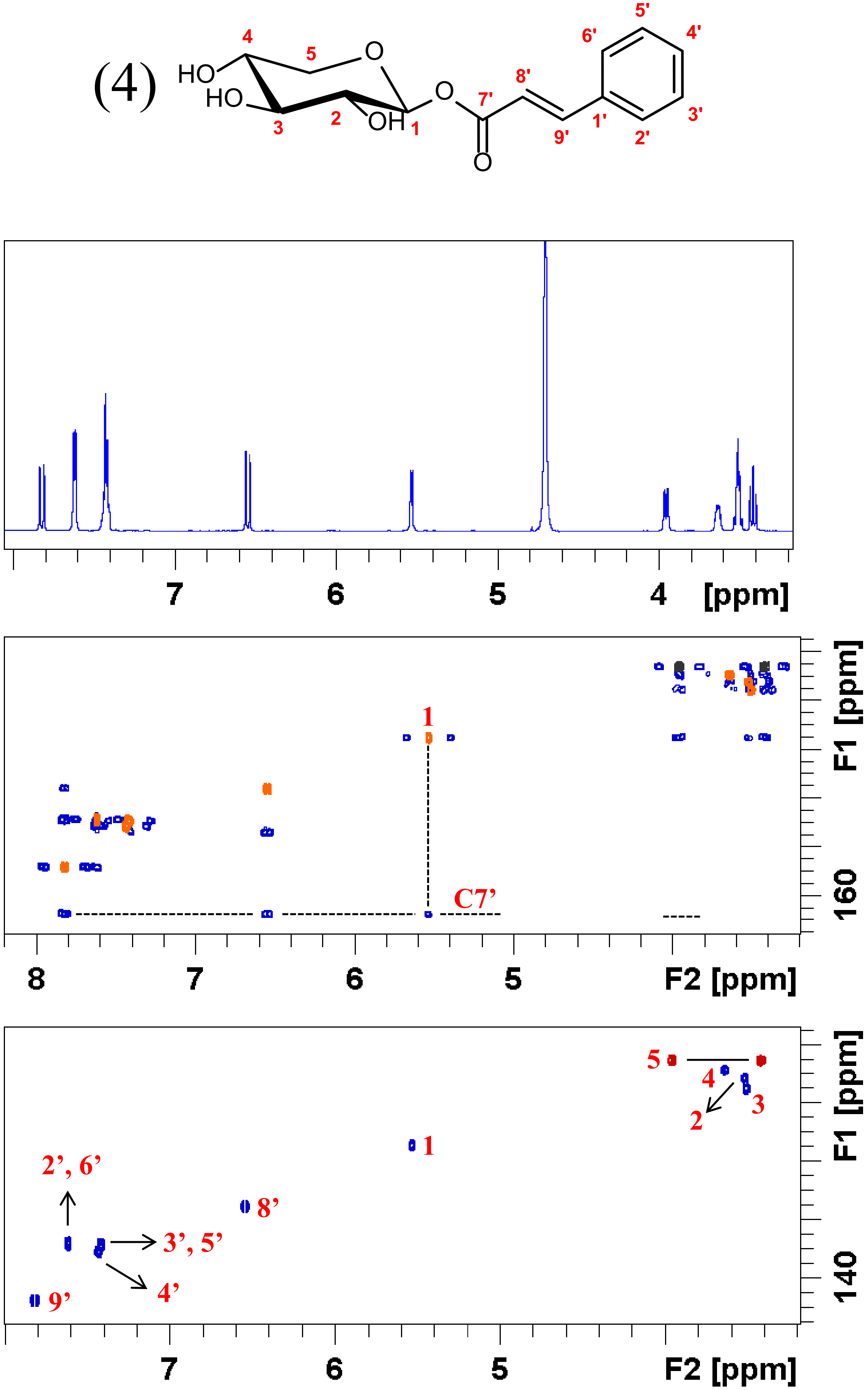
(4)
HO
HO
OH
O
O
O
1
2
3
4
5
1'
2'
3'
4'
5'
6'
7'
8'
9'
7
6
5
4
[ppm]
1
C7'
160
F1 [ppm]
8
7
6
5
F2 [ppm]
5
4
3
2
1
2', 6'
3', 5'
4'
8'
9'
140
F1 [ppm]
7
6
5
F2 [ppm]

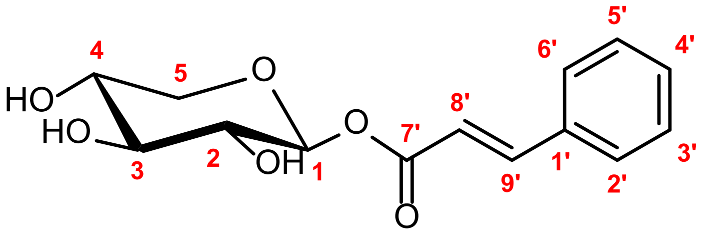

	^{1}H Chemical shift	^{13}C Chemical shift
**1**	5.53	94.85
**2**	3.51	71.26
**3**	3.51	75.42
**4**	3.63	69.00
**5**	3.41	65.79
**5**	3.95	
**1’**	------	133.81
**2’**	7.62	128.55
**3’**	7.42	129.08
**4’**	7.42	131.28
**5’**	7.42	129.08
**6’**	7.62	128.55
**7’**	------	167.14
**8’**	6.56	116.16
**9’**	7.81	148.03

c)

(6)

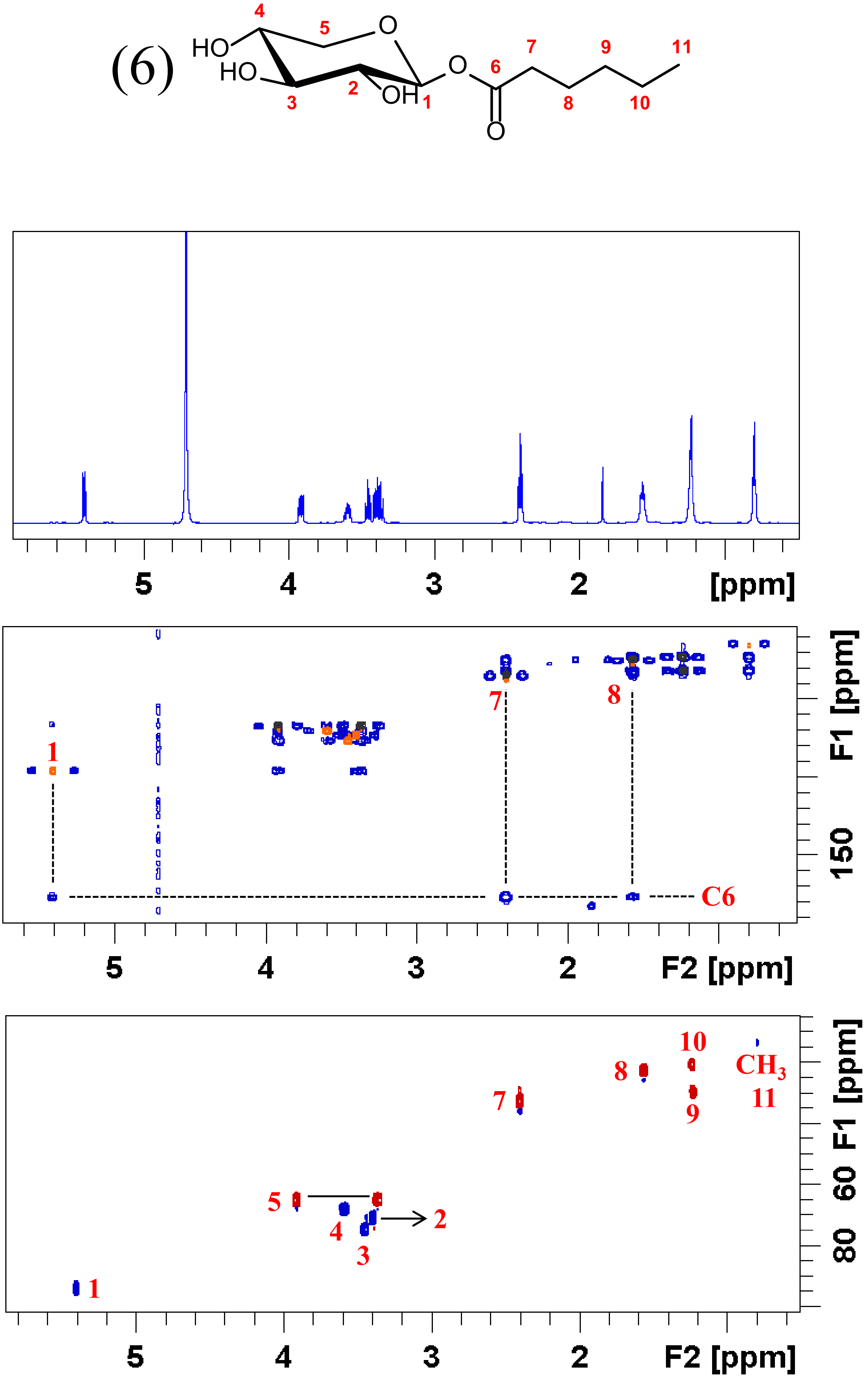

HO
HO
OH
O
O
O
1
2
3
4
5
6
7
8
9
10
11
5
4
3
2
[ppm]
F1 [ppm]
150
F2 [ppm]
C6
CH3
60
80

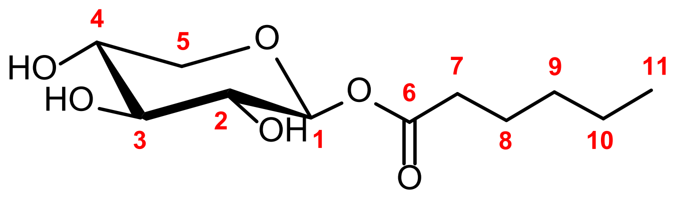

	^{1}H Chemical shift	^{13}C Chemical shift
**1**	5.40	94.43
**2**	3.40	71.49
**3**	3.44	75.26
**4**	3.59	68.66
**5**	3.35	65.67
**5**	3.90	
**6**	------	175.48
**7**	2.40	33.45
**8**	1.56	23.27
**9**	1.23	30.22
**10**	1.23	21.21
**11**	0.79	14.27

d)

(8)

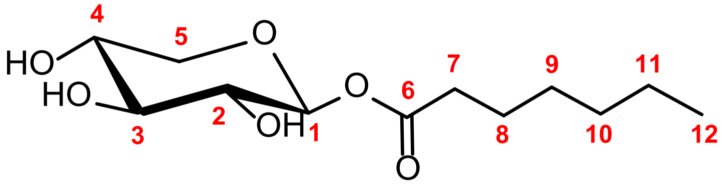

HO
HO
O
OH
O
O
1
2
3
4
5
6
7
8
9
10
11
12

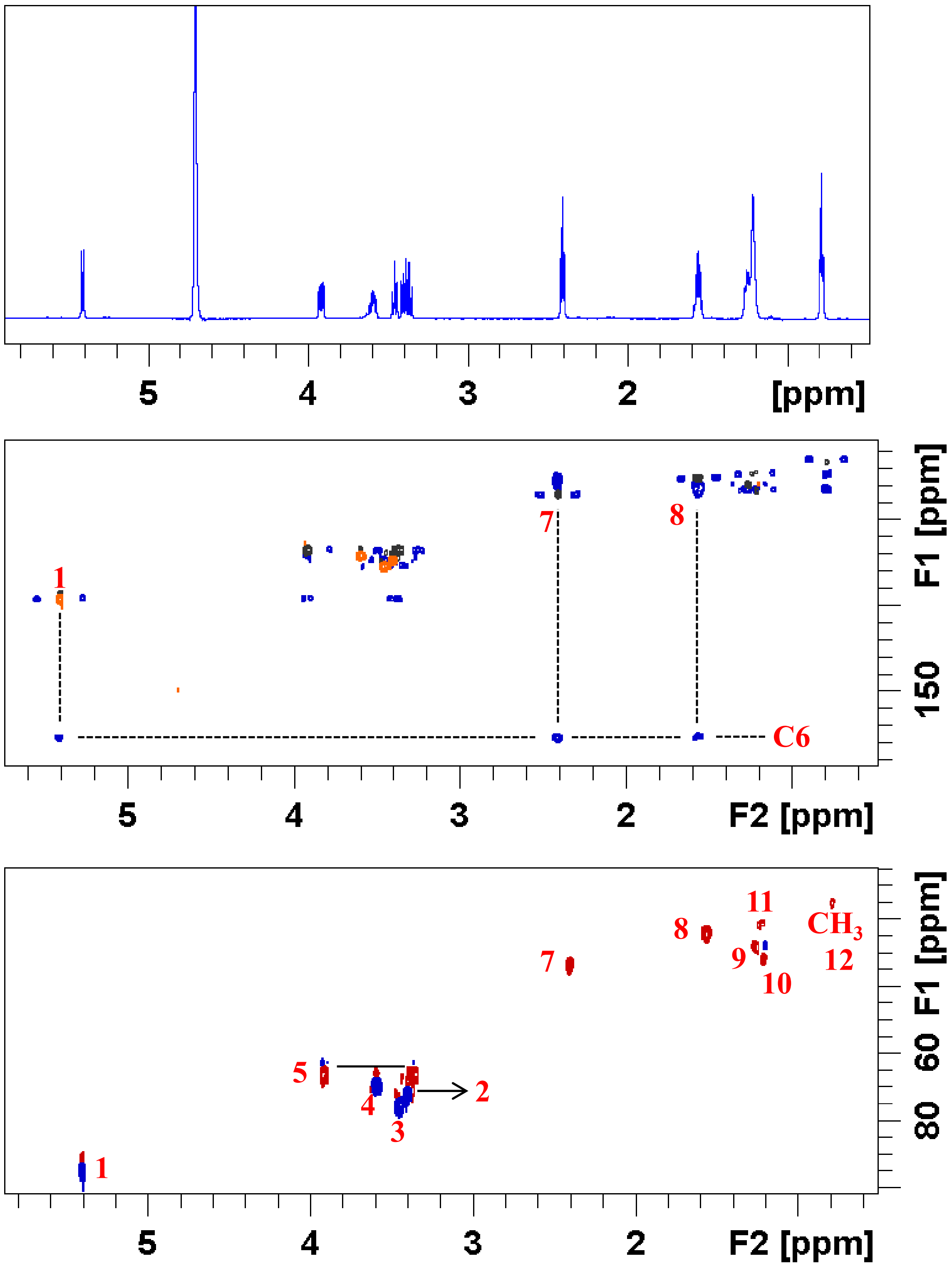

5
4
3
2
[ppm]
1
7
8
C6
150
F1 [ppm]
F2 [ppm]
11
CH$_3$
12
9
10
5
4
3
2
1
60
80
F1 [ppm]
F2 [ppm]

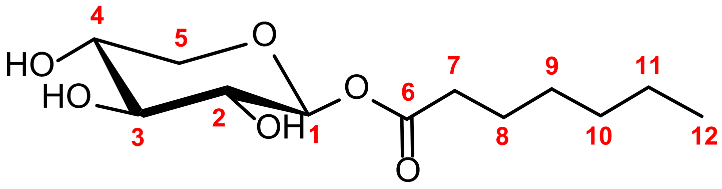

	^{1}H Chemical shift	^{13}C Chemical shift
**1**	5.40	94.87
**2**	3.40	72.16
**3**	3.47	75.70
**4**	3.59	69.26
**5**	3.38	65.86
**5**	3.92	
**6**	------	175.49
**7**	2.41	33.59
**8**	1.56	24.10
**9**	1.26	28.32
**10**	1.21	31.42
**11**	1.23	21.29
**12**	0.78	11.57

e)

(10)

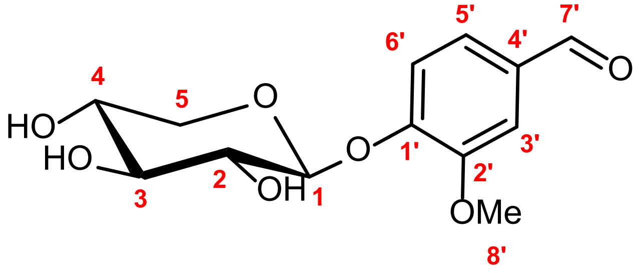
HO
HO
O
OH
O
OMe
O
1
2
3
4
5
1'
2'
3'
4'
5'
6'
7'
8'

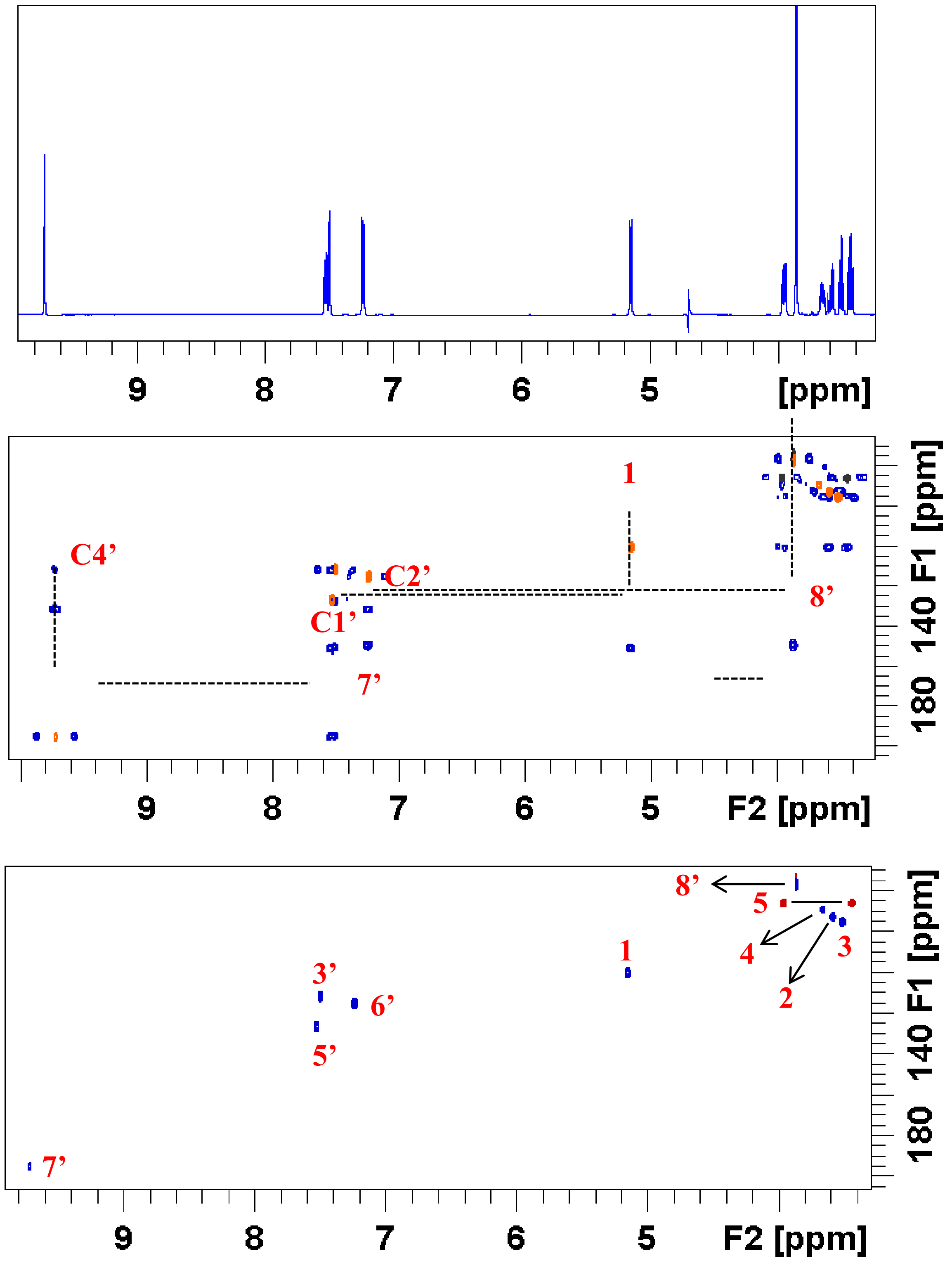
9
8
7
6
5
[ppm]
1
C4'
C2'
C1'
8'
7'
F1 [ppm]
140
180
F2 [ppm]
8'
5
4
3
2
1
3'
6'
5'
7'

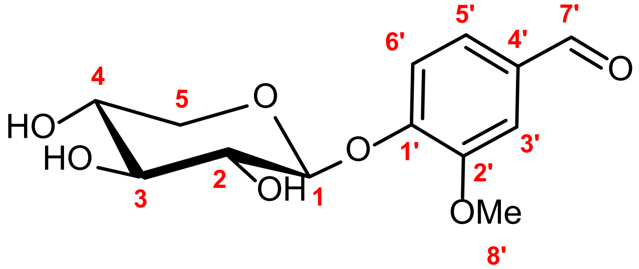

	^{1}H Chemical shift	^{13}C Chemical shift
**1**	5.15	100.21
**2**	3.59	72.67
**3**	3.51	75.21
**4**	3.66	69.17
**5**	3.44	65.74
**5**	3.95	
**1’**	---	150.70
**2’**	---	149.26
**3’**	7.50	111.80
**4’**	---	131.31
**5’**	7.52	126.60
**6’**	7.23	115.12
**7’**	9.72	194.37
**8’**	3.86	56.23

f)

(11)

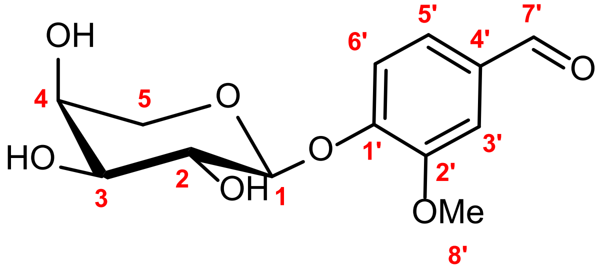
OH
HO
OH
O
O
O
OMe
1
2
3
4
5
1'
2'
3'
4'
5'
6'
7'
8'

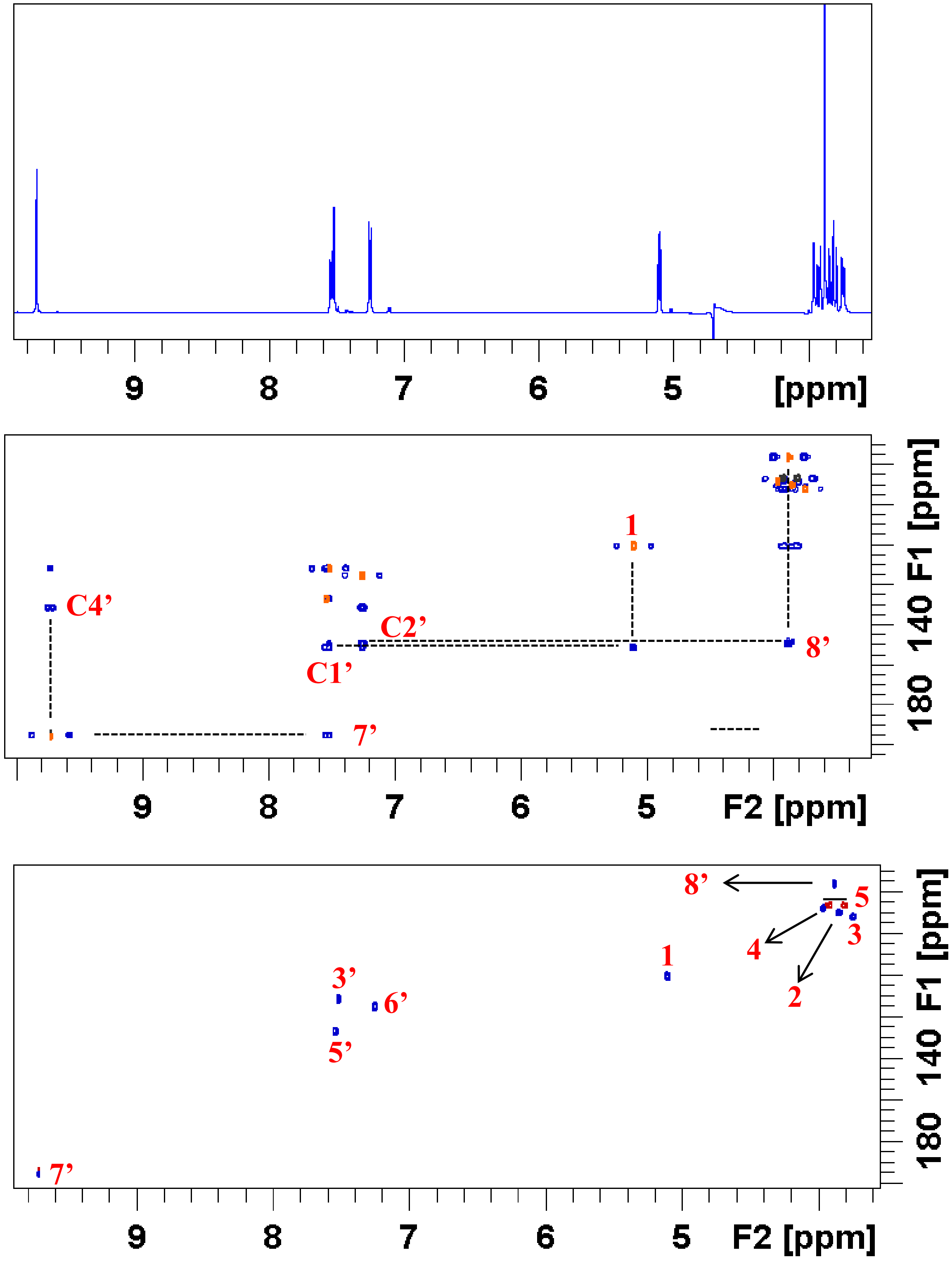
9
8
7
6
5
[ppm]
1
C4'
C2'
C1'
8'
7'
F2 [ppm]
F1 [ppm]
140
180
8'
5
3
4
2
1
3'
6'
5'
7'

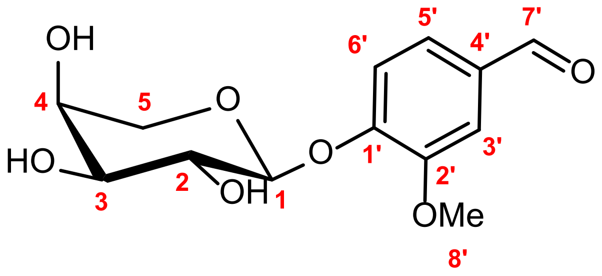

	^{1}H Chemical shift	^{13}C Chemical shift
**1**	5.10	100.28
**2**	3.85	70.07
**3**	3.74	72.12
**4**	3.95	68.03
**5**	3.80	66.35
**5**	3.92	
**1’**	---	151.09
**2’**	---	149.10
**3’**	7.52	111.54
**4’**	---	131.21
**5’**	7.54	126.74
**6’**	7.26	115.03
**7’**	9.72	195.52
**8’**	3.88	55.99

g)

(13)

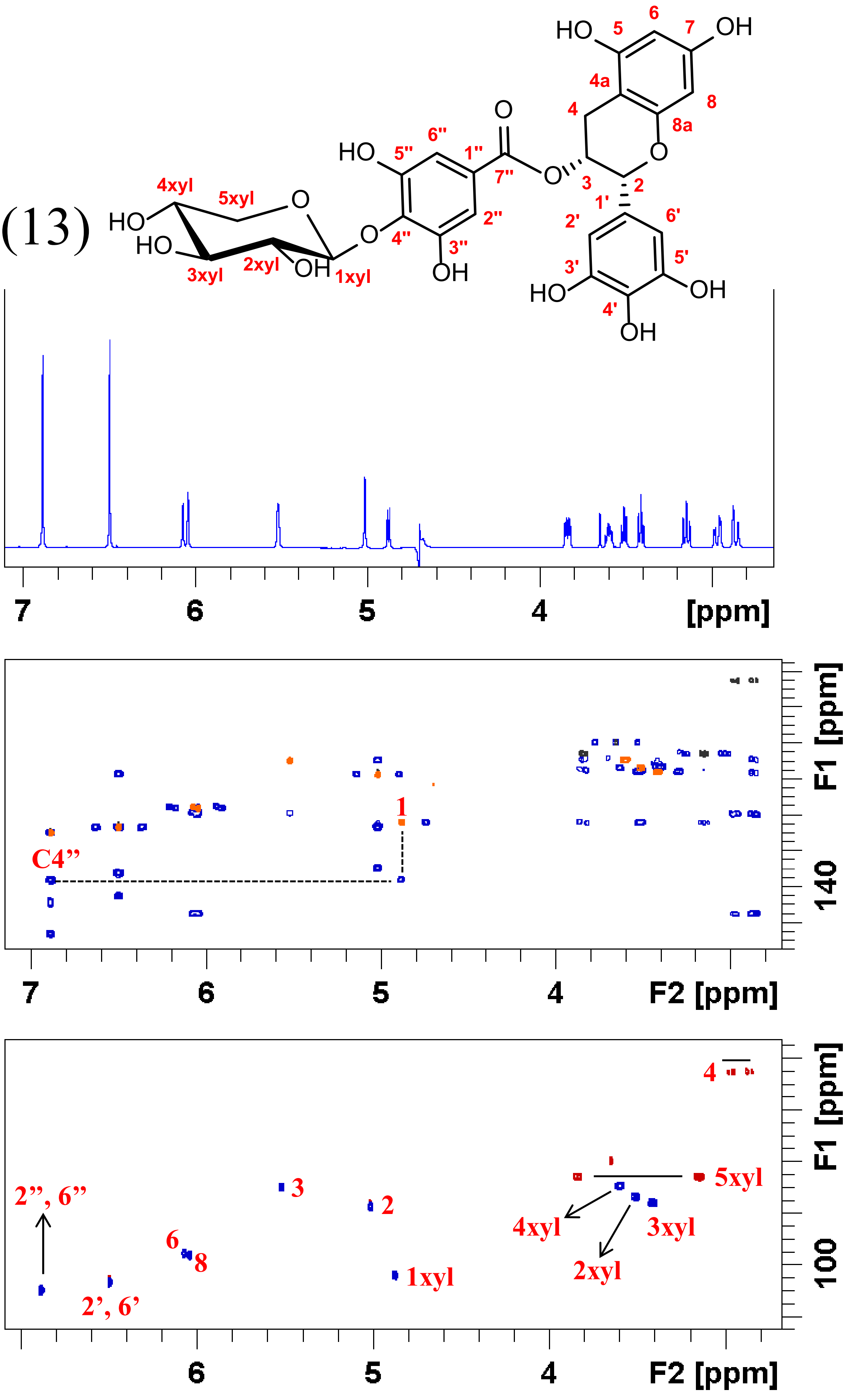
HO
OH
O
O
HO
HO
HO
OH
OH
HO
OH
OH
OH
1xyl
2xyl
3xyl
4xyl
5xyl
1"
2"
3"
4"
5"
6"
7"
2
3
4
4a
5
6
7
8
8a
1'
2'
3'
4'
5'
6'
[ppm]
F1 [ppm]
F2 [ppm]
140
100
C4"
1
4
5xyl
4xyl
3xyl
2xyl
3
2
1xyl
6
8
2", 6"
2', 6'

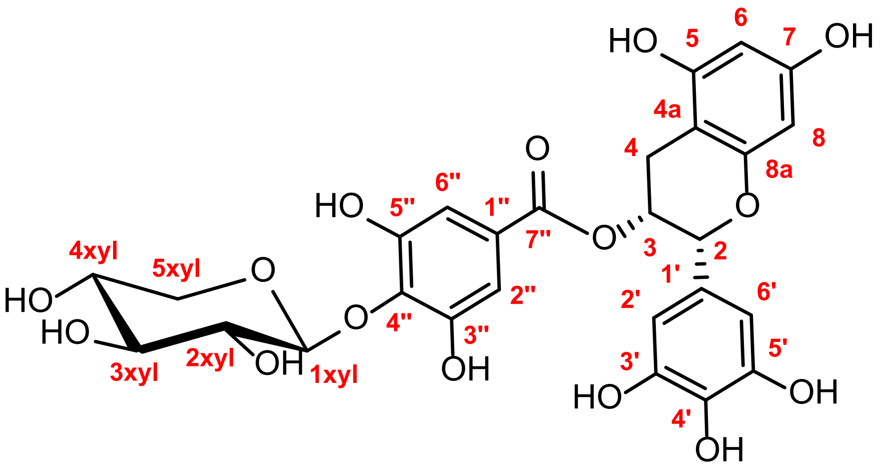

	^{1}H Chemical shift	^{13}C Chemical shift
**1 xyl**	4.87	103.8
**2 xyl**	3.51	73.33
**3 xyl**	3.41	75.42
**4 xyl**	3.60	69.20
**5 xyl**	3.15	65.54
**5 xyl**	3.85	
**2**	5.01	77.18
**3**	5.52	69.04
**4**	2.87	25.13
**4**	2.98	
**4a**	---	99.41
**5**	---	154.95
**6**	6.07	95.30
**7**	---	154.95
**8**	6.04	96.07
**8a**	---	154.95

**1’**	---	129.5
**2’**	6.50	106.46
**3’**	---	145.14
**4’**	---	132.15
**5’**	---	145.14
**6’**	6.50	106.46
**1”**	---	127.06
**2”**	6.89	109.70
**3”**	---	148.53
**4”**	---	135.99
**5”**	---	148.53
**6”**	6.89	109.70
**7”**	---	165.80

h)

(15)

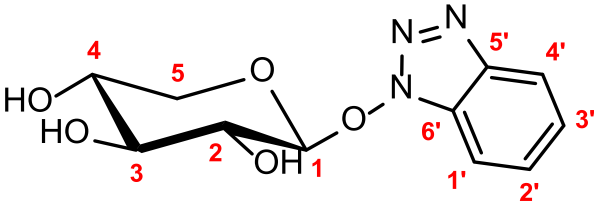
HO
HO
OH
O
N
N=N
1
2
3
4
5
1'
2'
3'
4'
5'
6'

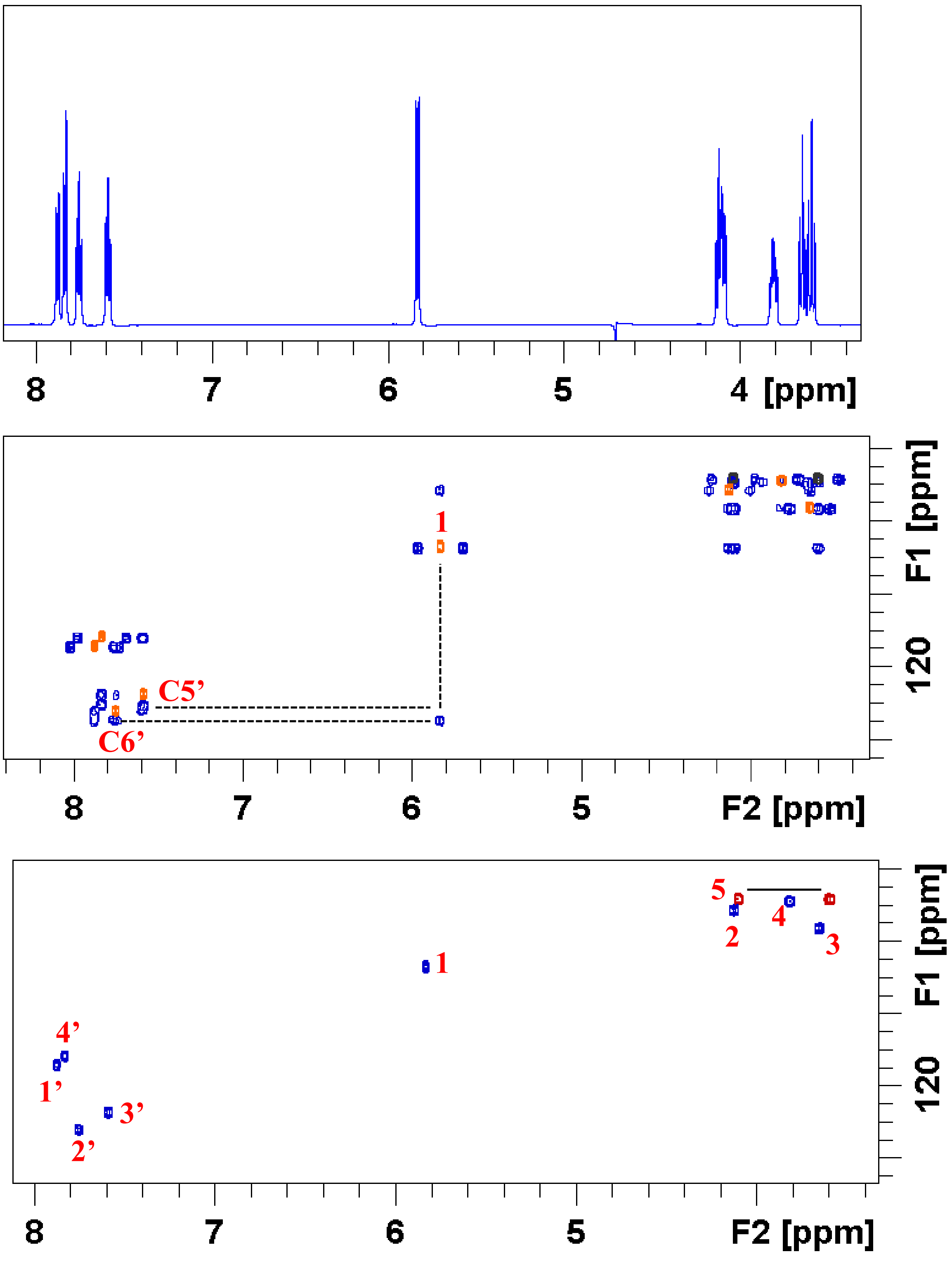
8
7
6
5
4 [ppm]
1
C5'
C6'
F1 [ppm]
120
F2 [ppm]
5
4
2
3
1
4'
1'
3'
2'

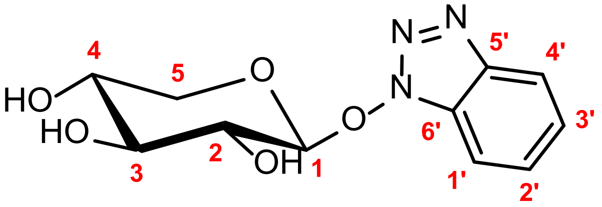

	^{1}H Chemical shift	^{13}C Chemical shift
**1**	5.82	86.80
**2**	4.10	71.19
**3**	3.64	76.08
**4**	3.81	68.66
**5**	3.59	68.03
**5**	4.09	
**1’**	7.87	114.40
**2’**	7.75	132.20
**3’**	7.58	127.39
**4’**	7.83	111.53
**5’**	---	129.88
**6’**	---	134.55

i)

(17) 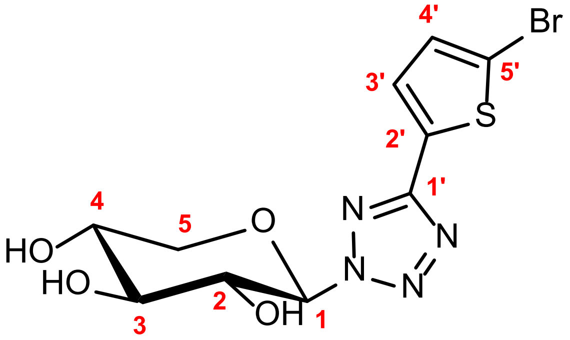

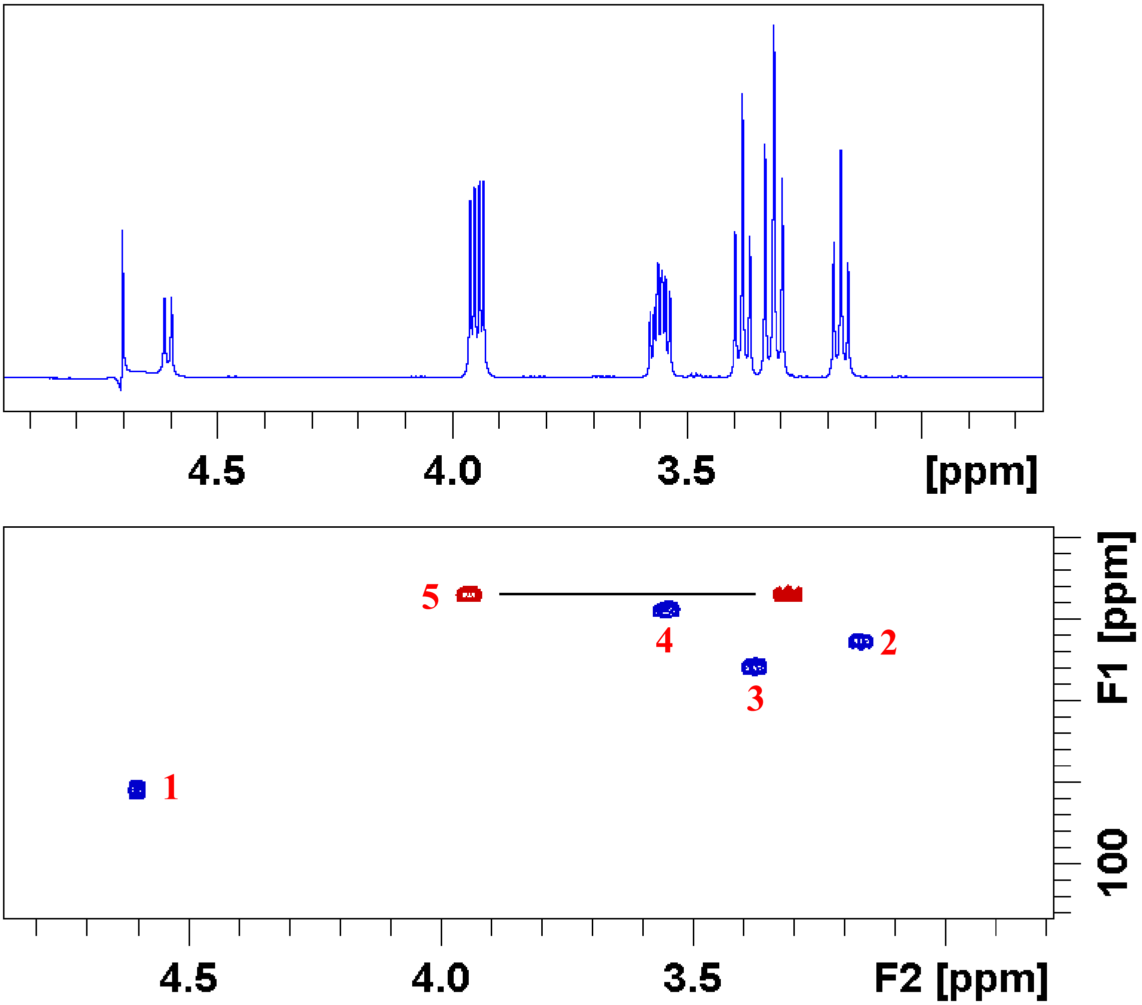

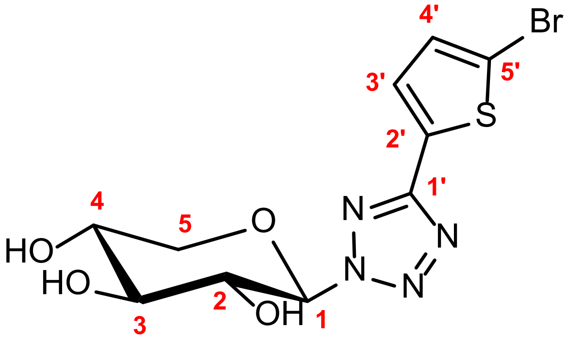

	^{1}H Chemical shift	^{13}C Chemical shift
**1**	5.95	89.83
**2**	4.09	71.80
**3**	3.64	75.91
**4**	3.80	68.70
**5**	3.59	68.01
**5**	4.11	
**1’**	---	160.71
**2’**	---	129.95
**3’**	7.58	129.47
**4’**	7.22	131.53
**5’**	---	116.02

j)

(19)

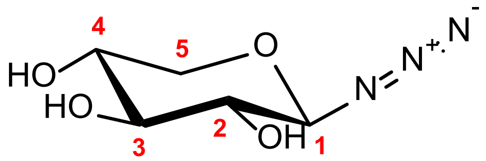
HO
HO
OH
O
N
1
2
3
4
5

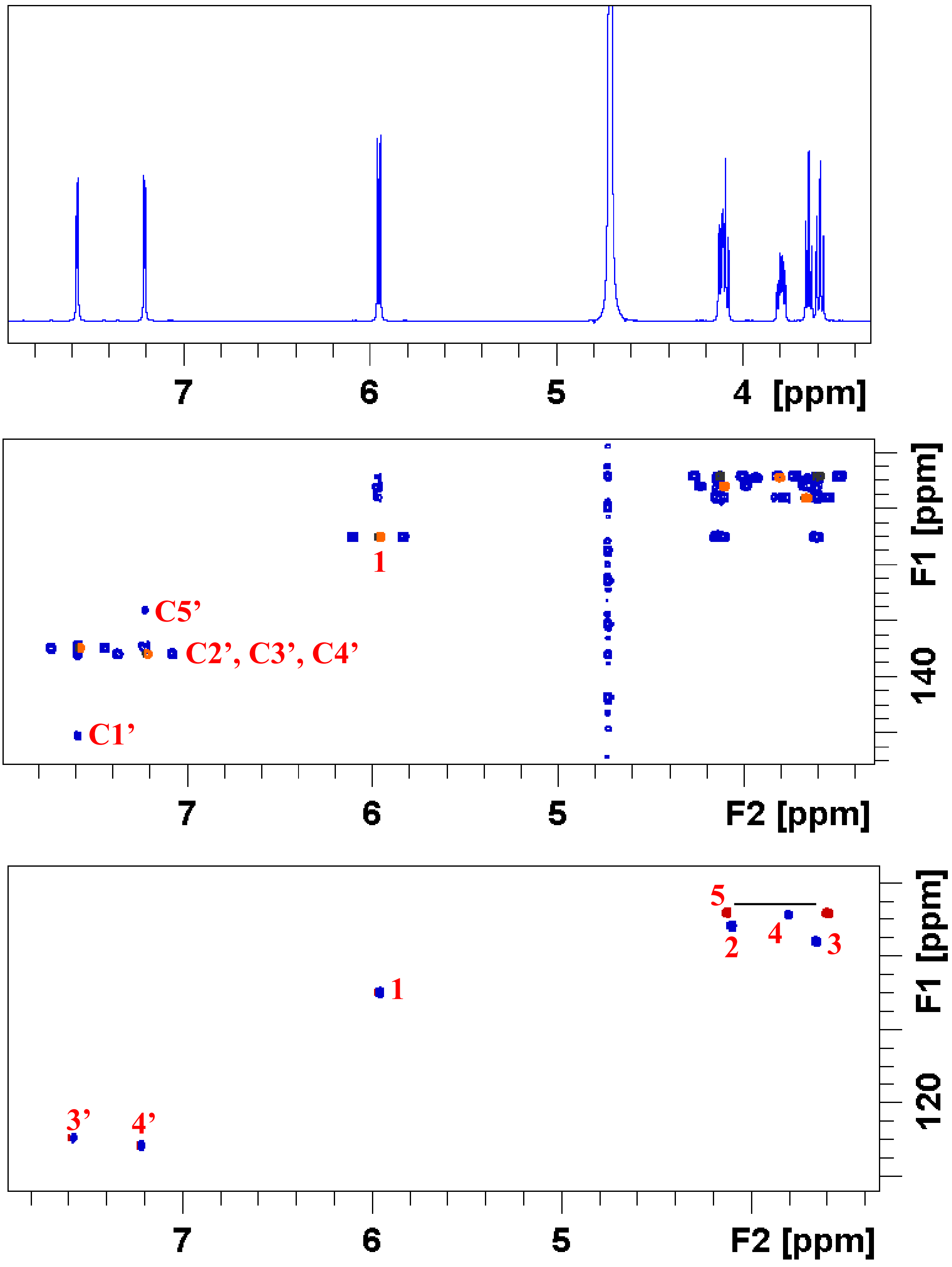
7
6
5
4
[ppm]
1
C5’
C2’, C3’, C4’
C1’
140
F1 [ppm]
F2 [ppm]
5
4
2
3
1
3’
4’
120

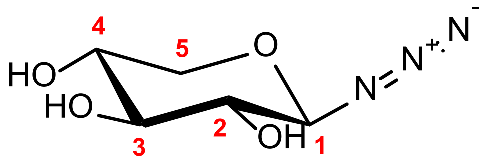

<table>
<tr><th></th><th>¹H Chemical shift</th><th>¹³C Chemical shift</th></tr>
<tr><td>1</td><td>4.60</td><td>90.90</td></tr>
<tr><td>2</td><td>3.17</td><td>72.74</td></tr>
<tr><td>3</td><td>3.38</td><td>75.80</td></tr>
<tr><td>4</td><td>3.56</td><td>68.96</td></tr>
<tr><td>5</td><td>3.31</td><td rowspan="2">66.90</td></tr>
<tr><td>5</td><td>3.95</td></tr>
</table>

k)

(21)

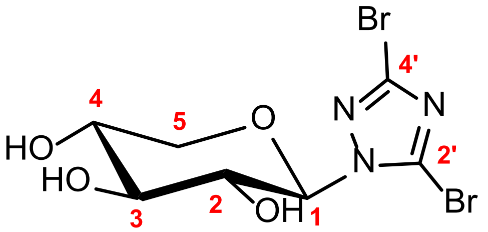
Br
4'
N
N
N
2'
Br
O
HO
HO
OH
1
2
3
4
5

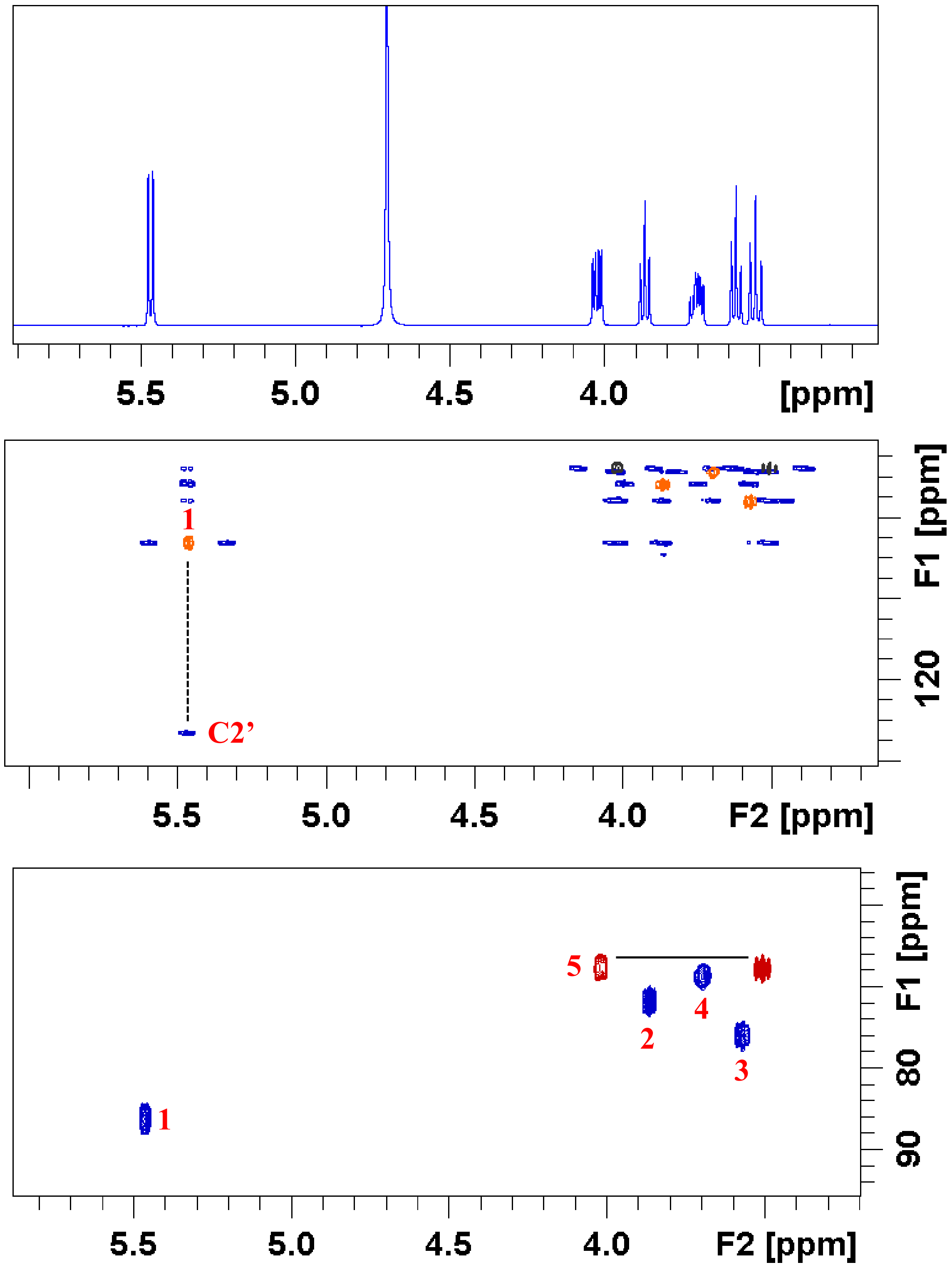
5.5
5.0
4.5
4.0
[ppm]
1
C2'
F1 [ppm]
120
F2 [ppm]
5
2
4
3
80
90

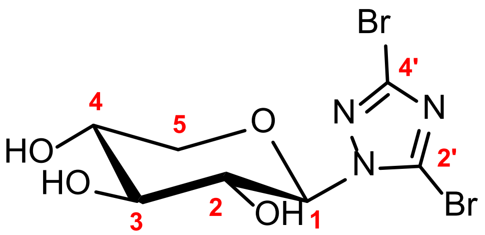

	^{1}H Chemical shift	^{13}C Chemical shift
**1**	5.46	86.49
**2**	3.86	72.16
**3**	3.57	76.29
**4**	3.69	68.96
**5**	3.51	67.85
**5**	4.02	
**2’**	---	133.28
**4’**	---	141.06

1)

(23)

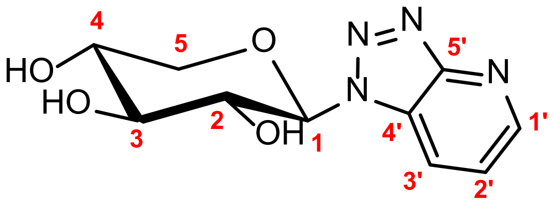

HO
HO
OH
O
N
N=N
4
5
3
2
1
5'
4'
3'
2'
1'

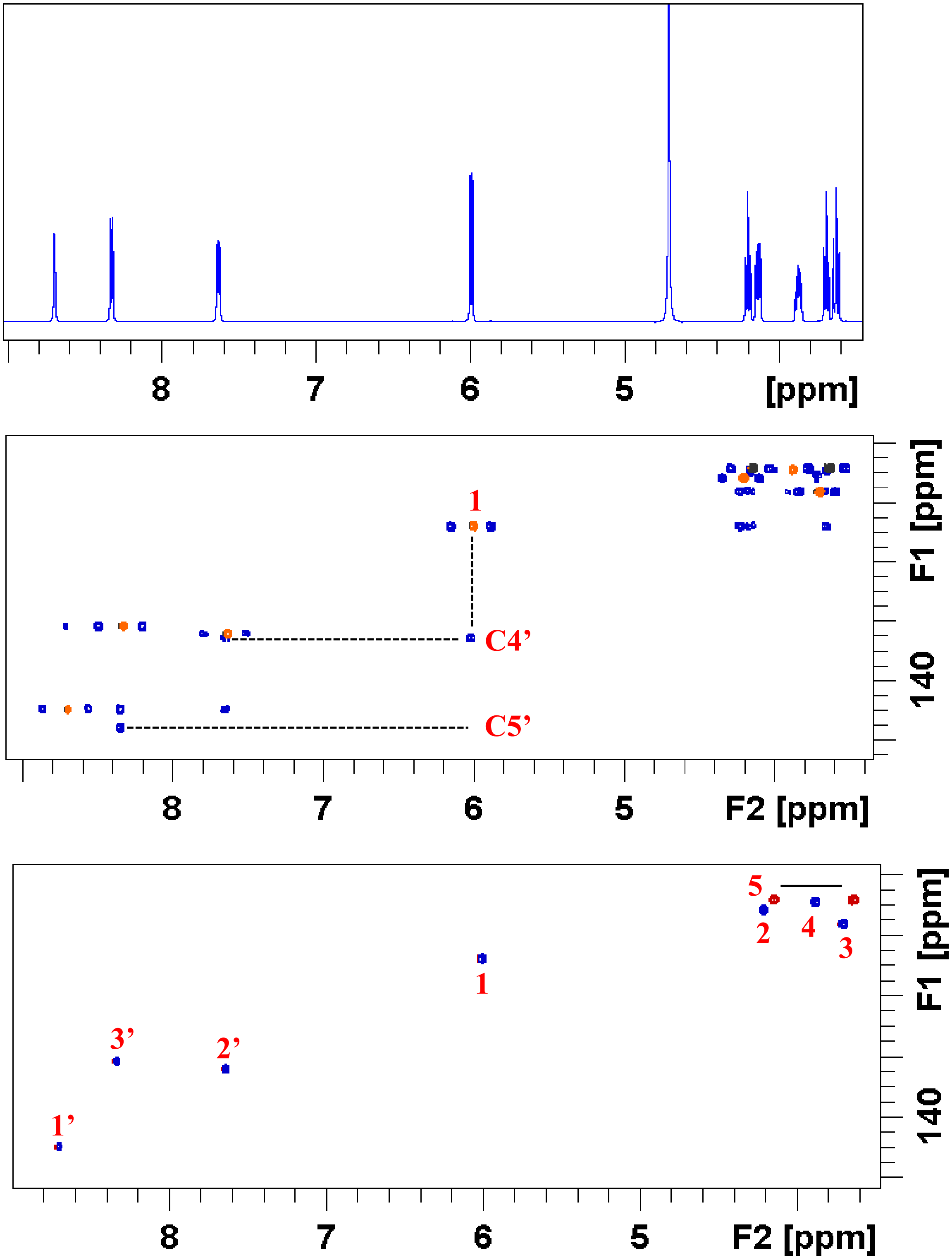

[ppm]
F1 [ppm]
F2 [ppm]
1
C4’
C5’
5
2
4
3
3’
2’
1’
140
8
7
6
5

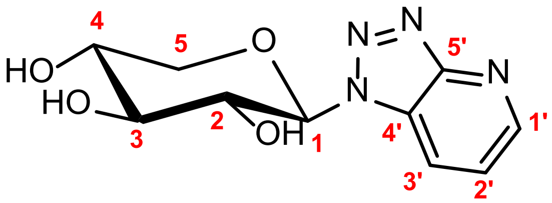

	^{1}H Chemical shift	^{13}C Chemical shift
**1**	6.00	87.66
**2**	4.20	71.43
**3**	3.69	76.13
**4**	3.88	68.76
**5**	3.62	68.02
**5**	4.13	
**1’**	8.70	149.56
**2’**	7.63	123.95
**3’**	8.33	121.47
**4’**	---	125.68
**5’**	---	155.69

m)

(25) 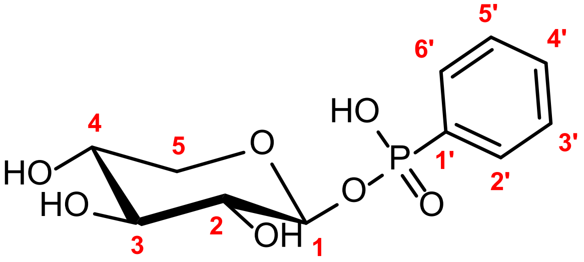
HO
HO
OH
O
HO
P
O
O
1
2
3
4
5
1'
2'
3'
4'
5'
6'

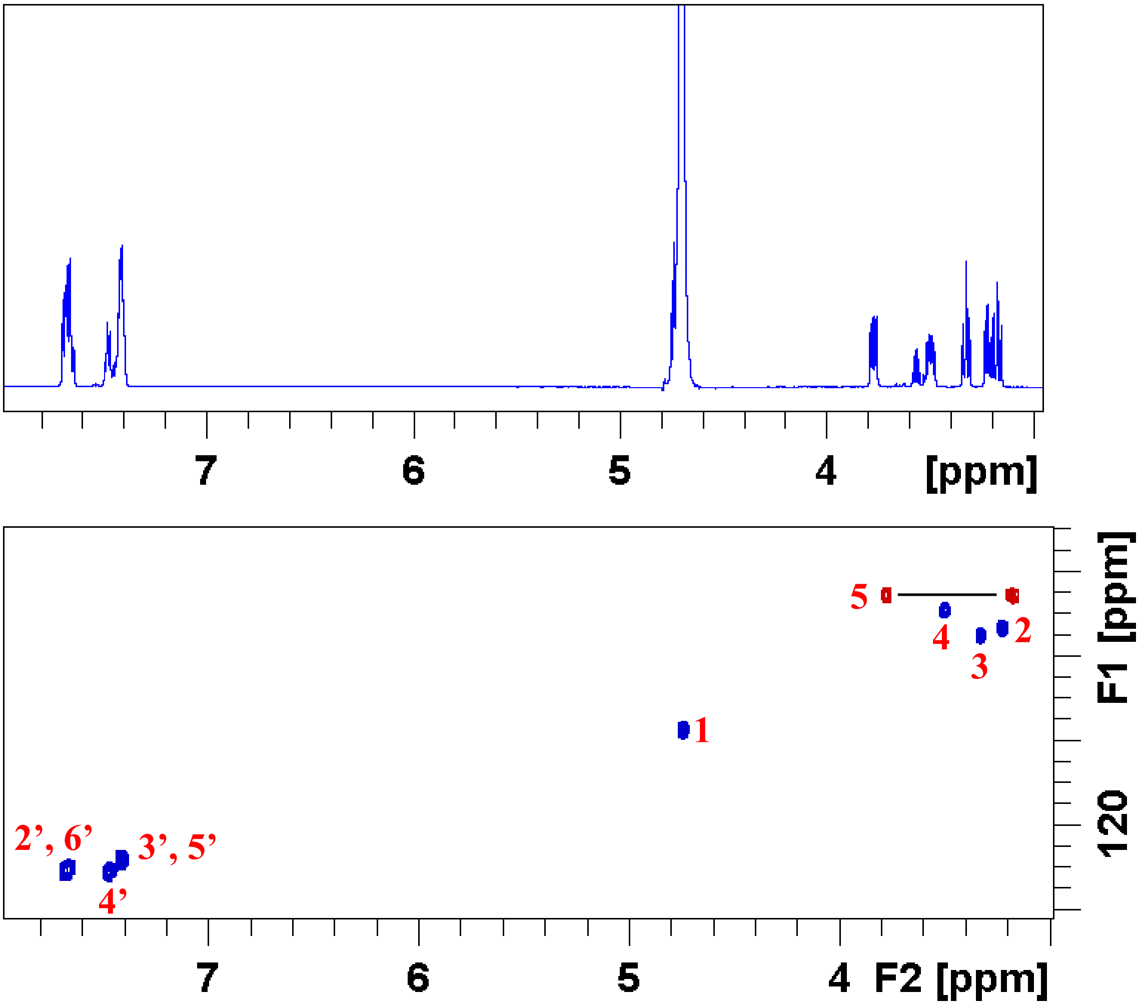
7
6
5
4
[ppm]
5
4
2
3
1
2’, 6’
3’, 5’
4’
120
F1 [ppm]
7
6
5
4 F2 [ppm]

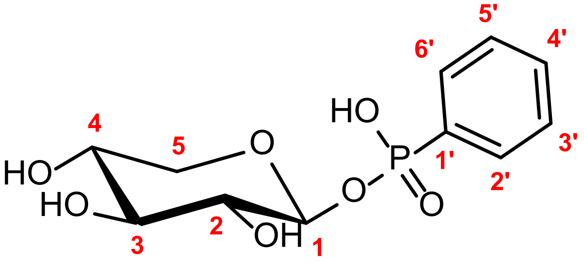

	^{1}H Chemical shift	^{13}C Chemical shift
**1**	4.74	97.59
**2**	3.22	73.47
**3**	3.32	75.19
**4**	3.50	69.10
**5**	3.17	65.55
**5**	3.76	
**2’**	7.68	130.53
**3’**	7.41	128.23
**4’**	7.48	130.88
**5’**	7.41	128.23
**6’**	7.68	130.53

n)

(27) 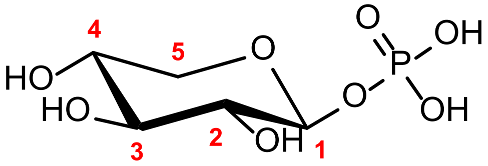

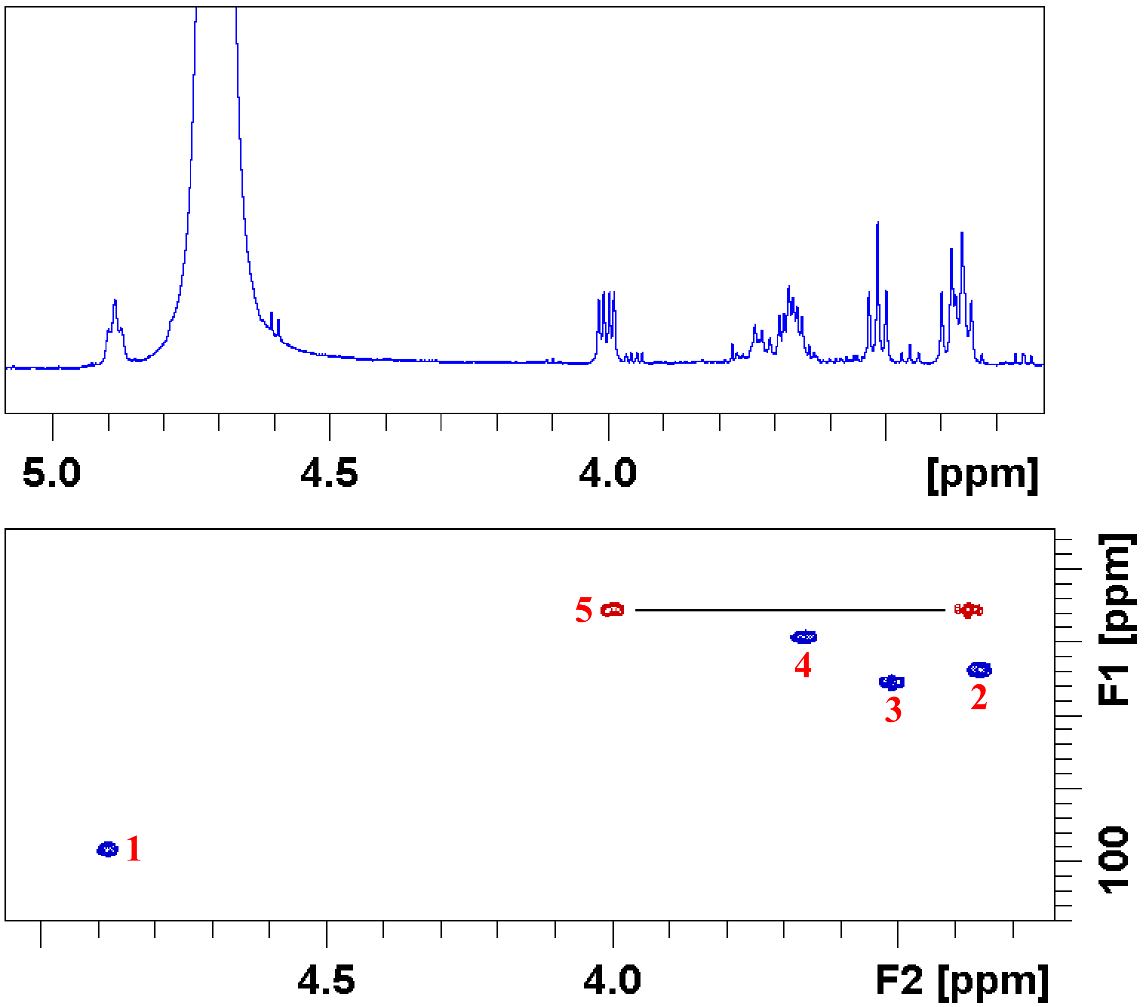

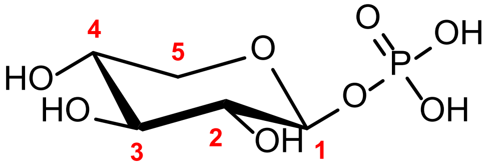

	^{1}H Chemical shift	^{13}C Chemical shift
**1**	4.88	98.35
**2**	3.36	73.70
**3**	3.51	75.57
**4**	3.66	69.24
**5**	3.38	65.57
**5**	4.00	

o)

(29) 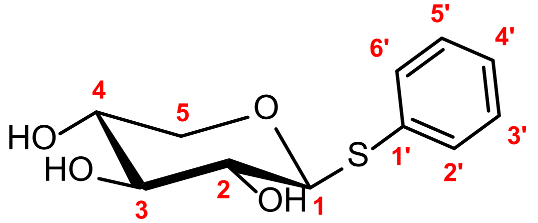

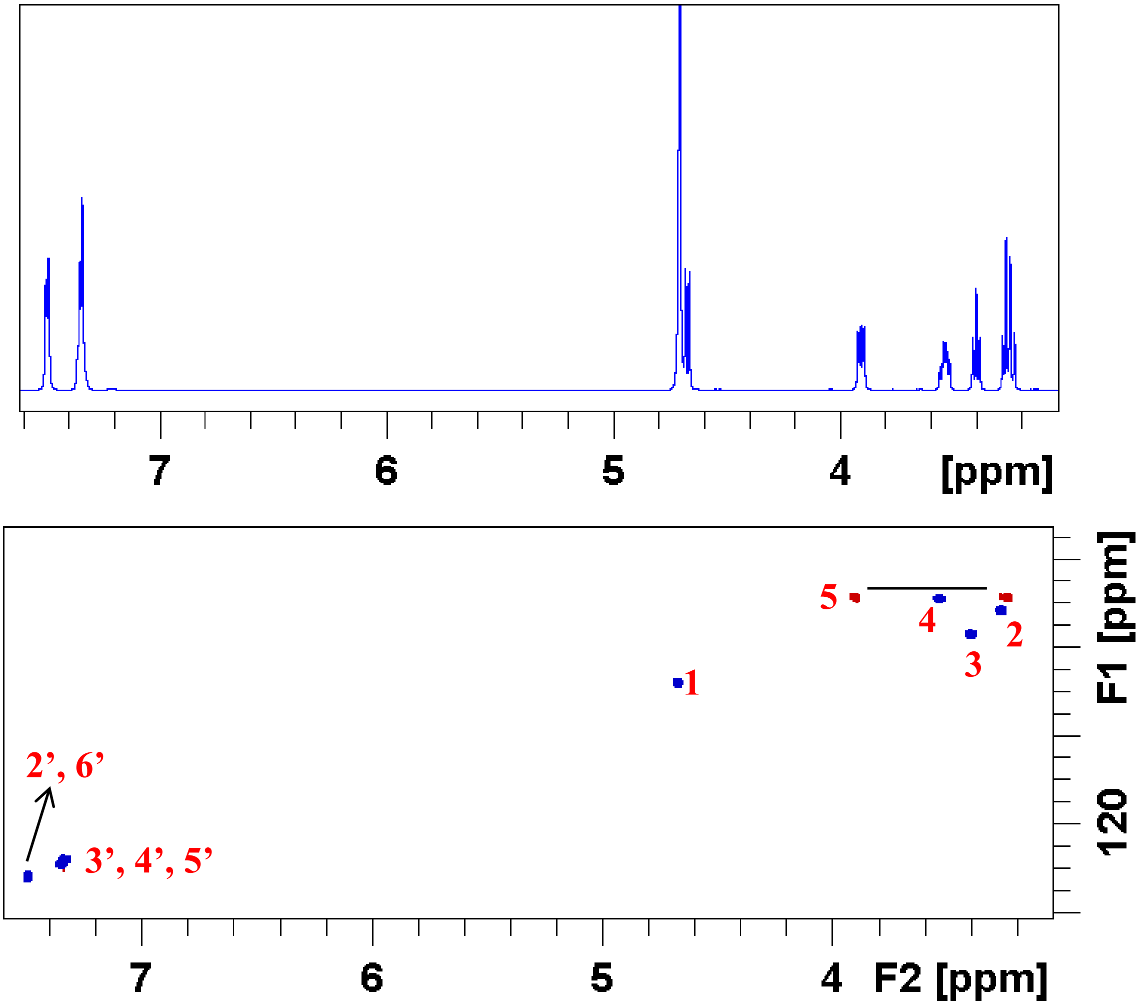

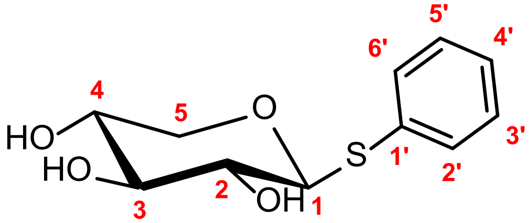

	^{1}H Chemical shift	^{13}C Chemical shift
**1**	4.66	88.06
**2**	3.26	71.67
**3**	3.40	77.02
**4**	3.53	69.05
**5**	3.24	68.76
**5**	3.90	
**2’**	7.49	132.08
**3’**	7.35	129.21
**4’**	7.34	128.55
**5’**	7.35	129.21
**6’**	7.49	132.08

p)

(31)

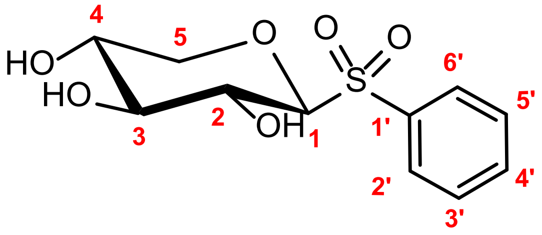
HO
HO
OH
O
O
O
S
1
2
3
4
5
1'
2'
3'
4'
5'
6'

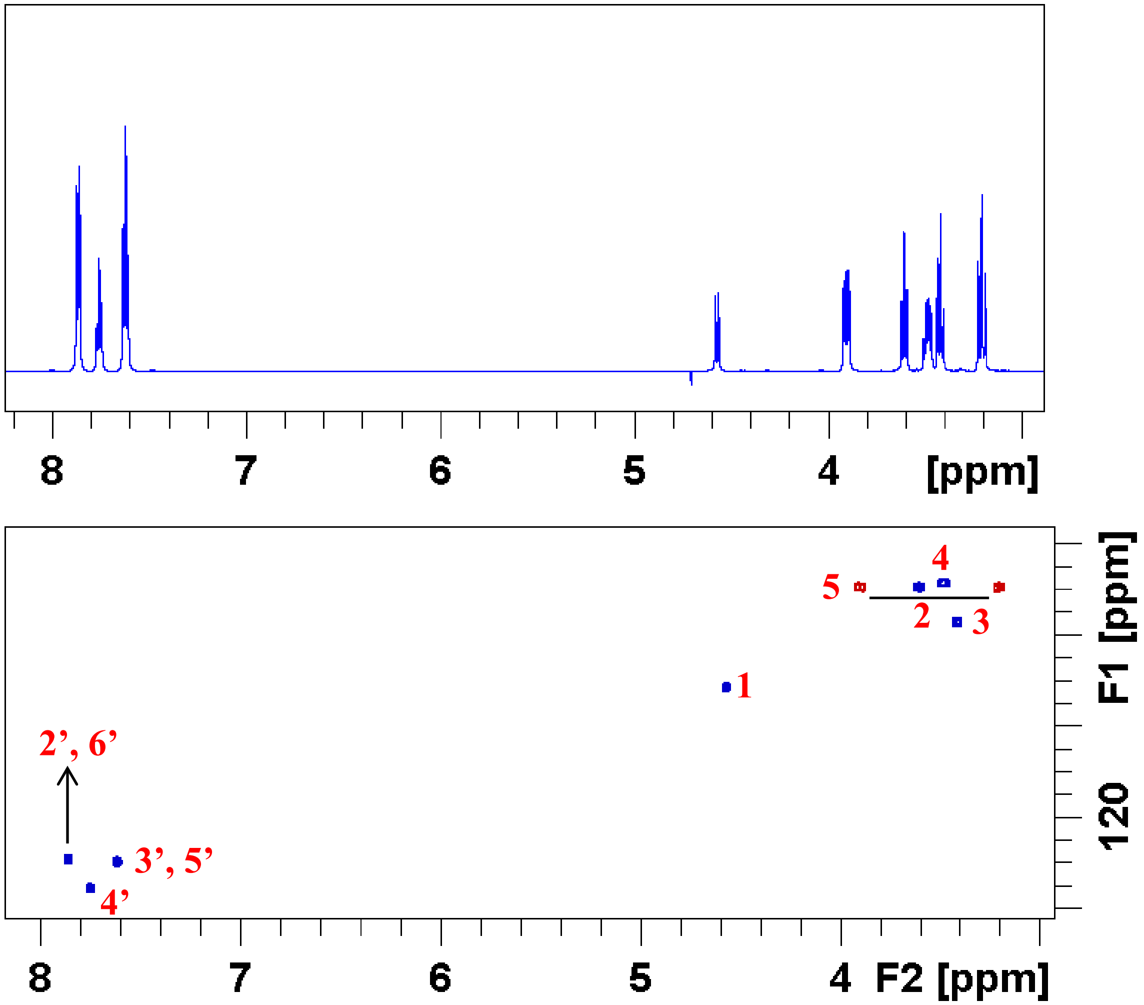
8
7
6
5
4
[ppm]
5
4
2
3
1
2', 6'
3', 5'
4'
120
F1 [ppm]
F2 [ppm]

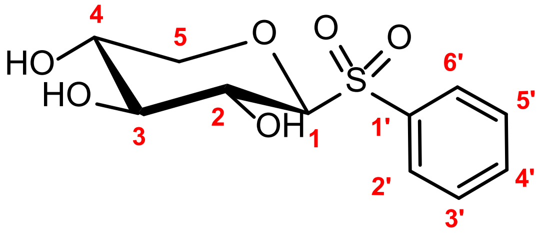

	^{1}H Chemical shift	^{13}C Chemical shift
**1**	4.57	91.29
**2**	3.60	69.28
**3**	3.42	76.85
**4**	3.48	68.31
**5**	3.20	69.31
**5**	3.90	
**2'**	7.86	129.10
**3'**	7.62	129.56
**4'**	7.75	135.39
**5'**	7.62	129.56
**6'**	7.86	129.10

q)

(33)

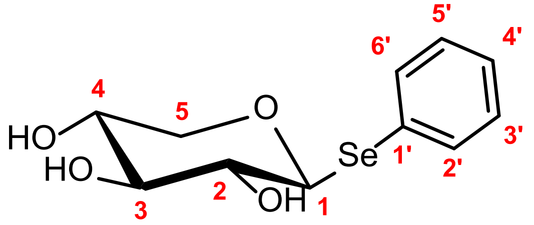
HO
HO
OH
O
Se
1
2
3
4
5
1'
2'
3'
4'
5'
6'

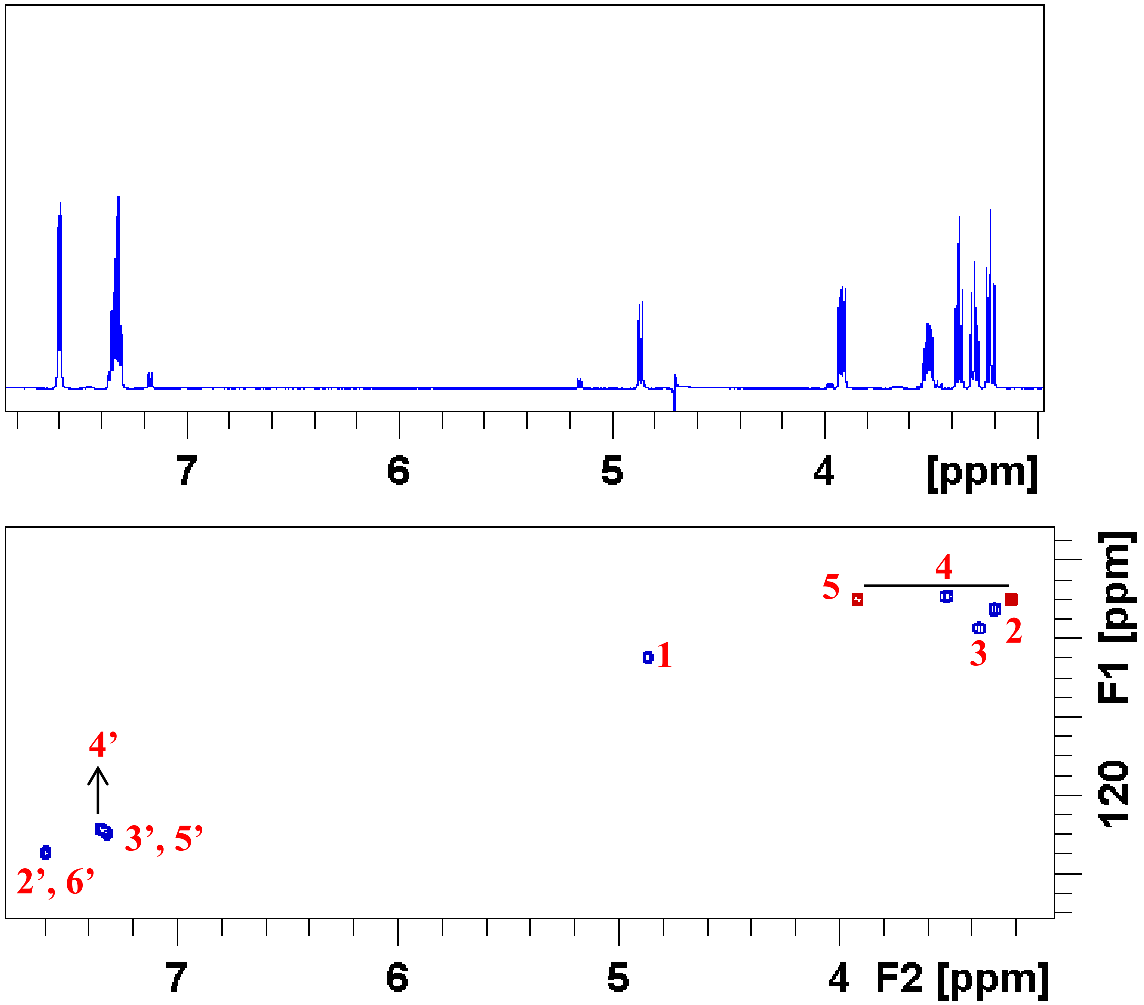
7
6
5
4
[ppm]
F1 [ppm]
120
F2 [ppm]
1
2
3
4
5
4’
3’, 5’
2’, 6’

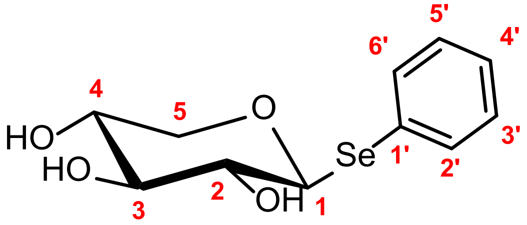

	^{1}H Chemical shift	^{13}C Chemical shift
**1**	4.86	84.62
**2**	3.29	72.25
**3**	3.37	77.02
**4**	3.51	68.93
**5**	3.22	69.86
**5**	3.92	
**1'**	---	126.54
**2'**	7.59	134.52
**3'**	7.32	129.40
**4'**	7.35	128.65
**5'**	7.32	129.40
**6'**	7.59	134.52

r)

(35)

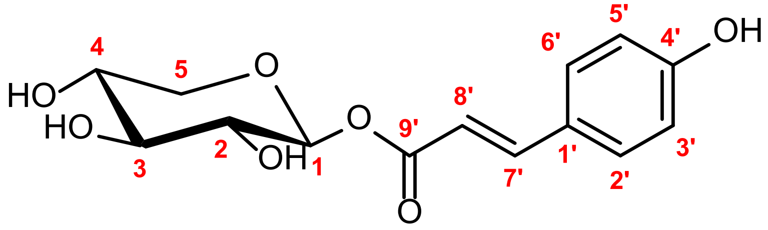

HO
HO
OH
OH
O
O
O
1
2
3
4
5
1'
2'
3'
4'
5'
6'
7'
8'
9'

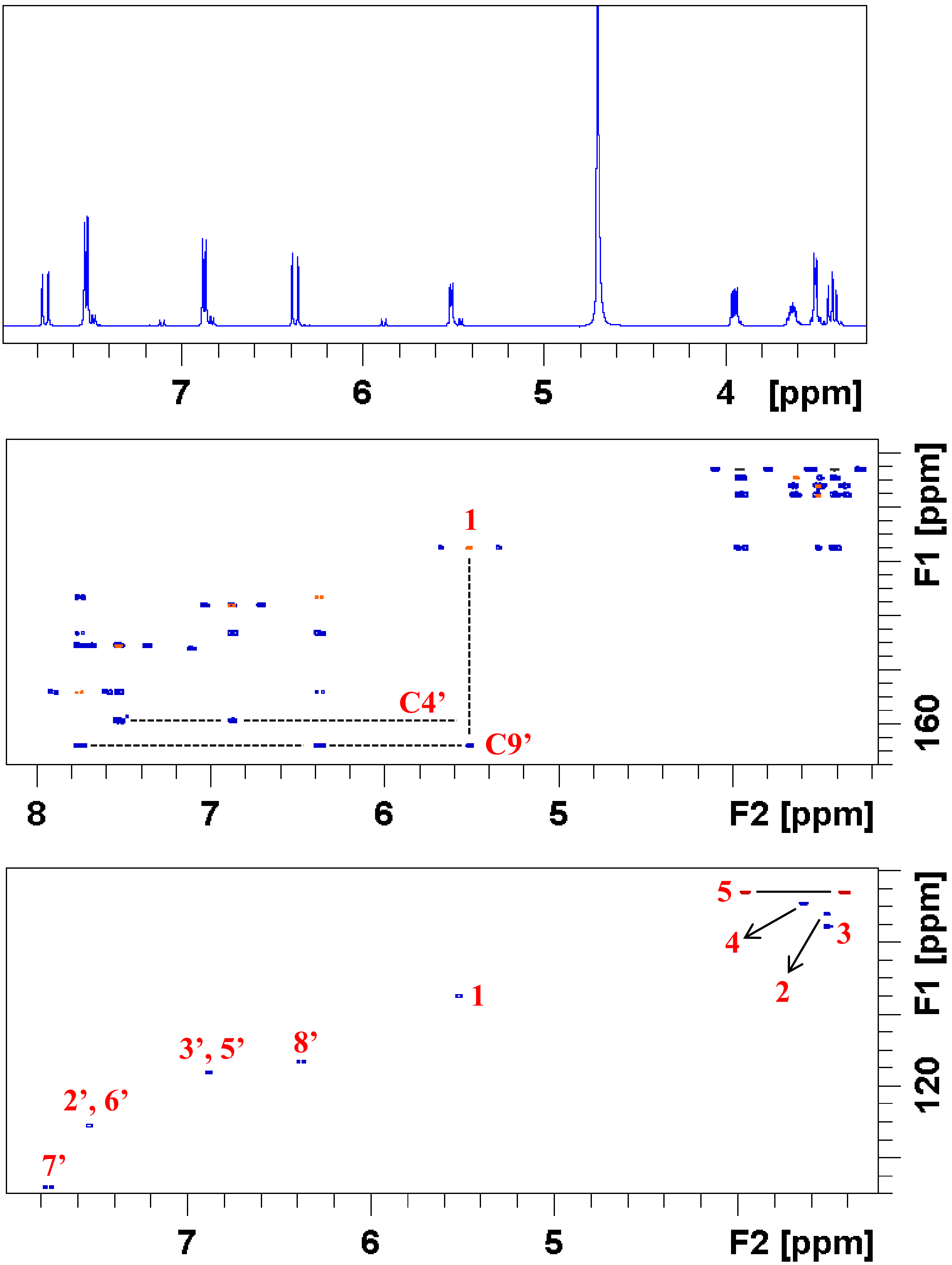

7
6
5
4
[ppm]
1
C4'
C9'
F1 [ppm]
160
8
7
6
5
F2 [ppm]
5
4
3
2
1
3', 5'
8'
2', 6'
7'
F1 [ppm]
120
7
6
5
F2 [ppm]

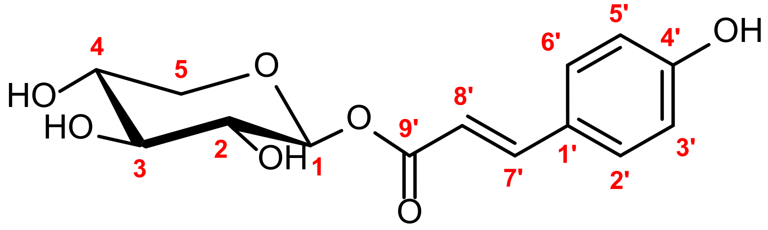

	^{1}H Chemical shift	^{13}C Chemical shift
**1**	5.51	94.71
**2**	3.50	71.85
**3**	3.51	75.39
**4**	3.63	69.01
**5**	3.40	65.83
**5**	3.95	
**2'**	7.53	130.76
**3'**	6.88	115.84
**5'**	6.88	115.84
**6'**	7.53	130.76
**7'**	7.76	147.95
**8'**	6.38	113.05

s)

(36)

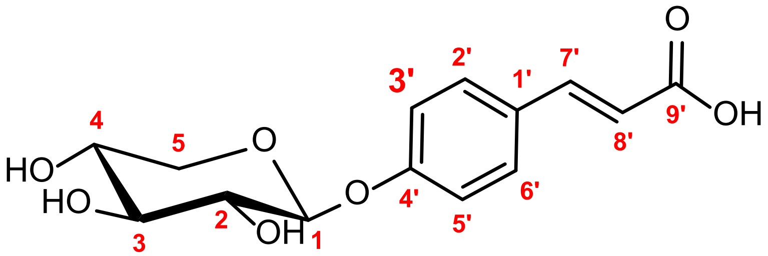

HO
HO
OH
O
OH
1
2
3
4
5
1'
2'
3'
4'
5'
6'
7'
8'
9'

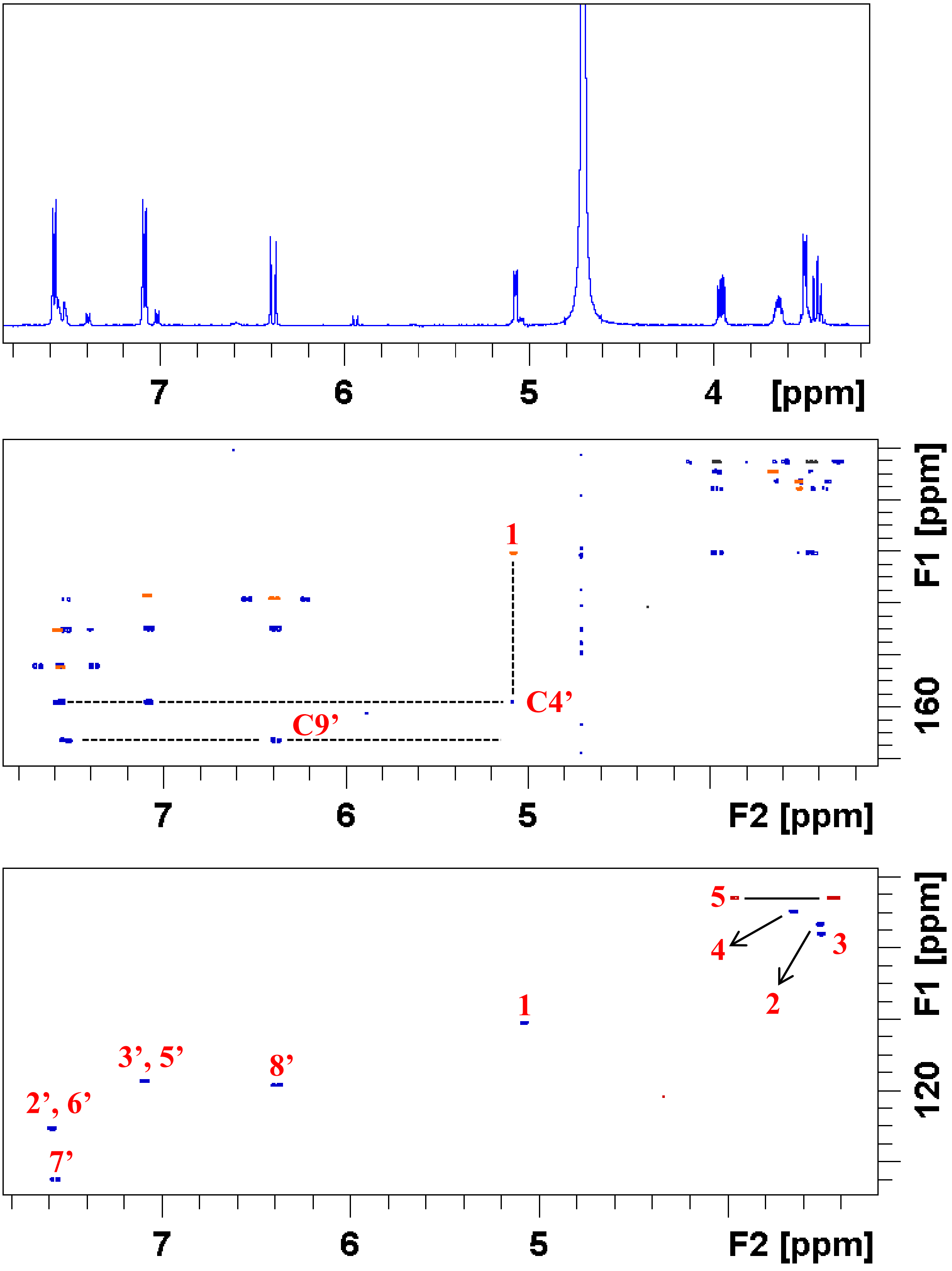

7
6
5
4
[ppm]
1
C4’
C9’
160
F1 [ppm]
F2 [ppm]
5
4
3
2
1
3’, 5’
8’
2’, 6’
7’
120
F1 [ppm]
F2 [ppm]

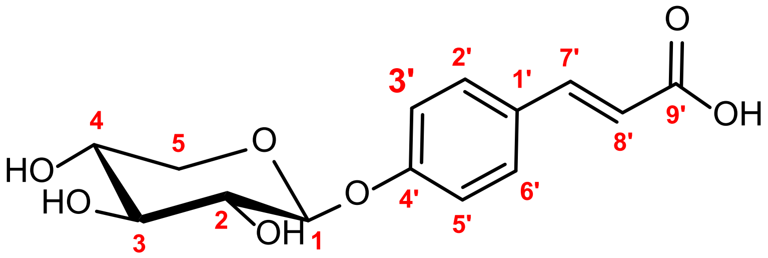

	**^{1}H Chemical shift**	**^{13}C Chemical shift**
**1**	5.08	100.27
**2**	3.51	72.74
**3**	3.50	75.40
**4**	3.66	68.99
**5**	3.44	65.18
**5**	3.97	
**2’**	7.58	129.98
**3’**	7.08	116.75
**5’**	7.08	116.75
**6’**	7.58	129.98
**7’**	7.58	144.25
**8’**	6.41	117.65

t)

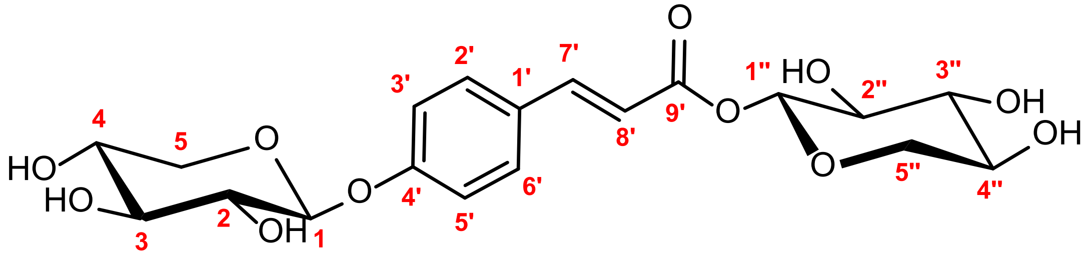

O
HO
OH
OH
HO
HO
OH

(37)

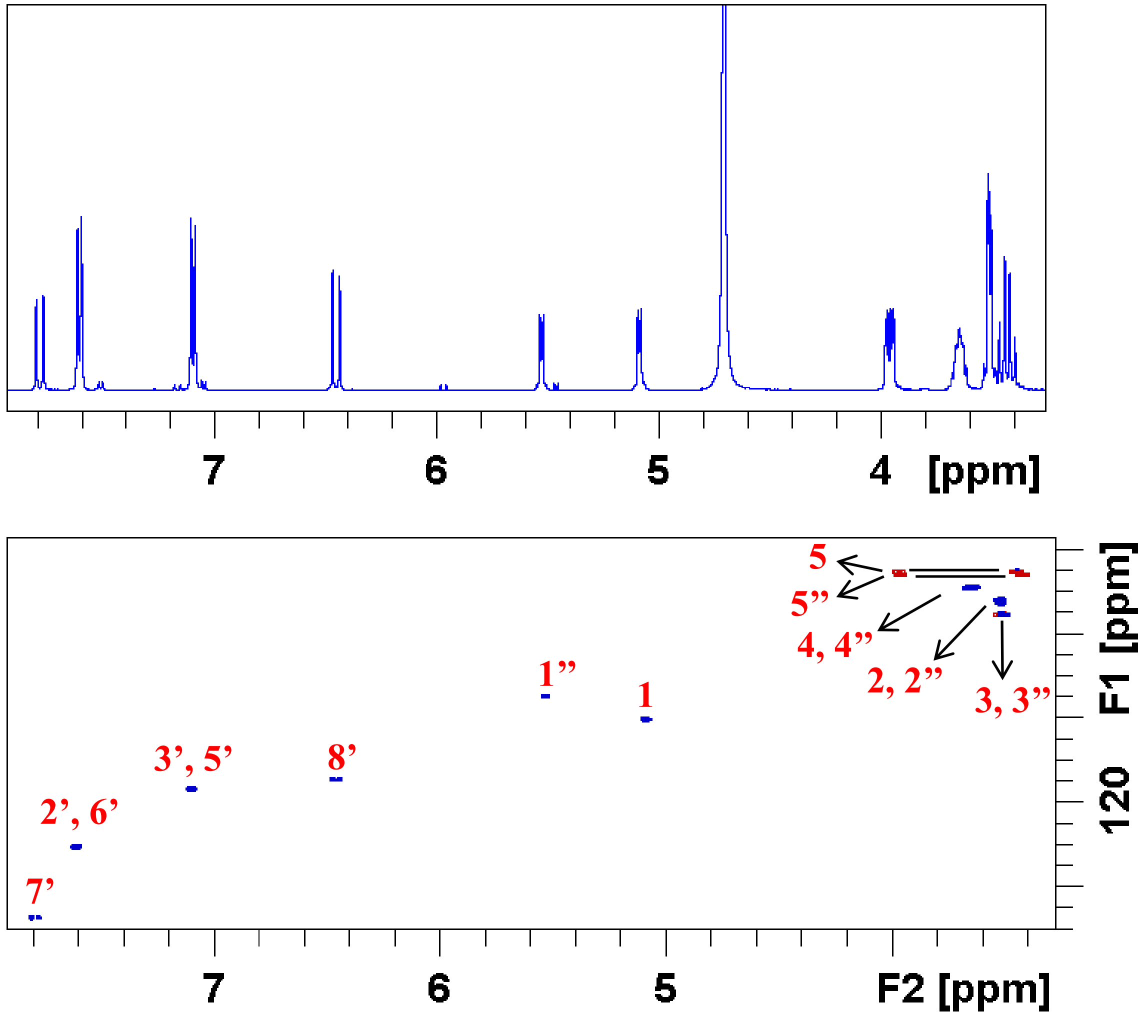

7
6
5
4
[ppm]
5
5”
4, 4”
2, 2”
3, 3”
1”
1
3’, 5’
8’
2’, 6’
7’
F1 [ppm]
120
7
6
5
F2 [ppm]

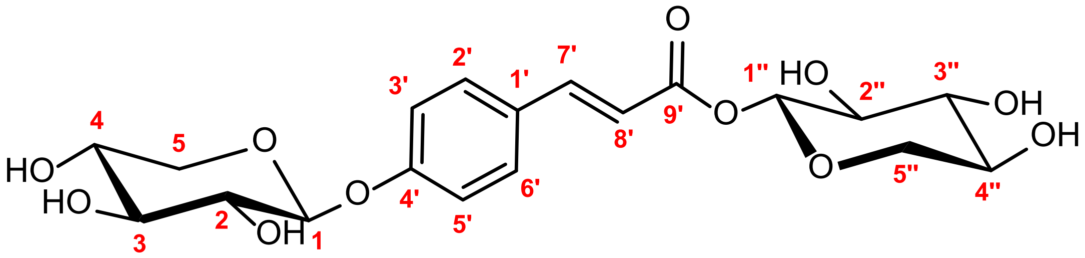

	^{1}H Chemical shift	^{13}C Chemical shift
**1**	5.08	100.17
**2**	3.52	72.70
**3**	3.51	75.47
**4**	3.64	69.03
**5**	3.44	65.27
**5**	3.96	
**2’**	7.6	130.47
**3’**	7.09	116.75
**5’**	7.09	116.75
**6’**	7.6	130.47
**7’**	7.77	147.39
**8’**	6.44	114.73

**1”**	5.53	94.81
**2”**	3.51	71.85
**3”**	3.51	75.47
**4”**	3.64	69.03
**5”**	3.44	65.82
**5”**	3.97	

u)

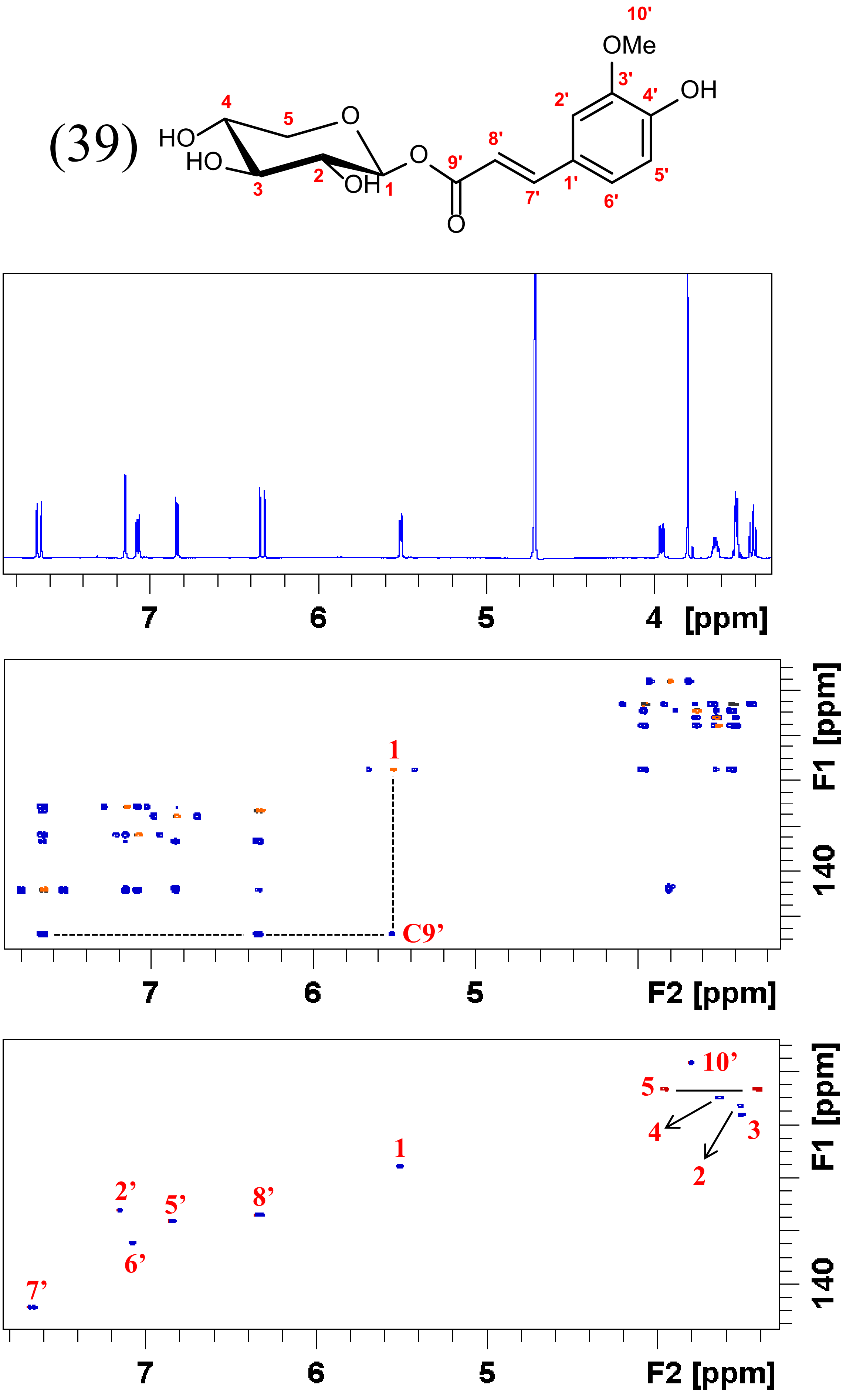
(39)
10'
OMe
3'
OH
4'
2'
4
HO
5
O
8'
HO
9'
O
2
OH
1
3
7'
1'
5'
6'
O
7
6
5
4
[ppm]
1
C9'
F1 [ppm]
140
F2 [ppm]
10'
5
4
3
2
2'
5'
8'
6'
7'

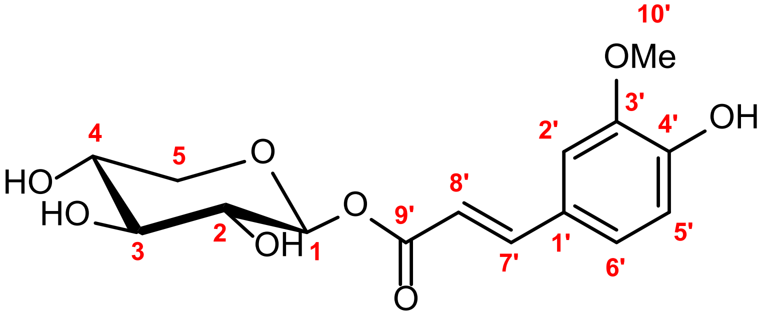

	^{1}H Chemical shift	^{13}C Chemical shift
**1**	5.51	94.77
**2**	3.50	71.90
**3**	3.50	75.35
**4**	3.63	69.01
**5**	3.41	65.80
**5**	3.95	
**1’**	---	126.69
**2’**	7.15	111.36
**3’**	---	147.60
**4’**	---	147.60
**5’**	6.84	115.53
**6’**	7.07	123.76
**7’**	7.66	147.97
**8’**	6.35	113.18
**9’**	---	167.71
**10’**	3.80	55.82

v)

(40)

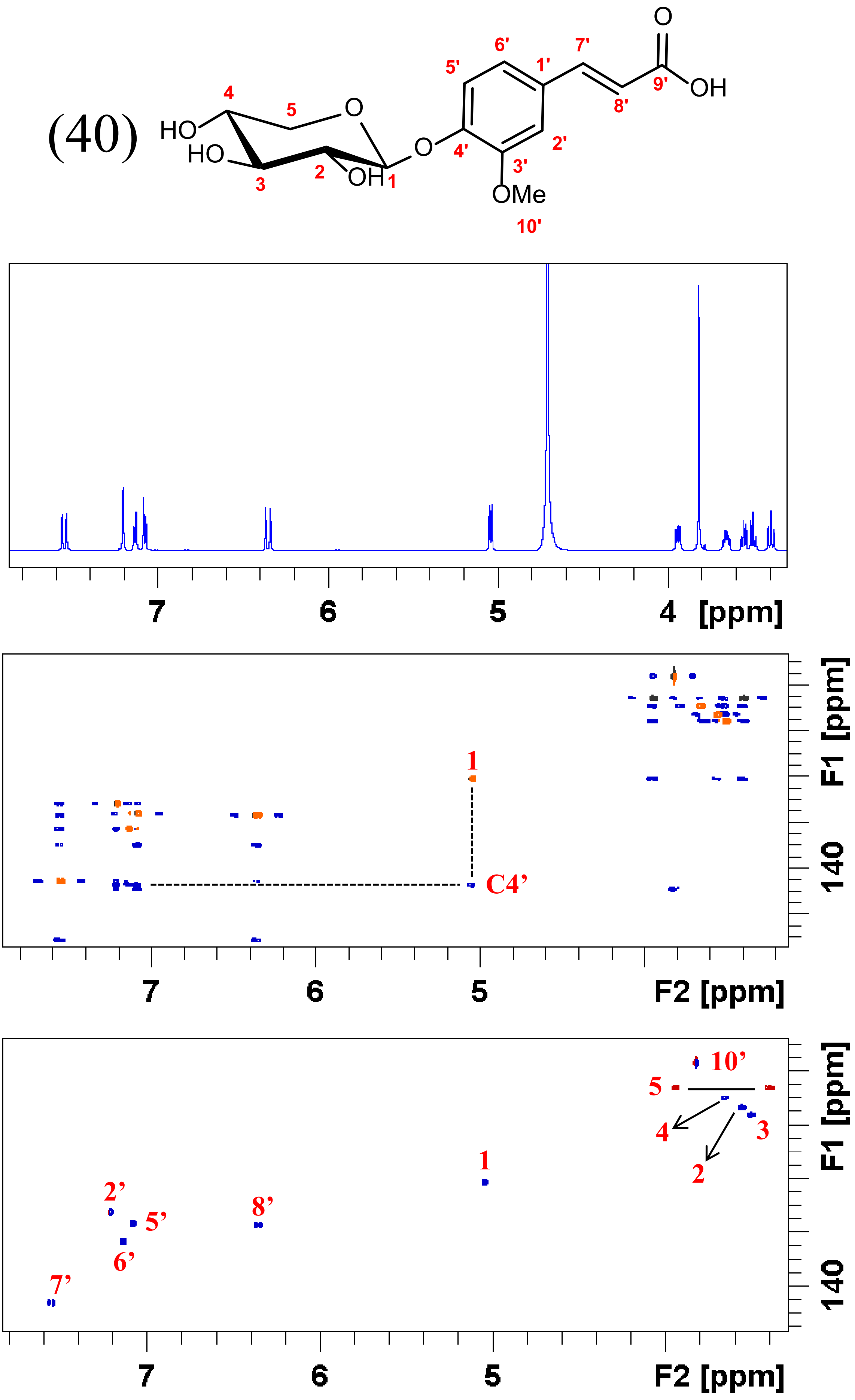
HO
HO
OH
O
O
OMe
O
OH
1
2
3
4
5
1'
2'
3'
4'
5'
6'
7'
8'
9'
10'
7
6
5
4
[ppm]
1
C4'
F1 [ppm]
140
F2 [ppm]
10'
5
4
3
2
1
2'
5'
6'
7'
8'

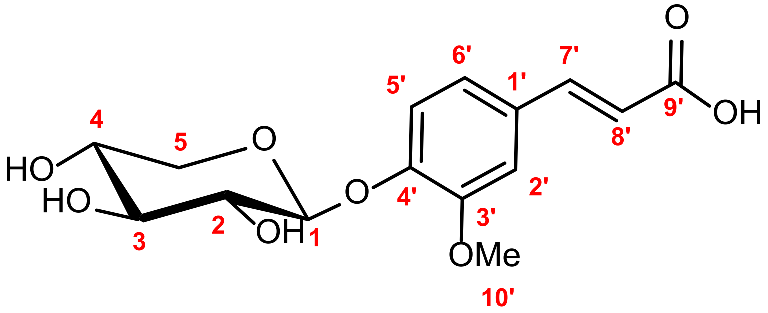

	^{1}H Chemical shift	^{13}C Chemical shift
**1**	5.04	100.74
**2**	3.54	72.71
**3**	3.48	75.41
**4**	3.65	69.07
**5**	3.39	65.35
**5**	3.95	
**1’**	---	129.76
**2’**	7.21	111.60
**3’**	---	148.97
**4’**	---	147.19
**5’**	7.07	115.90
**6’**	7.13	122.57
**7’**	7.55	145.57
**8’**	6.35	116.57
**9’**	---	171.28
**10’**	3.82	55.89

w)

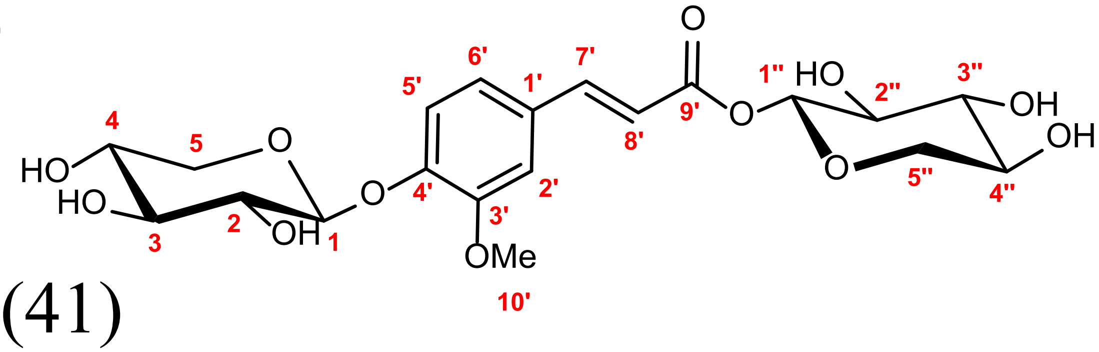

(41)

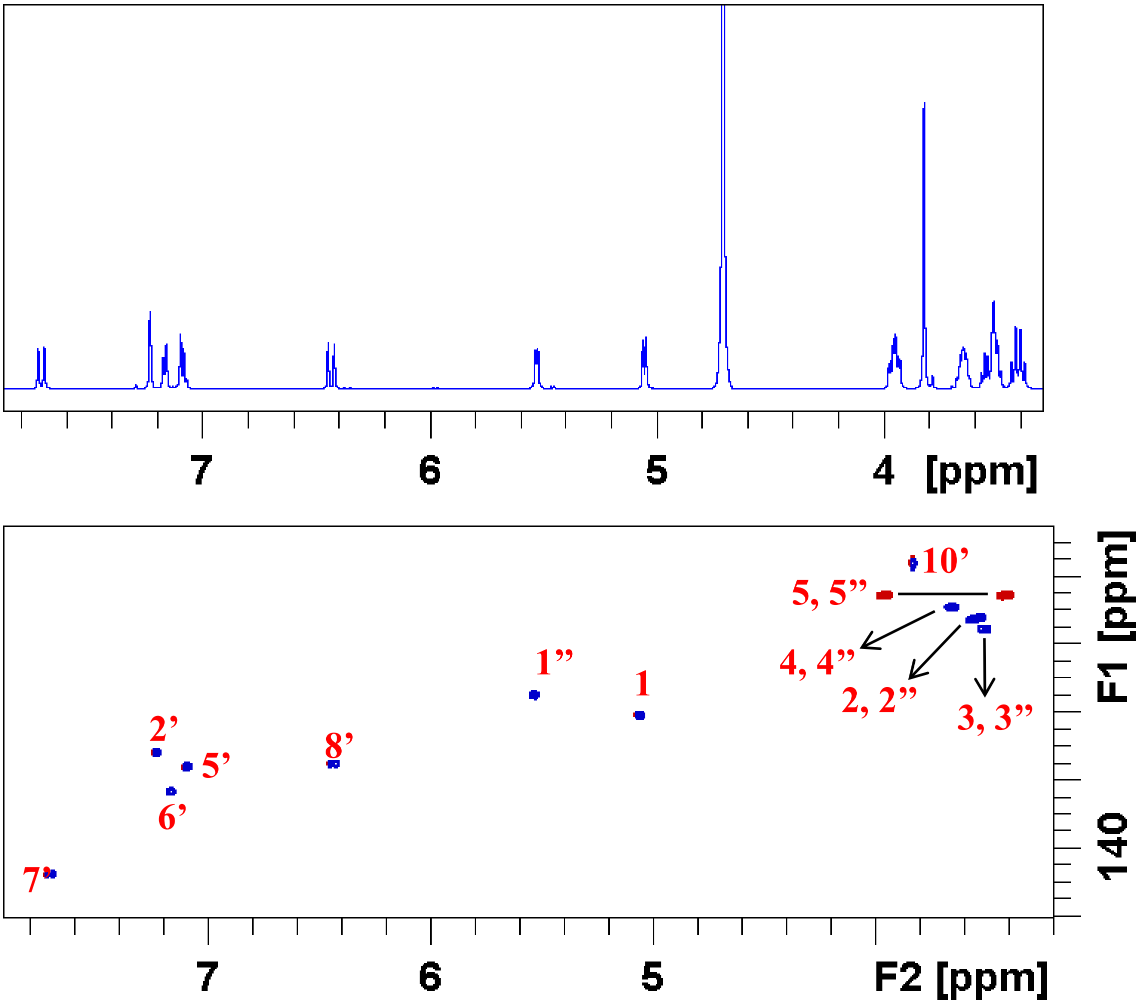

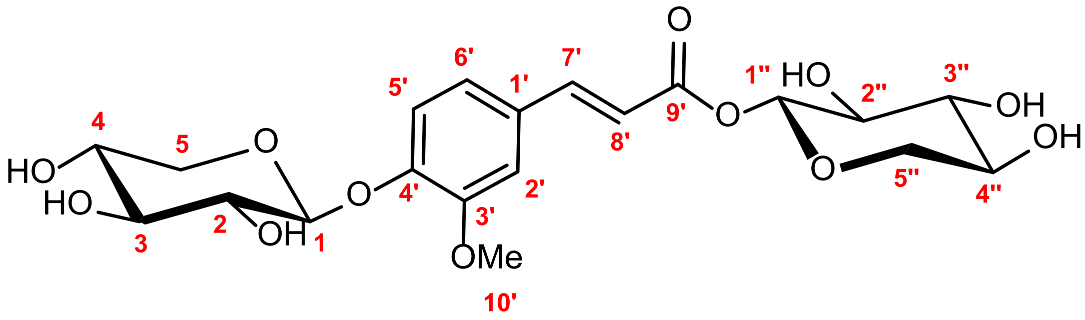

	^{1}H Chemical shift	^{13}C Chemical shift
**1**	5.05	100.65
**2**	3.56	72.65
**3**	3.50	75.33
**4**	3.65	68.96
**5**	3.40	65.55
**5**	3.95	
**1’**	---	129.38
**2’**	7.23	111.72
**3’**	---	148.97
**4’**	---	147.58
**5’**	7.09	115.84
**6’**	7.16	123.13
**7’**	7.71	147.46
**8’**	6.43	114.86
**9’**	---	167.42
**10’**	3.82	55.94

**1”**	5.53	94.81
**2”**	3.52	71.97
**3”**	3.50	75.33
**4”**	3.65	69.03
**5”**	3.40	65.35
**5”**	3.95	

Supplementary Figure 4. NMR assignment tables and spectra. Results are displayed for the identified glycoconjugates 2 (a), 4 (b), 6 (c), 8 (d), 10 (e), 11 (f), 13 (g), 15 (h), 17 (i), 19 (j), 21 (k), 23 (l), 25 (m), 27 (n), 29 (o), 31 (p), 33 (q), 35 (r), 36 (s), 37 (t), 39 (u), 40 (v) and 41 (w).

a)

Acceptor: *N*-Acetyl-L-glutamic acid

Mass Spectrum List Report

Acquisition Parameter

Ion Source Type	ESI	Ion Polarity	Negative	Alternating Ion Polarity	off
Mass Range Mode	Std/Enhanced	Scan Begin	100 m/z	Scan End	1200 m/z
Capillary Exit	-5.0 Volt	Skimmer	-40.0 Volt	Trap Drive	35.0
Accumulation Time	38826 µs	Averages	7 Spectra	Auto MS/MS	off

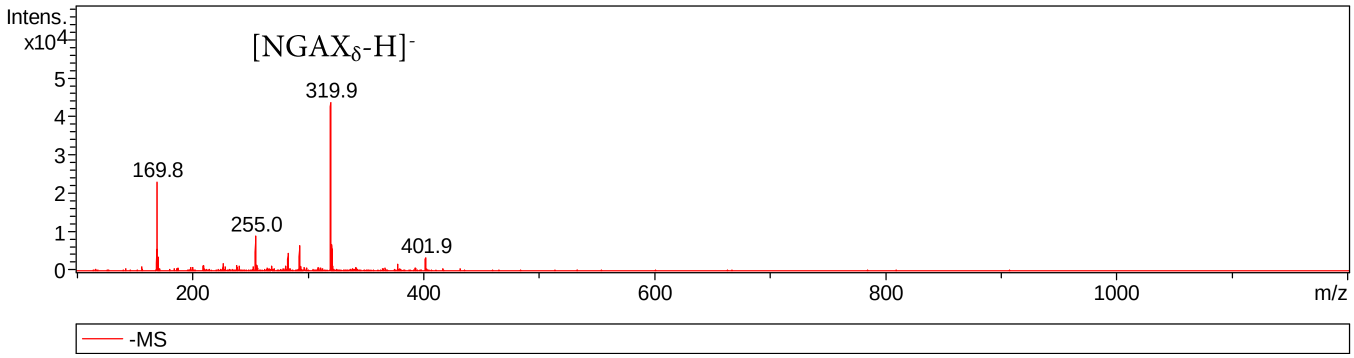

#	m/z	I
1	142.9	533
2	156.9	996
3	169.8	22994
4	170.8	3612
5	171.8	483
6	184.8	624
7	186.8	489
8	187.8	805
9	199.0	897
10	200.8	908
11	209.8	1412
12	210.8	533
13	227.0	1854
14	228.8	1035
15	238.8	1434
16	241.0	1251
17	253.0	1014
18	255.0	9022
19	256.0	1534
20	256.8	747
21	264.9	750
22	266.9	524
23	269.0	1219
24	271.0	502
25	279.0	611
26	281.0	1274
27	283.1	4626
28	284.0	614
29	285.0	493
30	293.0	6535
31	294.0	1158
32	297.0	870
33	299.0	728
34	308.9	814
35	311.0	783
36	313.0	562
37	319.9	43604
38	320.9	6840
39	321.9	1290
40	338.9	487
41	341.8	867
42	365.0	619
43	367.0	803
44	377.9	1664
45	379.8	533
46	393.0	790
47	401.9	3307
48	402.9	570
49	417.1	484
50	431.9	565

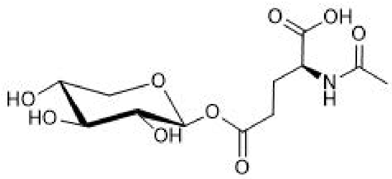

b)

Acceptor: *trans*-Cinnamic acid

Mass Spectrum List Report

Acquisition Parameter

Ion Source Type	ESI	Ion Polarity	Positive	Alternating Ion Polarity	off
Mass Range Mode	Std/Enhanced	Scan Begin	100 m/z	Scan End	1200 m/z
Capillary Exit	2.0 Volt	Skimmer	40.0 Volt	Trap Drive	27.0
Accumulation Time	12707 µs	Averages	9 Spectra	Auto MS/MS	off

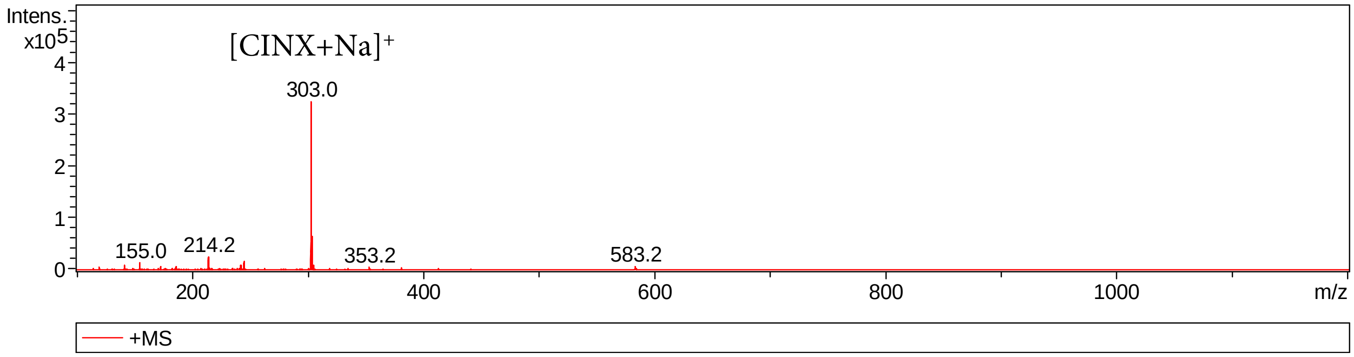

#	m/z	I
1	115.0	2829
2	120.0	4972
3	131.0	1984
4	133.0	1865
5	141.9	8846
6	144.0	1685
7	149.1	2661
8	155.0	14175
9	159.0	1839
10	171.0	3108
11	173.0	6335
12	177.0	2745
13	177.9	1928
14	182.9	2699
15	185.0	1846
16	186.2	6544
17	187.0	1876
18	208.0	3155
19	211.1	1849
20	214.2	25333
21	215.2	4203
22	217.0	2642
23	223.0	1892
24	224.0	3449
25	225.0	2002
26	227.0	2217
27	228.0	1681
28	235.1	3097
29	239.1	2765
30	241.1	2399
31	242.2	8836
32	244.0	2020
33	245.0	16553
34	246.0	2210
35	257.1	2049
36	263.0	2619

#	m/z	I
37	281.0	1956
38	294.0	1972
39	301.1	2332
40	303.0	324398
41	304.1	65137
42	305.1	8722
43	319.0	3394
44	332.4	1884
45	335.0	3317
46	353.2	5122
47	381.3	4430
48	413.2	2744
49	583.2	6280
50	584.1	2408

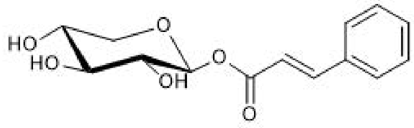

c)

Acceptor: Hexanoic acid

Mass Spectrum List Report

Acquisition Parameter

Ion Source Type	ESI	Ion Polarity	Positive	Alternating Ion Polarity	off
Mass Range Mode	Std/Enhanced	Scan Begin	100 m/z	Scan End	1200 m/z
Capillary Exit	5.0 Volt	Skimmer	40.0 Volt	Trap Drive	35.0
Accumulation Time	3655 µs	Averages	7 Spectra	Auto MS/MS	off

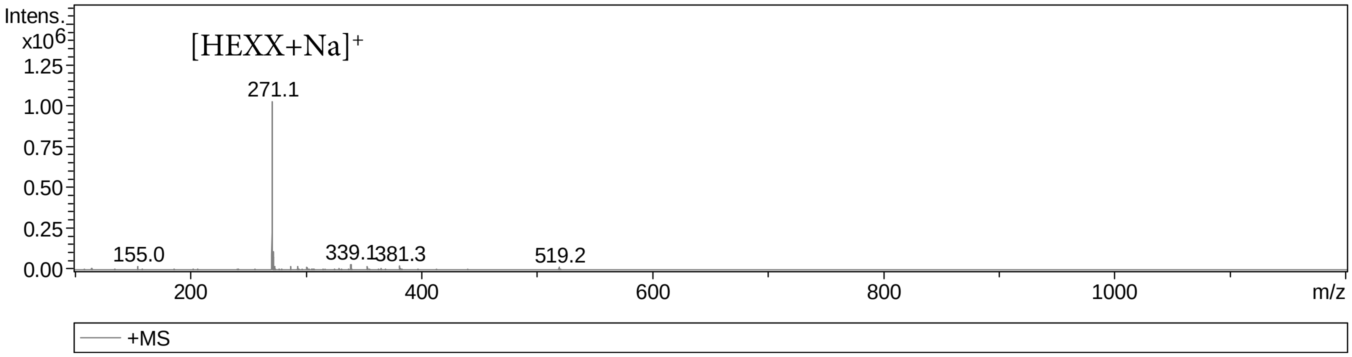

#	m/z	I
1	109.0	3774
2	115.1	9897
3	135.0	4337
4	155.0	19349
5	158.9	4617
6	186.2	5120
7	189.1	3312
8	203.0	4273
9	207.0	3501
10	241.1	6558
11	242.1	6180
12	256.2	3478
13	271.1	1024914
14	272.1	109930
15	273.1	22816
16	274.0	4602
17	277.1	3940
18	279.2	3578
19	287.0	20954
20	293.1	21316
21	293.9	4164
22	294.1	4935
23	297.2	4282
24	301.1	16224
25	302.2	5266
26	303.1	5743
27	305.1	4235
28	306.1	3874
29	307.3	3632
30	315.1	3487
31	317.0	6221
32	325.2	5087
33	329.1	7530
34	331.1	3421
35	337.2	5938
36	339.1	33862
37	340.0	4027
38	353.3	22847
39	355.2	5979
40	363.2	4516
41	365.1	10451
42	369.1	4643
43	381.3	24010
44	382.3	4646
45	383.2	5478
46	397.1	3647
47	413.3	5005
48	440.3	5967
49	519.2	18639
50	520.3	7069

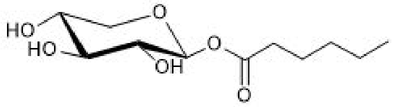

d)

Acceptor: Heptanoic acid

Mass Spectrum List Report

Acquisition Parameter

Ion Source Type	ESI	Ion Polarity	Positive	Alternating Ion Polarity	off
Mass Range Mode	Ultra Scan	Scan Begin	100 m/z	Scan End	1200 m/z
Capillary Exit	5.0 Volt	Skimmer	40.0 Volt	Trap Drive	33.0
Accumulation Time	3549 µs	Averages	14 Spectra	Auto MS/MS	off

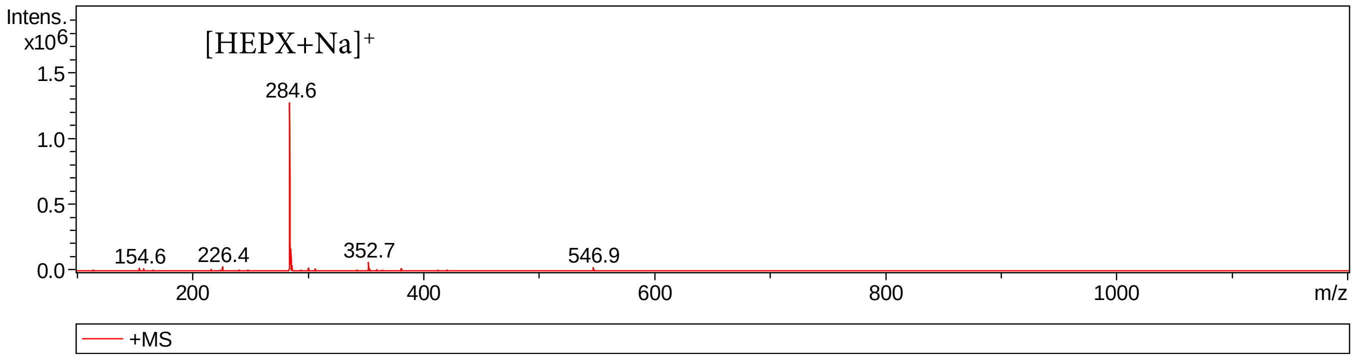

#	m/z	I
1	114.7	5553
2	132.6	3471
3	152.6	2389
4	154.6	22225
5	158.5	16804
6	166.5	4210
7	182.6	2670
8	184.6	2820
9	202.6	2667
10	216.5	9325
11	222.7	2355
12	224.6	4800
13	226.4	30930
14	237.6	2519
15	238.6	3419
16	240.6	6301
17	248.6	5009
18	270.6	3532
19	278.7	2332
20	282.6	2444
21	284.6	1274257
22	285.6	164459
23	286.6	38525
24	290.7	2270
25	294.5	6135
26	300.6	20541
27	301.7	3743
28	302.6	2902
29	304.7	2978
30	306.7	17647
31	308.7	2717
32	320.7	2654
33	336.7	3666
34	342.6	4907
35	344.7	2957
36	352.7	63646

#	m/z	I
37	353.7	10923
38	354.6	2557
39	359.9	9653
40	362.5	2792
41	364.8	4378
42	367.9	2465
43	380.9	18624
44	381.9	2622
45	408.8	2342
46	412.9	6333
47	420.7	7085
48	546.9	25622
49	547.9	5244
50	596.0	2696

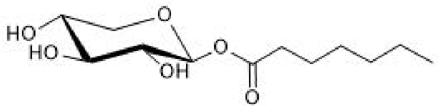

e) **Acceptor: Vanillin**

Mass Spectrum List Report

Acquisition Parameter

Ion Source Type	ESI	Ion Polarity	Positive	Alternating Ion Polarity	off
Mass Range Mode	Std/Enhanced	Scan Begin	100 m/z	Scan End	1200 m/z
Capillary Exit	5.0 Volt	Skimmer	40.0 Volt	Trap Drive	35.0
Accumulation Time	4005 µs	Averages	7 Spectra	Auto MS/MS	off

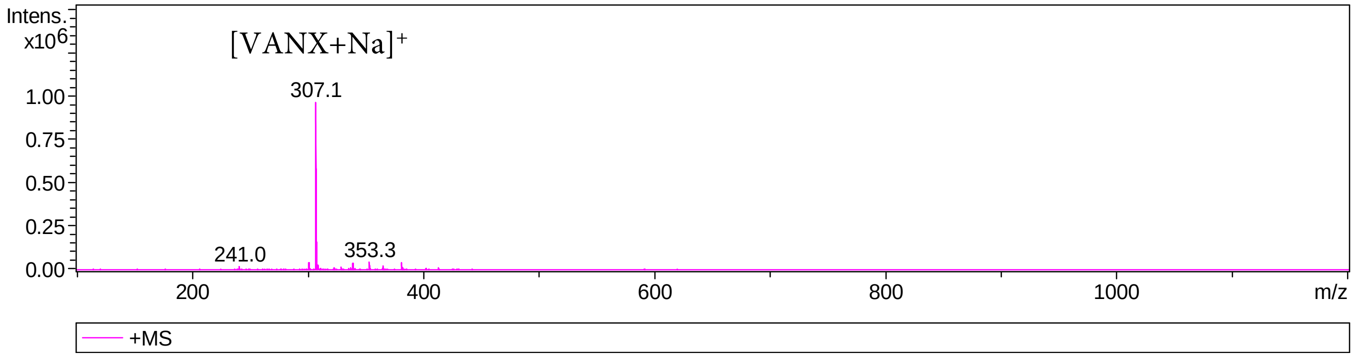

#	m/z	I
1	115.1	4550
2	206.9	4750
3	225.1	5079
4	237.1	4504
5	241.0	20215
6	257.0	4362
7	266.9	5911
8	269.1	5479
9	277.0	5933
10	279.2	6401
11	281.2	5176
12	293.2	4752
13	298.9	6862
14	299.2	5356
15	301.2	41920
16	302.1	8774
17	305.2	5932
18	307.1	962895
19	308.1	158812
20	309.0	26757
21	311.1	10320
22	313.2	5091
23	317.1	5880
24	322.2	5450
25	323.0	12253
26	325.2	5256
27	329.1	17296
28	330.1	5117
29	335.3	7266
30	337.2	13345
31	339.1	38486
32	339.9	6838
33	341.2	7211
34	345.1	4433
35	353.3	45561
36	354.2	7173

#	m/z	I
37	365.1	22658
38	366.1	6844
39	381.3	42383
40	382.3	10887
41	383.2	4644
42	385.3	4630
43	402.4	10214
44	413.3	12115
45	430.4	5652
46	442.4	5379
47	591.1	5100
48	591.4	4867
49	619.4	5233
50	619.6	6014

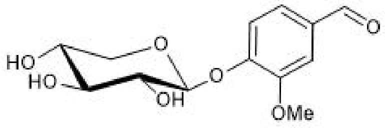

f)

Donor: *p*NPAp   Acceptor: Vanillin

Mass Spectrum List Report

Acquisition Parameter

Ion Source Type	ESI	Ion Polarity	Positive	Alternating Ion Polarity	off
Mass Range Mode	Std/Enhanced	Scan Begin	50 m/z	Scan End	1200 m/z
Capillary Exit	5.0 Volt	Skimmer	40.0 Volt	Trap Drive	30.0
Accumulation Time	4602 µs	Averages	8 Spectra	Auto MS/MS	off

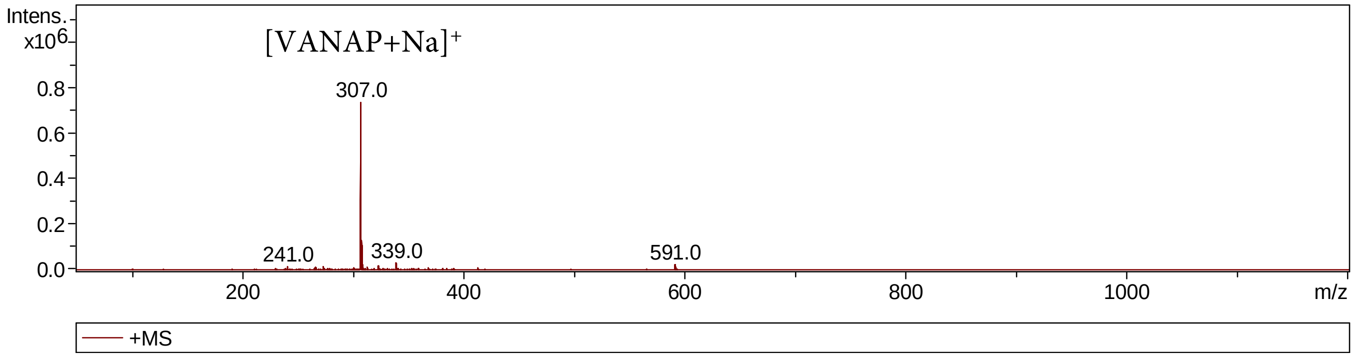

#	m/z	I
1	100.9	3055
2	129.0	5293
3	191.0	2975
4	211.0	3281
5	213.0	3996
6	225.0	2444
7	230.1	7299
8	231.1	2581
9	238.0	2759
10	239.0	5703
11	241.0	15291
12	243.0	3156
13	245.0	3615
14	248.9	2494
15	251.0	3914
16	252.0	3921
17	252.8	3520
18	253.0	3272
19	255.0	3767
20	261.0	2866
21	265.0	5493
22	266.1	11666
23	267.0	9003
24	269.0	5161
25	271.0	2774
26	273.1	14448
27	274.1	7423
28	277.2	7259
29	278.1	3492
30	279.1	5879
31	280.1	2954
32	281.1	2912
33	284.2	4349
34	286.9	3223
35	289.1	2604
36	290.1	4358

#	m/z	I
37	291.1	4771
38	293.1	4835
39	294.1	3374
40	295.1	4293
41	297.1	3101
42	299.1	3418
43	300.2	2854
44	301.0	8725
45	301.8	4251
46	303.1	2878
47	304.1	3904
48	305.1	4251
49	307.0	735697
50	308.0	131864
51	309.0	24917
52	310.1	3416
53	311.1	6543
54	313.1	11111
55	314.1	2443
56	317.0	3831
57	319.2	5700
58	323.0	17969
59	324.0	6401
60	325.1	5000
61	327.1	5813
62	328.3	3109
63	328.8	5116
64	329.2	2671
65	331.1	6422
66	333.0	2929
67	335.2	4871
68	337.1	4450
69	339.0	32975
70	339.9	5539
71	340.5	2503
72	341.3	8008

#	m/z	I
73	343.2	2752
74	347.0	3776
75	349.0	2525
76	350.1	2475
77	351.2	2990
78	353.1	5878
79	354.2	4161
80	355.2	6140
81	357.2	5151
82	359.2	7049
83	365.2	4237
84	368.3	9180
85	369.4	3212
86	372.1	3378
87	374.2	3021
88	375.0	5249
89	381.2	5863
90	385.0	5421
91	389.2	2940
92	391.0	5610
93	392.0	2490
94	413.2	8187
95	419.2	2849
96	497.2	2579
97	565.2	2770
98	591.0	22643
99	592.0	12559
100	593.1	3106

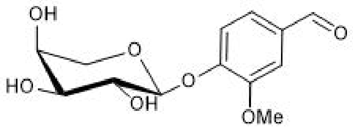

g)

Acceptor: Epigallocatechin gallate

Mass Spectrum List Report

Acquisition Parameter

Ion Source Type	ESI	Ion Polarity	Positive	Alternating Ion Polarity	off
Mass Range Mode	Std/Enhanced	Scan Begin	100 m/z	Scan End	1200 m/z
Capillary Exit	95.4 Volt	Skimmer	40.0 Volt	Trap Drive	69.7
Accumulation Time	1000 µs	Averages	7 Spectra	Auto MS/MS	off

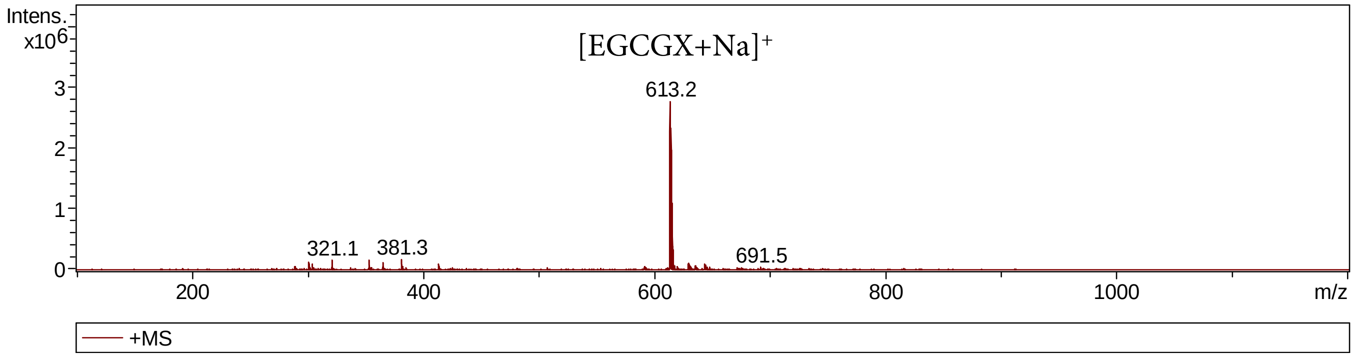

#	m/z	I
1	192.1	28434
2	273.1	26146
3	289.0	61004
4	301.1	134397
5	302.1	46576
6	304.2	97733
7	305.2	31416
8	321.1	161533
9	322.1	29657
10	337.1	32259
11	353.3	158377
12	354.3	27588
13	355.0	34814
14	365.1	116121
15	366.1	26220
16	381.3	170833
17	382.3	52392
18	385.2	36409
19	413.3	98932
20	425.2	33917
21	481.2	27087
22	507.3	31419
23	591.2	58845
24	591.6	39383
25	592.2	40268
26	611.2	35920
27	612.2	25490
28	613.2	2766370
29	614.2	2326536
30	615.2	1094340
31	616.2	328161
32	617.2	62393
33	619.6	61757
34	629.1	113192
35	630.1	89414
36	631.2	58100
37	632.2	28506
38	635.2	71590
39	636.2	45898
40	643.2	96161
41	644.1	73187
42	645.2	50646
43	647.6	43771
44	671.1	31841
45	672.1	25332
46	675.2	31470
47	676.2	29717
48	691.5	44890
49	713.2	27390
50	719.6	28933

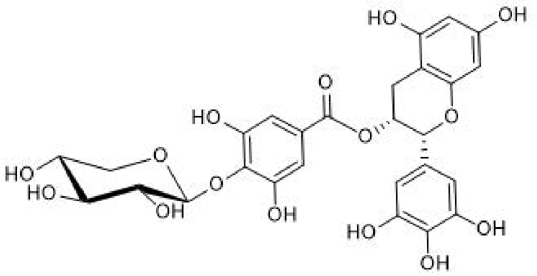

h)

Acceptor: *N*-Hydroxybenzotriazole

Mass Spectrum List Report

Acquisition Parameter

Ion Source Type	ESI	Ion Polarity	Positive	Alternating Ion Polarity	off
Mass Range Mode	Std/Enhanced	Scan Begin	100 m/z	Scan End	1200 m/z
Capillary Exit	5.0 Volt	Skimmer	40.0 Volt	Trap Drive	35.0
Accumulation Time	4330 µs	Averages	7 Spectra	Auto MS/MS	off

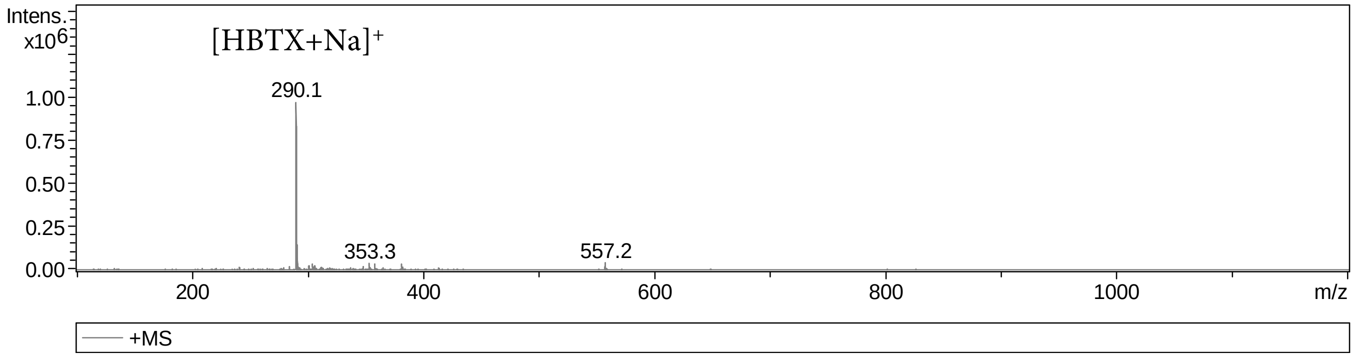

#	m/z	I
1	132.9	7850
2	177.0	6434
3	208.9	8242
4	221.0	8255
5	235.0	6481
6	237.0	6523
7	241.1	14955
8	253.1	7854
9	265.1	7601
10	269.1	6981
11	277.2	8452
12	278.2	6468
13	279.3	13614
14	284.4	20805
15	290.1	969829
16	291.1	147217
17	292.1	15990
18	293.2	12602
19	297.2	9832
20	301.1	24930
21	304.2	35411
22	305.3	15567
23	306.1	25532
24	307.0	8512
25	307.2	9453
26	311.2	13105
27	312.1	16170
28	313.2	8521
29	316.1	6963
30	317.2	10006
31	318.1	6938
32	319.2	11945
33	320.2	6708
34	321.2	9037
35	337.1	11249
36	339.2	7253
37	348.0	20077
38	353.3	38764
39	354.3	12503
40	358.0	34015
41	359.1	7557
42	360.3	6763
43	365.2	11952
44	381.3	34036
45	382.3	14952
46	389.4	6436
47	413.3	13969
48	557.2	42607
49	558.1	9662
50	648.4	6419

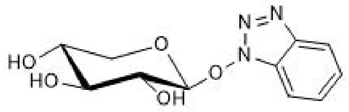

i)

Acceptor: 5-(5-Bromo-2-thienyl)-1*H*-tetrazole

Mass Spectrum List Report

Acquisition Parameter

Ion Source Type	ESI	Ion Polarity	Positive	Alternating Ion Polarity	off
Mass Range Mode	Std/Enhanced	Scan Begin	50 m/z	Scan End	1200 m/z
Capillary Exit	3.0 Volt	Skimmer	40.0 Volt	Trap Drive	35.0
Accumulation Time	5060 µs	Averages	6 Spectra	Auto MS/MS	off

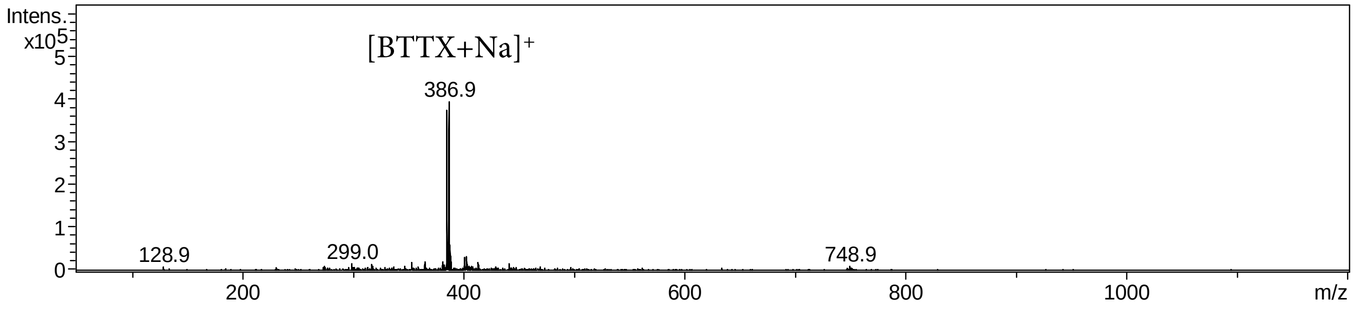

#	m/z	I
1	128.9	8397
2	134.0	4275
3	230.8	6874
4	248.0	3895
5	273.1	6323
6	274.2	10252
7	275.0	6035
8	277.0	4715
9	279.1	5268
10	285.0	3870
11	290.9	4146
12	296.1	6199
13	299.0	16148
14	300.0	7121
15	301.1	6428
16	304.0	5783
17	305.2	5407
18	311.2	5473
19	313.3	6850
20	317.1	14472
21	318.1	9938
22	321.1	4584
23	325.1	4426
24	329.1	7232
25	333.2	4412
26	335.1	5789
27	337.1	7958
28	340.9	4257
29	343.0	4204
30	347.0	9560
31	353.2	19035
32	354.2	5792
33	355.2	4568
34	357.3	5722
35	359.1	8784
36	365.1	20548
37	366.2	4738
38	367.0	3807
39	369.2	4483
40	373.2	3766
41	374.1	3883
42	375.0	4244
43	377.4	5241
44	379.2	4829
45	381.2	20350
46	382.3	12374
47	383.1	6085
48	384.2	3792
49	384.9	373704
50	385.9	65627
51	386.9	393437
52	387.9	59267
53	388.8	19861
54	391.1	5015
55	392.3	4360
56	399.2	4671
57	400.9	30338
58	401.9	8685
59	402.2	4681
60	402.8	31776
61	403.9	7107
62	404.7	9924
63	405.1	6807
64	406.2	5873
65	406.8	4793
66	407.2	9980
67	407.9	4767
68	408.2	7670
69	411.2	5293
70	413.2	18563
71	414.1	4273
73	421.3	4226
74	425.2	4473
75	428.4	4588
76	429.2	7403
77	430.3	5603
78	431.4	5304
79	435.3	4843
80	441.2	14982
81	442.2	4428
82	443.3	5333
83	445.3	6791
84	447.4	6804
85	455.2	4440
86	459.2	3918
87	463.4	3677
88	465.2	4605
89	466.9	4033
90	469.3	8142
91	473.3	5288
92	484.9	5705
93	497.1	6179
94	511.2	3940
95	561.5	4801
96	633.5	4781
97	746.9	4763
98	748.9	9360
99	749.9	5349
100	750.9	4890

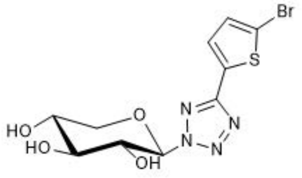

j)

Acceptor: Hydrazoic acid

Mass Spectrum List Report

Acquisition Parameter

Ion Source Type	ESI	Ion Polarity	Positive	Alternating Ion Polarity	off
Mass Range Mode	Std/Enhanced	Scan Begin	100 m/z	Scan End	1200 m/z
Capillary Exit	5.0 Volt	Skimmer	40.0 Volt	Trap Drive	27.0
Accumulation Time	12825 µs	Averages	12 Spectra	Auto MS/MS	off

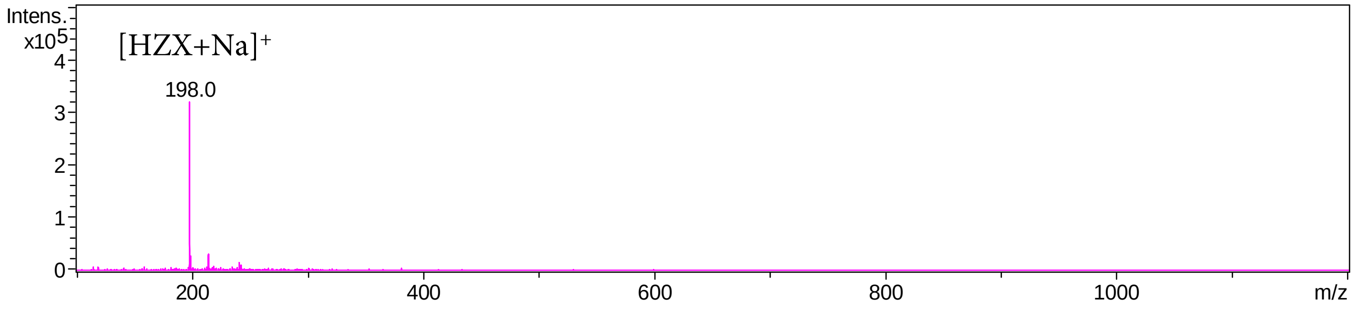

#	m/z	I
1	115.0	5977
2	118.9	5955
3	127.0	2720
4	141.0	4311
5	149.9	3067
6	157.0	2683
7	158.9	6398
8	160.9	2845
9	173.0	3359
10	174.9	2948
11	177.0	3997
12	181.9	4882
13	185.0	2876
14	186.2	4348
15	186.9	2951
16	196.9	4891
17	198.0	320605
18	199.0	27759
19	199.9	2795
20	200.9	4891
21	203.0	3165
22	205.0	2600
23	209.0	3299
24	211.1	3508
25	213.1	6449
26	214.2	30869
27	215.1	4554
28	216.9	2646
29	217.8	4877
30	218.9	7928
31	219.9	3460
32	220.9	3892
33	225.0	4930
34	227.0	2754
35	234.9	6041
36	235.9	3449
37	236.8	2999
38	239.1	5862
39	241.0	14729
40	242.2	10350
41	245.1	3103
42	249.9	3003
43	263.0	2581
44	266.1	3600
45	269.2	3042
46	277.1	2893
47	279.2	3469
48	301.1	3752
49	304.3	3004
50	381.3	4646

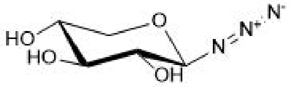

k)

Acceptor: 3,5-Dibromo-1,2,4-triazole

Mass Spectrum List Report

Acquisition Parameter

Ion Source Type	ESI	Ion Polarity	Positive	Alternating Ion Polarity	off
Mass Range Mode	Std/Enhanced	Scan Begin	50 m/z	Scan End	1200 m/z
Capillary Exit	5.0 Volt	Skimmer	40.0 Volt	Trap Drive	30.0
Accumulation Time	11678 µs	Averages	8 Spectra	Auto MS/MS	off

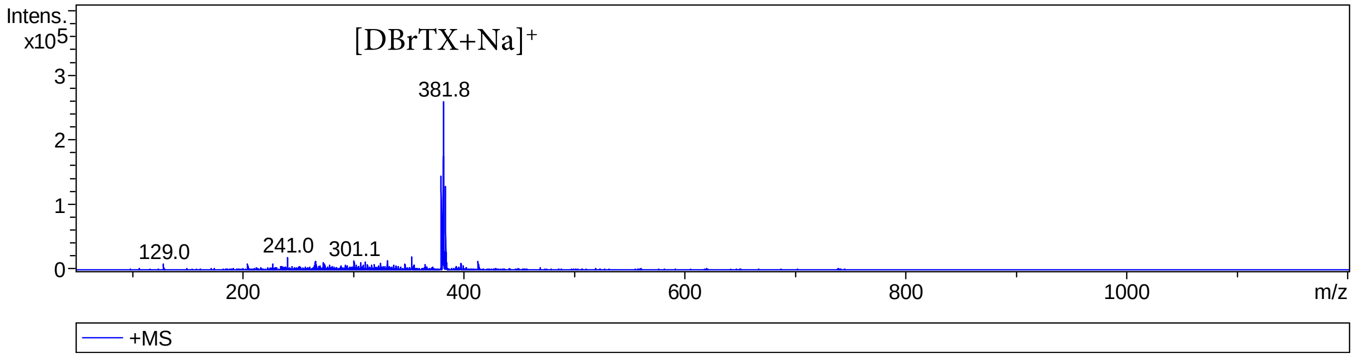

#	m/z	I	#	m/z	I	#	m/z	I
1	129.0	8773	37	289.1	5875	73	339.1	6661
2	205.0	9426	38	291.1	3725	74	341.2	5117
3	217.0	3252	39	293.1	6894	75	343.1	4168
4	223.1	3496	40	294.1	3187	76	347.1	9383
5	225.8	3280	41	295.1	6534	77	351.1	3330
6	227.8	9163	42	297.1	3906	78	353.2	19861
7	229.8	3047	43	299.1	4342	79	354.2	4713
8	235.0	5410	44	300.1	4002	80	355.2	7204
9	236.0	5282	45	301.1	14625	81	365.2	8014
10	236.9	3924	46	302.1	3635	82	366.1	4013
11	238.0	3639	47	302.3	3685	83	367.2	3994
12	239.0	4629	48	303.1	7671	84	371.2	3174
13	241.0	18695	49	304.0	3266	85	372.3	4653
14	245.0	4971	50	305.2	5423	86	379.8	144350
15	249.0	3155	51	307.1	11565	87	380.8	19875
16	251.1	4187	52	308.2	4085	88	381.3	18475
17	252.1	5169	53	309.2	7177	89	381.8	259341
18	253.0	3351	54	311.1	12078	90	382.8	28275
19	255.0	3429	55	313.2	8532	91	383.8	128472
20	257.1	4209	56	314.1	3662	92	384.8	11450
21	259.1	3184	57	315.1	3965	93	393.2	5449
22	261.0	4408	58	317.1	7422	94	394.2	3127
23	265.0	6384	59	319.1	7815	95	395.7	4610
24	266.1	12843	60	320.1	3086	96	397.8	9802
25	267.1	8039	61	321.1	4511	97	399.8	5971
26	269.1	5291	62	323.1	6152	98	402.3	3529
27	270.2	6045	63	325.2	10059	99	413.2	13368
28	271.1	3678	64	326.2	3080	100	414.2	4151
29	273.1	11402	65	327.2	4975			
30	274.2	8834	66	329.1	5231			
31	275.1	5879	67	331.2	14605			
32	277.0	4193	68	332.1	3622			
33	279.1	7252	69	333.1	5172			
34	280.1	3418	70	334.2	3227			
35	281.1	4804	71	335.2	3907			
36	283.1	4586	72	337.1	7023			

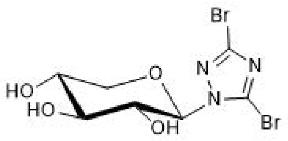

1)

Acceptor: 1*H*-1,2,3-Triazolo[4,5-b]pyridine

Mass Spectrum List Report

Acquisition Parameter

Ion Source Type	ESI	Ion Polarity	Positive	Alternating Ion Polarity	off
Mass Range Mode	Std/Enhanced	Scan Begin	50 m/z	Scan End	1200 m/z
Capillary Exit	3.0 Volt	Skimmer	40.0 Volt	Trap Drive	25.0
Accumulation Time	5467 µs	Averages	6 Spectra	Auto MS/MS	off

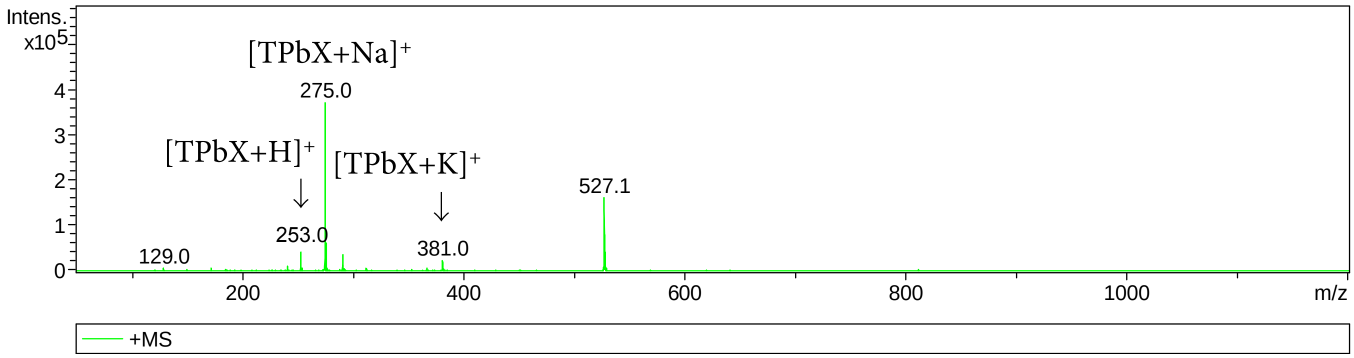

#	m/z	I
1	129.0	6152
2	171.9	6117
3	241.0	9812
4	253.0	41632
5	254.0	6576
6	273.0	3543
7	275.0	371379
8	276.0	60929
9	276.8	6004
10	288.1	3883
11	288.3	3558
12	291.0	35360
13	291.9	5446
14	312.0	5478
15	367.1	6719
16	381.0	23650
17	381.9	4089
18	382.1	5291
19	526.4	5137
20	527.1	161078
21	527.7	6766
22	528.1	40955
23	528.8	4989
24	529.1	6091

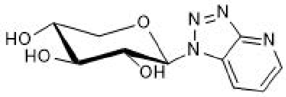

m)

Acceptor: Phenylphosphonic acid

Mass Spectrum List Report

Acquisition Parameter

Ion Source Type	ESI	Ion Polarity	Negative	Alternating Ion Polarity	off
Mass Range Mode	Std/Enhanced	Scan Begin	50 m/z	Scan End	1200 m/z
Capillary Exit	-3.0 Volt	Skimmer	-40.0 Volt	Trap Drive	30.0
Accumulation Time	9238 µs	Averages	6 Spectra	Auto MS/MS	off

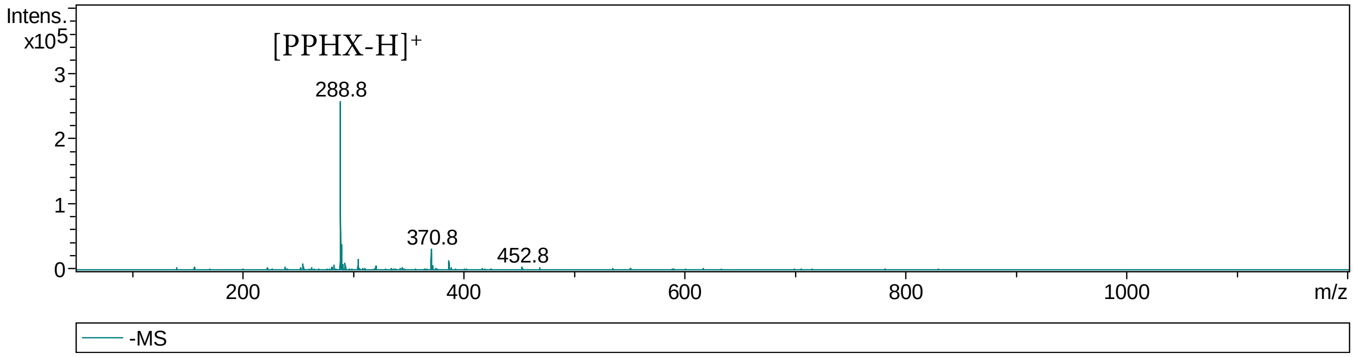

#	m/z	I
1	140.8	2894
2	156.8	3911
3	222.8	3742
4	238.8	4512
5	252.8	2993
6	255.0	9630
7	262.8	3020
8	281.0	4220
9	283.0	7454
10	288.8	256723
11	289.8	38622
12	290.8	8161
13	292.9	9743
14	304.8	15685
15	309.0	2564
16	320.8	5882
17	344.8	2816
18	370.8	32016
19	371.8	6609
20	374.9	2519
21	386.8	13856
22	389.0	3154
23	417.1	2662
24	452.8	4490
25	468.8	3331
26	550.8	2207
27	616.8	2203

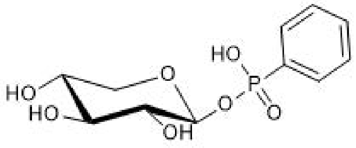

n)

Acceptor: Phosphoric acid

Mass Spectrum List Report

Acquisition Parameter

Ion Source Type	ESI	Ion Polarity	Negative	Alternating Ion Polarity	off
Mass Range Mode	Std/Enhanced	Scan Begin	100 m/z	Scan End	1200 m/z
Capillary Exit	-5.0 Volt	Skimmer	-40.0 Volt	Trap Drive	25.0
Accumulation Time	139370 µs	Averages	12 Spectra	Auto MS/MS	off

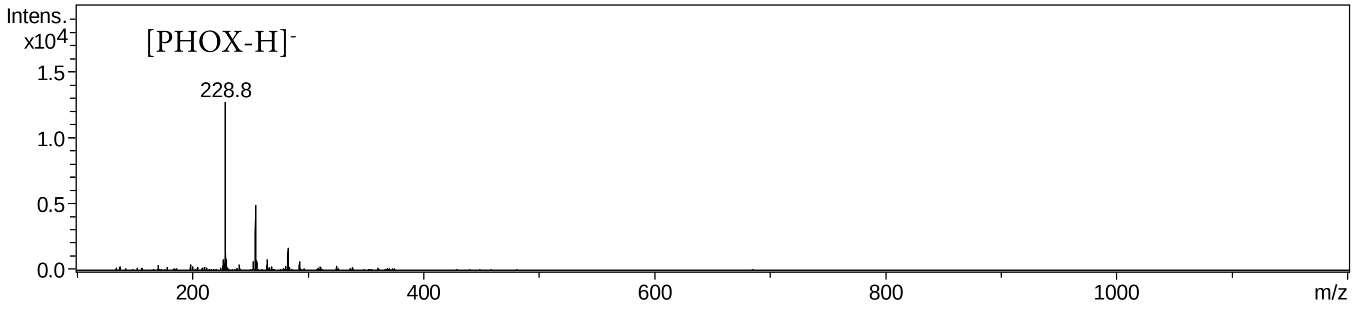

#	m/z	I
1	134.9	140
2	137.8	269
3	142.9	123
4	152.8	165
5	156.9	176
6	170.9	337
7	178.7	222
8	184.8	109
9	186.8	130
10	198.9	408
11	200.7	257
12	204.9	205
13	208.7	144
14	210.9	197
15	212.9	147
16	224.9	170
17	227.0	784
18	228.0	181
19	228.8	12692
20	229.8	780
21	230.8	183
22	238.7	137
23	238.9	108
24	241.0	400
25	253.0	627
26	255.0	4908
27	256.0	747
28	264.9	801
29	265.8	129
30	267.0	209
31	269.0	272
32	279.0	121
33	281.0	314
34	283.0	1689
35	284.1	227
36	292.9	656

#	m/z	I
37	293.9	106
38	296.9	112
39	309.0	169
40	311.0	260
41	325.0	288
42	326.9	109
43	336.9	105
44	339.0	228
45	360.8	154
46	368.9	113
47	370.8	108
48	373.5	123
49	375.0	103

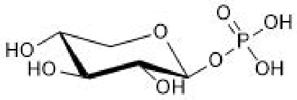

o)

Acceptor: Thiophenol

Mass Spectrum List Report

Acquisition Parameter

Ion Source Type	ESI	Ion Polarity	Negative	Alternating Ion Polarity	off
Mass Range Mode	Std/Enhanced	Scan Begin	100 m/z	Scan End	1200 m/z
Capillary Exit	-5.0 Volt	Skimmer	-40.0 Volt	Trap Drive	35.0
Accumulation Time	13201 µs	Averages	7 Spectra	Auto MS/MS	off

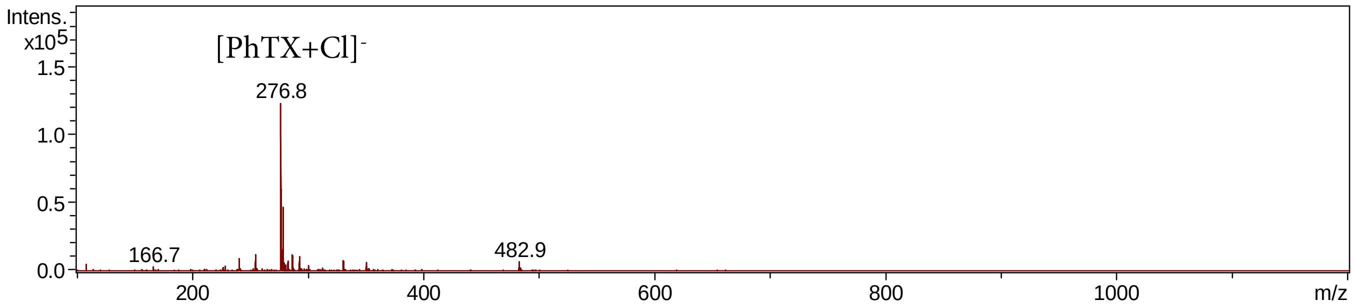

#	m/z	I
1	108.8	5110
2	115.0	1046
3	156.9	1037
4	166.7	2789
5	170.9	990
6	198.9	1340
7	210.9	1276
8	213.0	1164
9	226.9	2653
10	228.7	3644
11	239.0	940
12	240.8	9236
13	241.9	1582
14	252.9	1816
15	255.0	11960
16	256.0	1966
17	260.8	1387
18	265.0	1277
19	268.9	1321
20	276.8	123122
21	277.8	15051
22	278.8	46567
23	279.8	5922
24	280.9	4471
25	283.0	7166
26	284.0	1185
27	286.8	11970
28	287.8	1916
29	293.0	10307
30	294.0	1585
31	295.0	1577
32	297.0	1415
33	298.8	1282
34	300.8	4070
35	301.9	1042
36	308.9	1180

#	m/z	I
37	310.9	1000
38	312.9	1925
39	330.8	7728
40	331.8	1101
41	332.8	1336
42	350.8	6112
43	351.9	1298
44	352.9	1753
45	356.9	1100
46	360.8	1161
47	372.8	1053
48	399.0	989
49	482.9	6667
50	483.8	2413

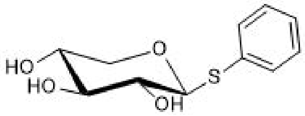

p)

Acceptor: Benzenesulfinic acid

Mass Spectrum List Report

Acquisition Parameter

Ion Source Type	ESI	Ion Polarity	Positive	Alternating Ion Polarity	off
Mass Range Mode	Ultra Scan	Scan Begin	100 m/z	Scan End	800 m/z
Capillary Exit	5.0 Volt	Skimmer	40.0 Volt	Trap Drive	30.0
Accumulation Time	2498 µs	Averages	11 Spectra	Auto MS/MS	off

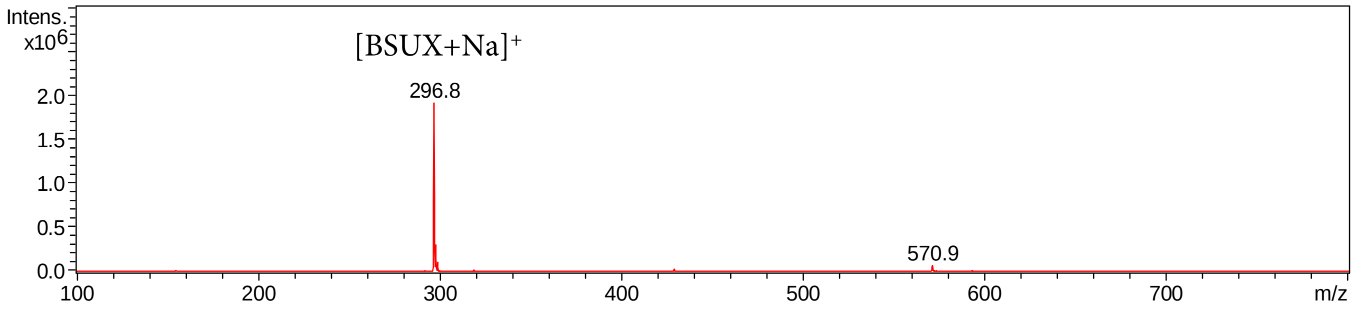

#	m/z	I
1	154.9	11208
2	164.9	3973
3	274.8	3752
4	291.8	7248
5	296.8	1917273
6	297.8	302842
7	298.8	105581
8	299.8	12979
9	300.9	6132
10	317.8	1961
11	318.8	17126
12	319.9	2343
13	320.8	2388
14	386.8	4043
15	428.9	25473
16	429.9	6208
17	430.9	3948
18	568.8	2103
19	570.9	70142
20	571.9	12572
21	573.0	10922
22	574.0	2381
23	593.0	13125
24	593.9	3786
25	615.2	2058

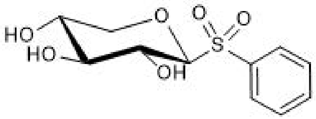

q)

Acceptor: Benzeneselenol

Mass Spectrum List Report

Acquisition Parameter

Ion Source Type	ESI	Ion Polarity	Positive	Alternating Ion Polarity	off
Mass Range Mode	Std/Enhanced	Scan Begin	100 m/z	Scan End	1000 m/z
Capillary Exit	5.0 Volt	Skimmer	40.0 Volt	Trap Drive	40.0
Accumulation Time	115000 µs	Averages	7 Spectra	Auto MS/MS	off

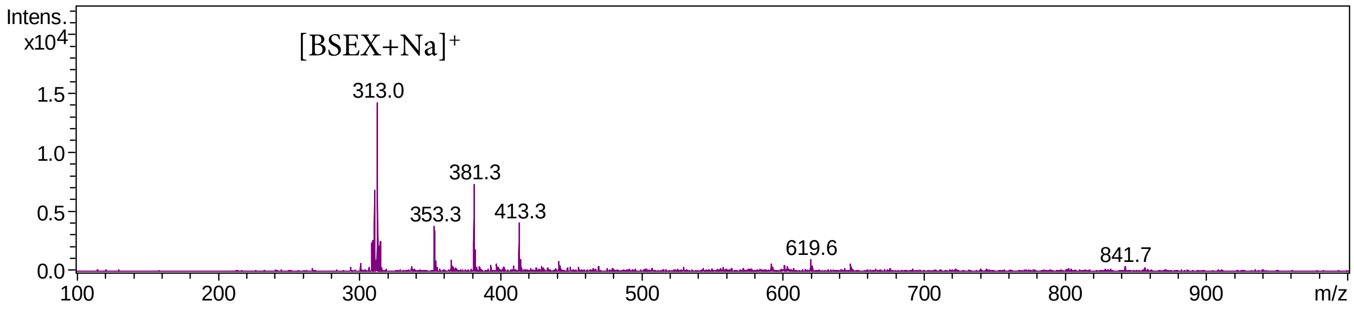

#	m/z	I
1	294.1	352
2	301.1	689
3	307.0	351
4	309.0	2393
5	310.0	2620
6	311.0	6836
7	312.0	939
8	313.0	14214
9	314.0	2146
10	315.0	2487
11	316.0	290
12	337.2	397
13	353.3	3783
14	354.3	870
15	355.3	327
16	365.2	953
17	366.2	409
18	368.4	289
19	381.3	7296
20	382.3	1821
21	383.3	369
22	385.2	408
23	393.3	493
24	397.2	589
25	398.3	350
26	402.3	342
27	409.3	447
28	413.3	4087
29	414.3	1014
30	425.3	306
31	429.3	387
32	430.4	309
33	433.4	282
34	441.3	816
35	442.3	425
36	447.4	296
37	449.4	321
38	455.3	323
39	469.4	393
40	529.4	333
41	557.5	362
42	591.6	597
43	592.5	364
44	600.9	483
45	603.0	408
46	619.6	981
47	620.6	446
48	647.6	627
49	841.7	410
50	855.7	275

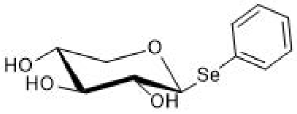

r)

Acceptor: *p*-Coumaric acid

Mass Spectrum List Report

Acquisition Parameter

Ion Source Type	ESI	Ion Polarity	Negative	Alternating Ion Polarity	off
Mass Range Mode	Std/Enhanced	Scan Begin	100 m/z	Scan End	1200 m/z
Capillary Exit	-5.0 Volt	Skimmer	-40.0 Volt	Trap Drive	35.0
Accumulation Time	10056 µs	Averages	7 Spectra	Auto MS/MS	off

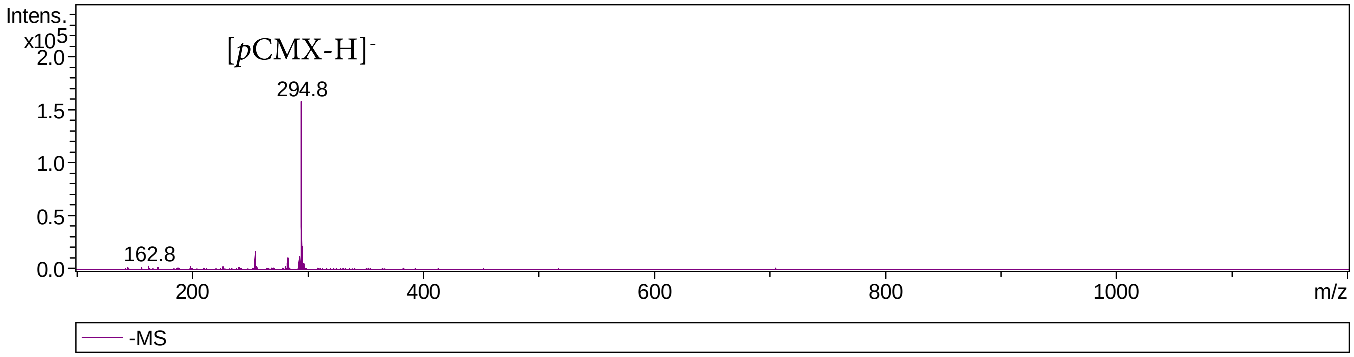

#	m/z	I
1	144.8	1815
2	156.8	2023
3	162.8	3330
4	170.9	1773
5	184.8	1076
6	186.8	914
7	187.8	1378
8	188.7	1256
9	198.9	2413
10	210.8	1707
11	212.9	920
12	224.7	794
13	226.9	2891
14	232.8	945
15	234.9	1080
16	241.0	2180
17	242.8	858
18	253.0	1695
19	255.0	16821
20	256.0	3008
21	264.9	1655
22	266.9	844
23	268.9	1441
24	270.8	1476
25	278.9	1338
26	281.0	2549
27	282.0	976
28	283.1	10795
29	284.0	1502
30	285.0	822
31	293.0	12382
32	293.9	2295
33	294.8	157734
34	295.8	21872
35	296.9	5662
36	297.9	1021

#	m/z	I
37	299.0	1051
38	309.0	1380
39	310.7	840
40	311.0	1011
41	316.9	944
42	330.8	955
43	340.9	886
44	352.9	1141
45	354.9	819
46	365.0	906
47	383.0	1255
48	393.1	1087
49	517.0	1096
50	704.8	1442

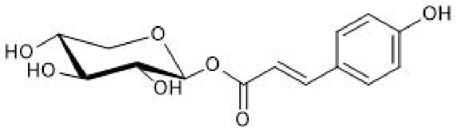

s)

Acceptor: *p*-Coumaric acid

Mass Spectrum List Report

Acquisition Parameter

Ion Source Type	ESI	Ion Polarity	Negative	Alternating Ion Polarity	off
Mass Range Mode	Std/Enhanced	Scan Begin	100 m/z	Scan End	1200 m/z
Capillary Exit	-5.0 Volt	Skimmer	-40.0 Volt	Trap Drive	35.0
Accumulation Time	25064 µs	Averages	7 Spectra	Auto MS/MS	off

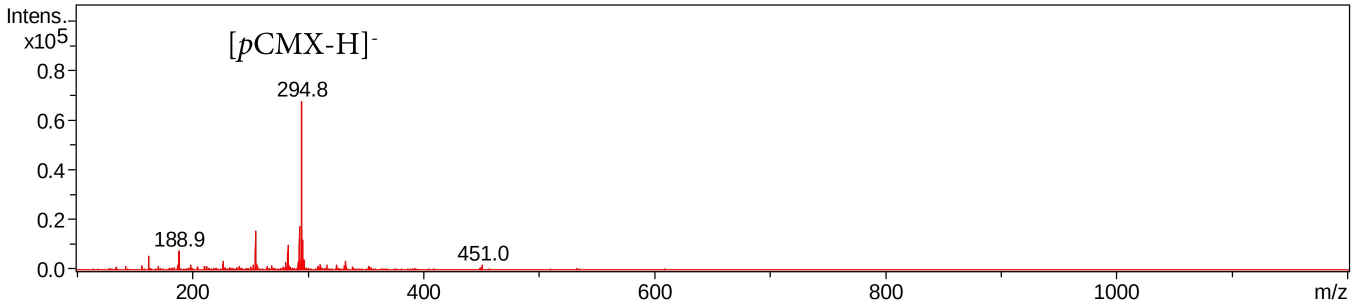

#	m/z	I
1	128.8	671
2	134.8	1032
3	142.8	1338
4	156.9	1529
5	162.8	5389
6	170.9	1391
7	184.9	778
8	187.8	1799
9	188.9	7508
10	198.9	1846
11	199.9	645
12	204.9	1014
13	210.9	1497
14	212.8	1489
15	220.7	643
16	226.9	3329
17	228.7	957
18	232.8	908
19	238.9	876
20	241.0	1454
21	250.9	1117
22	253.0	1843
23	255.0	15464
24	256.0	2282
25	256.8	1017
26	264.9	1446
27	269.0	1557
28	278.9	1122
29	281.1	2804
30	282.0	850
31	283.0	9751
32	284.1	1609
33	285.0	1153
34	291.9	3269
35	292.9	17225
36	293.9	2454

#	m/z	I
37	294.8	67534
38	295.8	11951
39	296.9	3887
40	308.9	1443
41	310.9	2210
42	316.8	1986
43	324.9	1780
44	331.9	1527
45	332.9	3371
46	339.0	1113
47	352.9	1348
48	354.9	811
49	450.0	833
50	451.0	1920

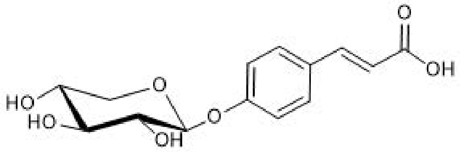

t)

Acceptor: *p*-Coumaric acid

Mass Spectrum List Report

Acquisition Parameter

Ion Source Type	ESI	Ion Polarity	Positive	Alternating Ion Polarity	off
Mass Range Mode	Std/Enhanced	Scan Begin	100 m/z	Scan End	1200 m/z
Capillary Exit	5.0 Volt	Skimmer	40.0 Volt	Trap Drive	50.0
Accumulation Time	2870 µs	Averages	7 Spectra	Auto MS/MS	off

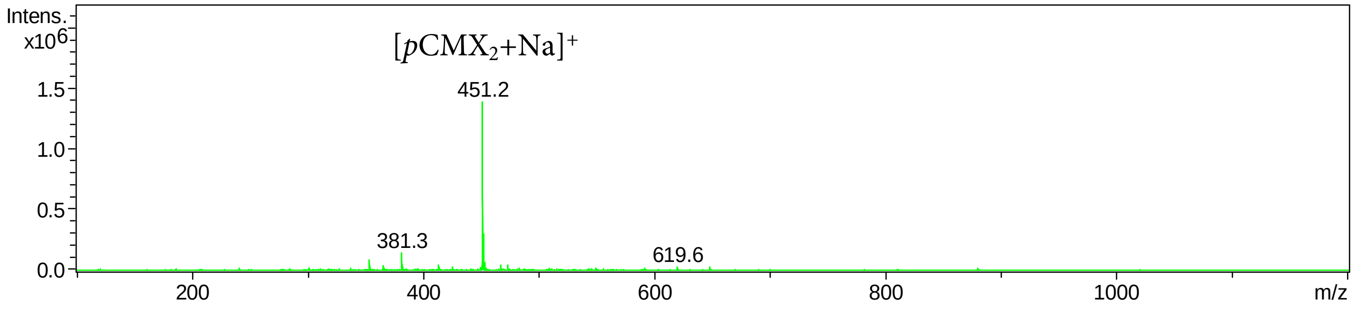

#	m/z	I
1	121.0	10063
2	186.1	10384
3	241.1	16393
4	284.3	13276
5	301.1	19867
6	311.2	10578
7	318.3	10193
8	325.2	9674
9	327.2	10014
10	337.2	15360
11	353.3	86964
12	354.2	17698
13	357.2	9767
14	365.2	38720
15	366.2	16441
16	381.3	145932
17	382.3	30373
18	385.3	12298
19	393.3	14002
20	395.2	11100
21	413.3	42725
22	414.3	14897
23	425.3	28358
24	441.3	15054
25	447.3	10980
26	449.4	22096
27	451.2	1384626
28	452.2	298173
29	453.1	67425
30	454.2	18733
31	465.4	11167
32	467.1	45466
33	469.1	10089
34	469.4	10347
35	473.1	44438
36	474.1	11069
37	481.4	14840
38	483.1	15263
39	487.3	10310
40	509.1	15540
41	511.2	10526
42	515.5	10308
43	543.2	11204
44	545.4	14707
45	549.1	17847
46	555.6	12444
47	591.5	18102
48	619.6	29603
49	647.6	27771
50	879.2	19994

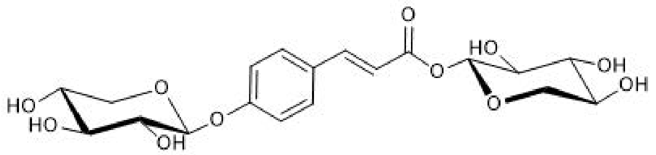

u)

Acceptor: Ferulic acid

Mass Spectrum List Report

Acquisition Parameter

Ion Source Type	ESI	Ion Polarity	Negative	Alternating Ion Polarity	off
Mass Range Mode	Std/Enhanced	Scan Begin	100 m/z	Scan End	1200 m/z
Capillary Exit	-5.0 Volt	Skimmer	-40.0 Volt	Trap Drive	40.0
Accumulation Time	21669 µs	Averages	7 Spectra	Auto MS/MS	off

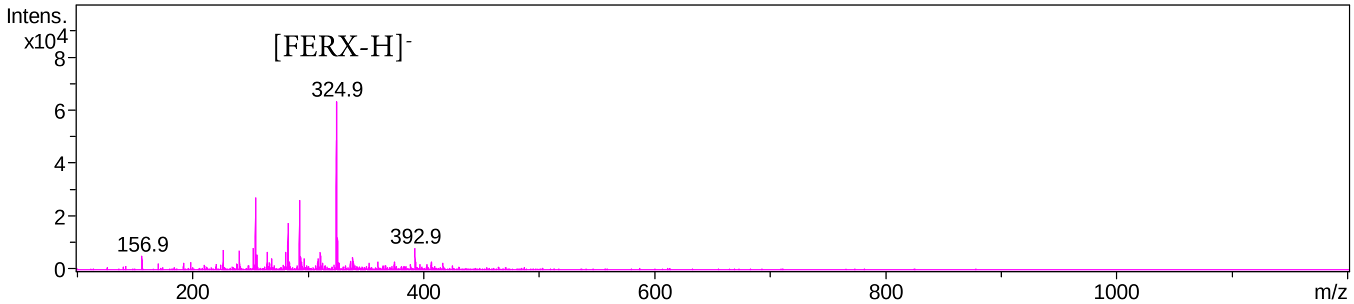

#	m/z	I
1	156.9	5051
2	170.9	2195
3	192.8	2385
4	198.9	2830
5	210.9	1684
6	220.9	1949
7	225.0	1891
8	227.0	7288
9	238.9	2151
10	241.0	7107
11	248.8	1546
12	253.0	7942
13	254.1	1746
14	255.0	27092
15	256.0	5591
16	264.9	6614
17	267.0	2845
18	269.0	4067
19	279.0	1886
20	281.0	6614
21	283.1	17339
22	284.1	3261
23	290.8	1560
24	293.0	26096
25	294.0	5147
26	295.0	3042
27	297.0	4071
28	299.0	1568
29	309.0	4158
30	311.0	6677
31	313.0	2578
32	315.0	1593
33	323.0	1852
34	324.9	63289
35	325.9	12012
36	326.9	2777
37	337.0	3249
38	339.0	4591
39	339.9	2164
40	341.0	1529
41	353.0	2507
42	360.9	2864
43	367.1	1531
44	375.0	3086
45	389.1	2029
46	392.9	7992
47	397.1	2064
48	403.0	1922
49	406.9	2942
50	417.1	2471

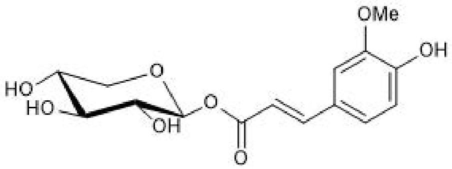

v) **Acceptor: Ferulic acid**

Mass Spectrum List Report

Acquisition Parameter

Ion Source Type	ESI	Ion Polarity	Negative	Alternating Ion Polarity	off
Mass Range Mode	Std/Enhanced	Scan Begin	100 m/z	Scan End	1200 m/z
Capillary Exit	-5.0 Volt	Skimmer	-40.0 Volt	Trap Drive	35.0
Accumulation Time	24724 µs	Averages	7 Spectra	Auto MS/MS	off

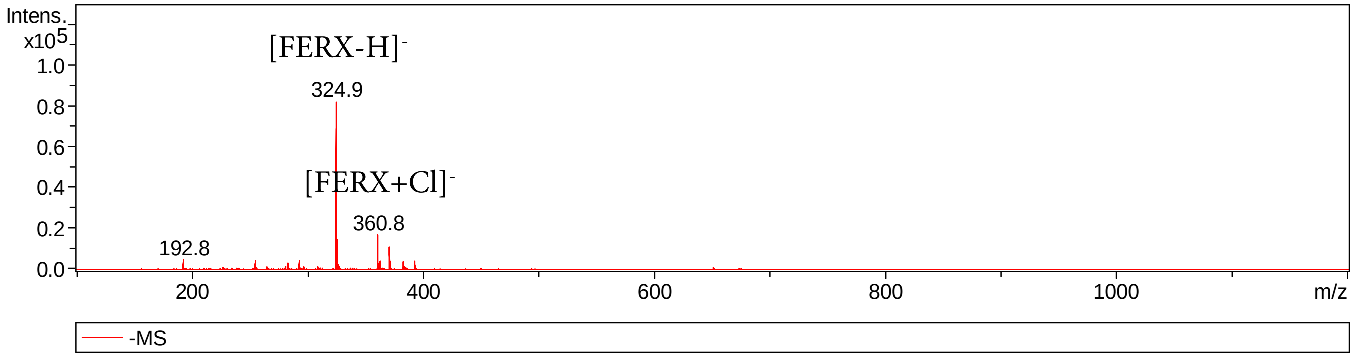

#	m/z	I
1	192.8	4714
2	199.0	529
3	210.9	602
4	212.9	459
5	214.7	441
6	224.8	482
7	227.0	964
8	234.9	594
9	238.9	795
10	241.0	754
11	253.0	842
12	255.0	4517
13	256.0	939
14	264.9	1405
15	266.9	576
16	278.9	447
17	281.0	1284
18	283.1	3092
19	284.1	488
20	292.9	4524
21	293.9	861
22	295.0	435
23	296.9	1257
24	299.0	521
25	306.9	512
26	309.0	1234
27	311.0	813
28	313.1	692
29	322.8	426
30	324.9	81801
31	325.9	14764
32	326.9	2518
33	329.0	534
34	337.0	611
35	339.0	807
36	353.1	512
37	360.8	16990
38	361.8	2839
39	362.8	4257
40	363.9	576
41	365.0	673
42	367.0	551
43	370.9	11052
44	371.9	2893
45	382.9	3872
46	383.8	1250
47	384.9	1081
48	392.9	4127
49	393.9	797
50	651.0	950

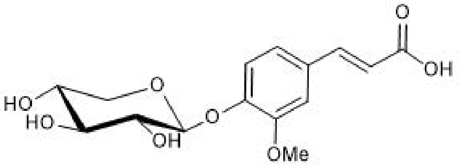

w)

Acceptor: Ferulic acid

Mass Spectrum List Report

Acquisition Parameter

Ion Source Type	ESI	Ion Polarity	Negative	Alternating Ion Polarity	off
Mass Range Mode	Std/Enhanced	Scan Begin	100 m/z	Scan End	1200 m/z
Capillary Exit	-5.0 Volt	Skimmer	-40.0 Volt	Trap Drive	45.0
Accumulation Time	7850 µs	Averages	7 Spectra	Auto MS/MS	off

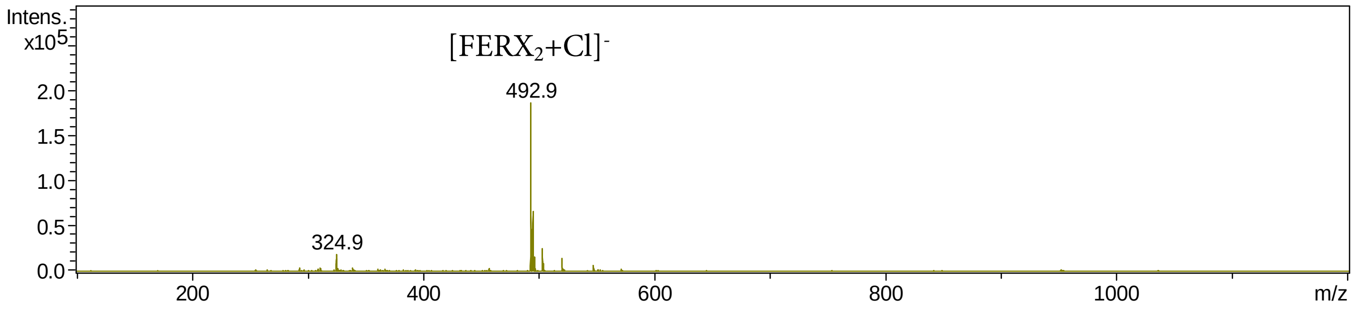

#	m/z	I
1	112.8	1087
2	255.0	1354
3	264.9	1979
4	282.9	1031
5	292.9	3594
6	297.0	1979
7	300.9	1125
8	306.9	1116
9	308.9	2134
10	311.0	4021
11	322.9	1608
12	324.9	18548
13	325.9	2996
14	327.0	1405
15	328.9	1625
16	339.0	3666
17	340.0	1566
18	350.9	1059
19	352.9	1225
20	360.9	2579
21	362.9	1523
22	367.1	2299
23	369.0	1099
24	383.0	1462
25	393.1	1568
26	397.0	1157
27	441.0	1086
28	456.9	2957
29	458.0	1105
30	469.1	1267
31	490.2	1022
32	492.9	186718
33	493.9	46130
34	494.9	66143
35	495.9	16112
36	496.9	3124
37	502.9	24926
38	503.9	8731
39	513.2	1005
40	519.9	14319
41	520.9	2205
42	521.9	1521
43	542.1	1045
44	547.0	6868
45	548.0	1698
46	551.0	1719
47	552.9	1458
48	571.0	2491
49	572.0	1194
50	951.1	1447

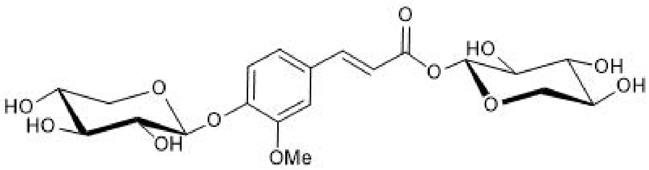

Supplementary Figure 5. ESI-MS spectra of identified glycoconjugates. The clearest spectrum among positive or negative mode is shown for the identified glycoconjugates 2 (a), 4 (b), 6 (c), 8 (d), 10 (e), 11 (f), 13 (g), 15 (h), 17 (i), 19 (j), 21 (k), 23 (l), 25 (m), 27 (n), 29 (o), 31 (p), 33 (q), 35 (r), 36 (s), 37 (t), 39 (u), 40 (v) and 41 (w). The main product adducts are labelled. BSEX: xylosyl derivative of benzeneselenol; BSUX: xylosyl derivative of benzenesulfinic acid; BTTX: xylosyl derivative of 5-(5-bromo-2-thienyl)-1*H*-tetrazole; CINX: xylosyl derivative of *trans*-cinnamic acid; DBrTX: xylosyl derivative of 3,5-dibromo-1,2,4-triazole; EGCGX: xylosyl derivative of epigallocatechin gallate; FERX: xylosyl derivative of ferulic acid; $FERX_2$: dixylosyl derivative of ferulic acid; HBTX: xylosyl derivative of N-hydroxybenzotriazole; HEPX: xylosyl derivative of heptanoic acid; HEXX: xylosyl derivative of hexanoic acid; HZX: xylosyl derivative of hydrazoic acid; $NGAX_\delta$: xylosyl derivative of N-acetyl-L-glutamic acid (δ-carboxyl derivative); pCMX: xylosyl derivative of *p*-coumaric acid; $pCMX_2$: dixylosyl derivative of *p*-coumaric acid; PHOX: xylosyl derivative of phosphoric acid; PhTX: phenyl-β-D-thioxylopyranoside; PPHX: xylosyl derivative of phenylphosphonic acid; VANAP: arabinopyranosyl derivative of vanillin; VANX: xylosyl derivative of vanillin; TPbX: xylosyl derivative of 1*H*-1,2,3-triazolo[4,5-*b*]pyridine.

a) 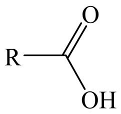

Acceptor: L-Glutamic acid

Mass Spectrum List Report

Acquisition Parameter

Ion Source Type	ESI	Ion Polarity	Negative	Alternating Ion Polarity	off
Mass Range Mode	Ultra Scan	Scan Begin	100 m/z	Scan End	1200 m/z
Capillary Exit	-5.0 Volt	Skimmer	-40.0 Volt	Trap Drive	33.0
Accumulation Time	20000 µs	Averages	14 Spectra	Auto MS/MS	off

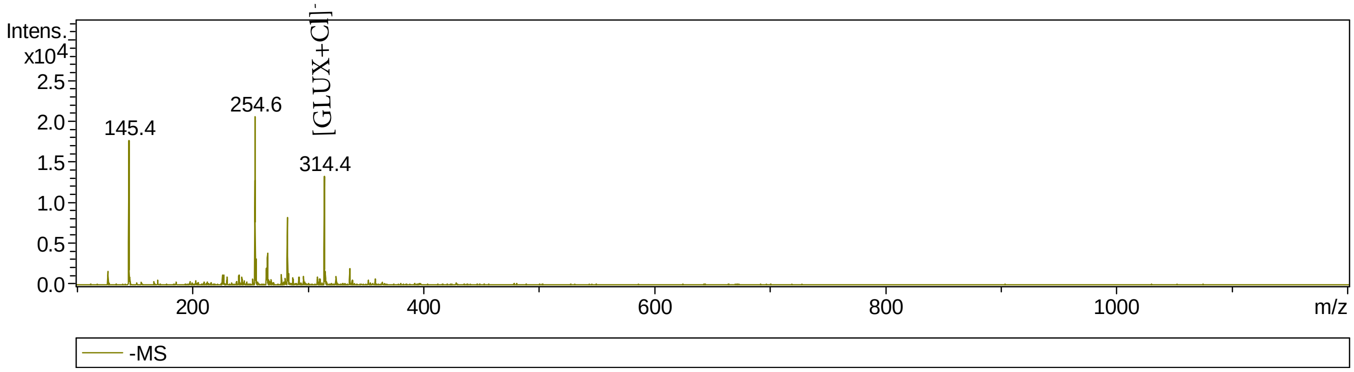

#	m/z	I
1	127.5	1692
2	145.4	17625
3	146.3	958
4	156.5	350
5	167.4	398
6	170.4	571
7	186.4	315
8	198.4	411
9	203.3	531
10	210.4	343
11	213.3	342
12	226.5	1217
13	227.4	1209
14	230.3	989
15	238.4	401
16	240.5	1166
17	241.5	343
18	243.3	968
19	245.2	512
20	247.3	374
21	252.5	726
22	254.6	20541
23	255.6	3200
24	256.5	320
25	264.5	2047
26	265.3	3890
27	266.5	693
28	267.3	424
29	268.5	638
30	277.4	1315
31	279.3	373
32	280.5	835
33	281.3	374
34	282.6	8217
35	283.6	1344
36	287.3	882

#	m/z	I
37	292.5	982
38	296.4	1055
39	297.5	381
40	308.5	932
41	309.3	372
42	310.5	714
43	314.4	13301
44	315.4	1699
45	316.4	385
46	324.5	1059
47	336.5	1969
48	338.6	608
49	352.6	548
50	358.4	717

b)

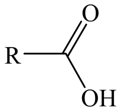

Acceptor: *N*-Acetyl-L-glutamic acid

Mass Spectrum List Report

Acquisition Parameter

Ion Source Type	ESI	Ion Polarity	Negative	Alternating Ion Polarity	off
Mass Range Mode	Std/Enhanced	Scan Begin	100 m/z	Scan End	1200 m/z
Capillary Exit	-5.0 Volt	Skimmer	-40.0 Volt	Trap Drive	35.0
Accumulation Time	14861 µs	Averages	7 Spectra	Auto MS/MS	off

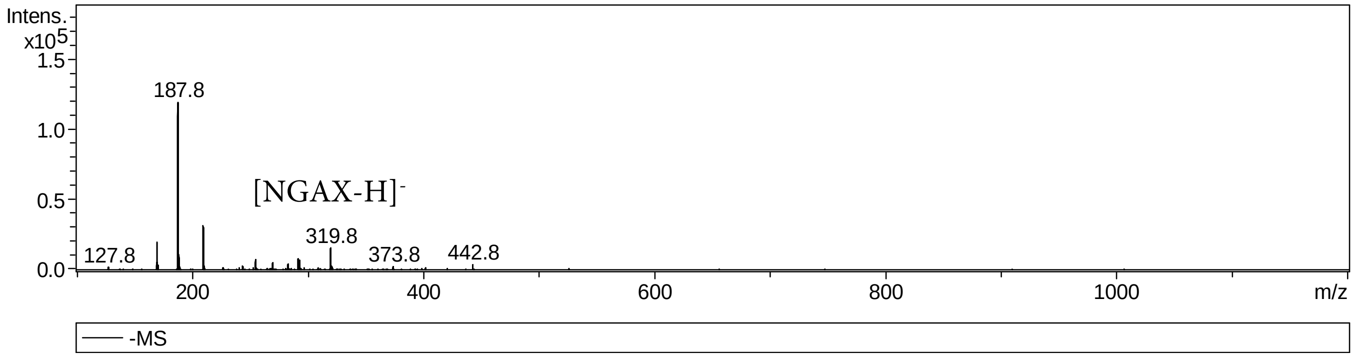

#	m/z	I	#	m/z	I
1	127.8	2139	37	320.8	3061
2	140.7	814	38	321.8	896
3	169.8	19590	39	325.9	764
4	170.8	3391	40	336.9	744
5	187.8	119253	41	351.7	804
6	188.8	11221	42	361.0	834
7	189.8	1853	43	373.8	2276
8	198.9	801	44	398.9	1230
9	209.8	31680	45	401.9	1393
10	210.8	2823	46	420.8	1146
11	226.9	1704	47	436.8	827
12	241.0	1570	48	442.8	3640
13	243.9	2862	49	443.9	733
14	249.8	809	50	525.8	956
15	253.0	1364			
16	255.0	7519			
17	255.9	1699			
18	264.9	1079			
19	267.7	922			
20	269.0	886			
21	269.8	5297			
22	270.7	838			
23	281.0	1127			
24	282.0	730			
25	283.0	4427			
26	284.0	1007			
27	285.0	672			
28	285.8	1048			
29	291.8	7933			
30	292.9	7080			
31	293.9	1120			
32	296.9	1396			
33	301.8	820			
34	309.0	1361			
35	310.9	1216			
36	319.8	15607			

c) 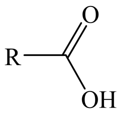

Acceptor: 5-Aminolevulinic acid

Mass Spectrum List Report

Acquisition Parameter

Ion Source Type	ESI	Ion Polarity	Positive	Alternating Ion Polarity	off
Mass Range Mode	Std/Enhanced	Scan Begin	15 m/z	Scan End	800 m/z
Capillary Exit	3.0 Volt	Skimmer	40.0 Volt	Trap Drive	32.0
Accumulation Time	20077 µs	Averages	7 Spectra	Auto MS/MS	off

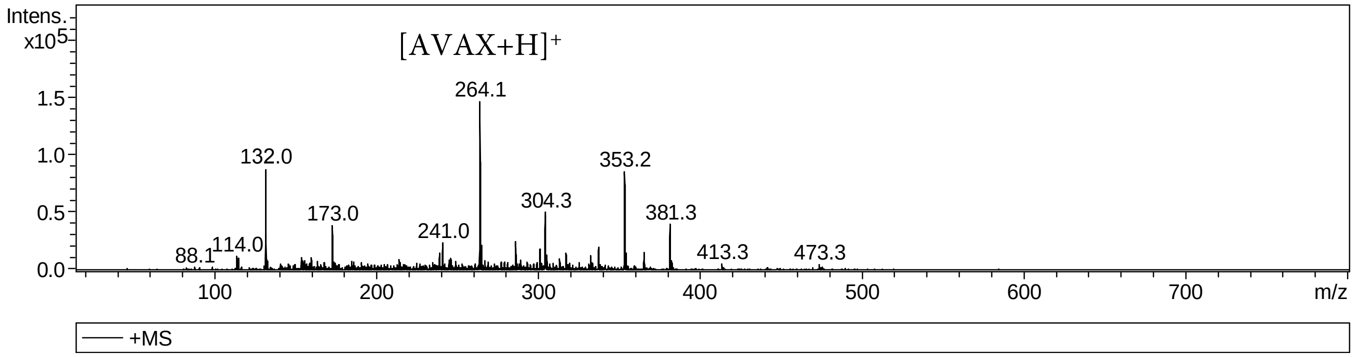

#	m/z	I
1	114.0	11893
2	115.0	10239
3	132.0	87400
4	133.0	8205
5	154.0	10599
6	154.9	7792
7	155.9	7807
8	158.9	5819
9	160.0	11052
10	163.9	7637
11	168.0	6399
12	173.0	38885
13	174.0	6717
14	185.0	7416
15	186.2	7006
16	191.0	6185
17	214.2	8893
18	215.0	6455
19	235.0	6393
20	239.1	14833
21	241.0	23455
22	245.0	8507
23	246.1	10011
24	249.1	7436
25	264.1	146568
26	265.1	21591
27	267.1	7889
28	269.1	6657
29	277.1	6756
30	279.1	7022
31	281.1	6161
32	286.1	24690
33	289.1	8739
34	293.2	6681
35	299.1	6163
36	301.1	18105

#	m/z	I
37	304.3	50589
38	305.3	13255
39	313.2	9896
40	317.1	14952
41	319.2	5854
42	325.2	6613
43	332.3	12549
44	333.3	6314
45	337.3	19569
46	353.2	85546
47	354.3	14943
48	365.3	15482
49	381.3	39859
50	382.3	8053

d) 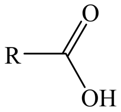

Acceptor: Acetic acid

Mass Spectrum List Report

Acquisition Parameter

Ion Source Type	ESI	Ion Polarity	Positive	Alternating Ion Polarity	off
Mass Range Mode	Std/Enhanced	Scan Begin	50 m/z	Scan End	600 m/z
Capillary Exit	7.0 Volt	Skimmer	40.0 Volt	Trap Drive	25.0
Accumulation Time	84518 µs	Averages	10 Spectra	Auto MS/MS	off

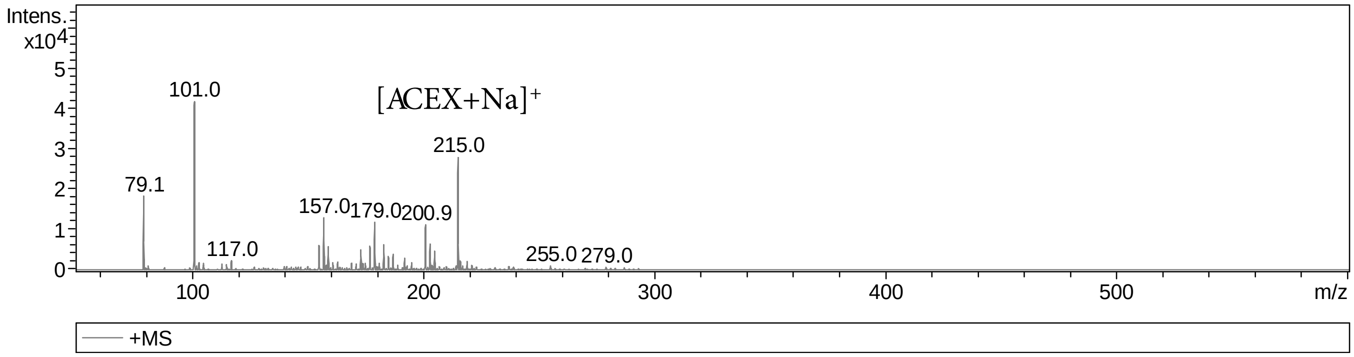

#	m/z	I
1	79.1	18347
2	81.1	1034
3	101.0	41701
4	102.0	959
5	103.0	1781
6	105.0	1682
7	113.0	1409
8	115.0	1304
9	117.0	2255
10	127.0	739
11	140.0	898
12	141.0	821
13	150.1	888
14	155.0	6098
15	157.0	12899
16	158.0	1170
17	159.0	5819
18	160.9	1744
19	163.0	1950
20	164.0	702
21	169.0	1723
22	171.0	1424
23	173.0	4956
24	174.0	1601
25	174.9	1678
26	177.0	5886
27	179.0	11782
28	180.0	834
29	181.0	1680
30	182.9	6247
31	184.9	3436
32	186.9	3814
33	188.9	1107
34	191.9	2955
35	193.0	1069
36	195.0	1876
37	200.9	11189
38	202.9	6456
39	203.9	1198
40	204.9	4689
41	206.9	796
42	209.9	892
43	214.2	1043
44	215.0	27839
45	216.0	2257
46	217.0	958
47	218.9	2106
48	220.9	1175
49	237.0	861
50	255.0	944

e) 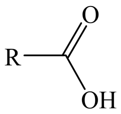

Acceptor: Propionic acid

Mass Spectrum List Report

Acquisition Parameter

Ion Source Type	ESI	Ion Polarity	Positive	Alternating Ion Polarity	off
Mass Range Mode	Std/Enhanced	Scan Begin	15 m/z	Scan End	1000 m/z
Capillary Exit	7.0 Volt	Skimmer	40.0 Volt	Trap Drive	25.0
Accumulation Time	12854 µs	Averages	7 Spectra	Auto MS/MS	off

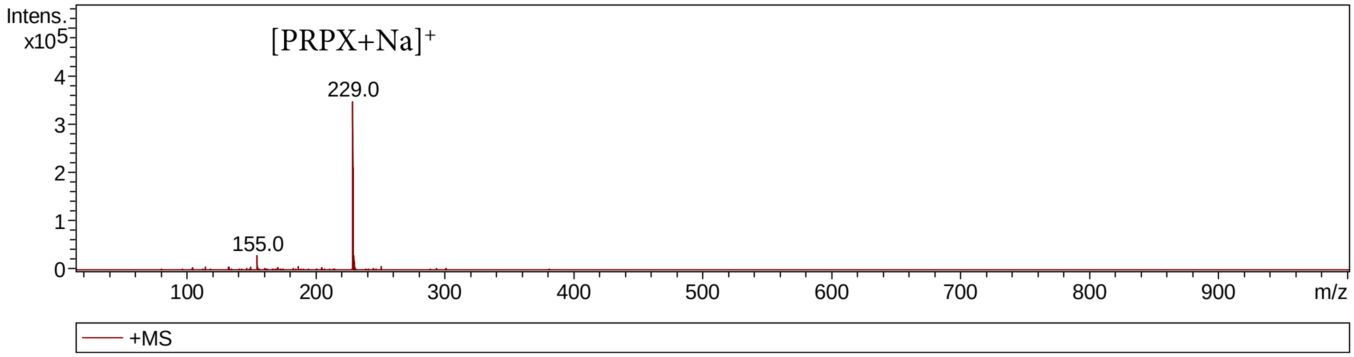

#	m/z	I
1	81.0	1852
2	97.0	1231
3	105.0	5032
4	113.0	1732
5	115.0	5636
6	119.0	1718
7	133.0	5648
8	135.0	1345
9	141.1	1296
10	143.0	1375
11	147.0	3172
12	149.0	1602
13	150.0	6439
14	155.0	29570
15	156.0	1754
16	157.0	2329
17	159.0	1093
18	161.0	2493
19	162.0	1171
20	163.0	1544
21	167.0	1939
22	169.0	1247
23	171.0	4910
24	173.0	1511
25	175.0	1951
26	183.0	2495
27	185.0	2089
28	186.9	6904
29	189.0	1534
30	191.0	1172
31	192.1	1100
32	195.0	1435
33	201.0	1654
34	205.0	4328
35	207.0	2191
36	211.0	1177

#	m/z	I
37	214.1	2439
38	215.1	2030
39	229.0	346803
40	230.0	29712
41	231.0	4301
42	239.1	1393
43	241.0	1504
44	245.0	2721
45	247.0	1318
46	251.0	7197
47	289.1	1206
48	294.0	3342
49	301.1	2709
50	381.1	1563

f) 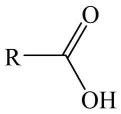

Acceptor: Butyric acid

Mass Spectrum List Report

Acquisition Parameter

Ion Source Type	ESI	Ion Polarity	Positive	Alternating Ion Polarity	off
Mass Range Mode	Std/Enhanced	Scan Begin	50 m/z	Scan End	600 m/z
Capillary Exit	7.0 Volt	Skimmer	40.0 Volt	Trap Drive	25.0
Accumulation Time	81552 µs	Averages	10 Spectra	Auto MS/MS	off

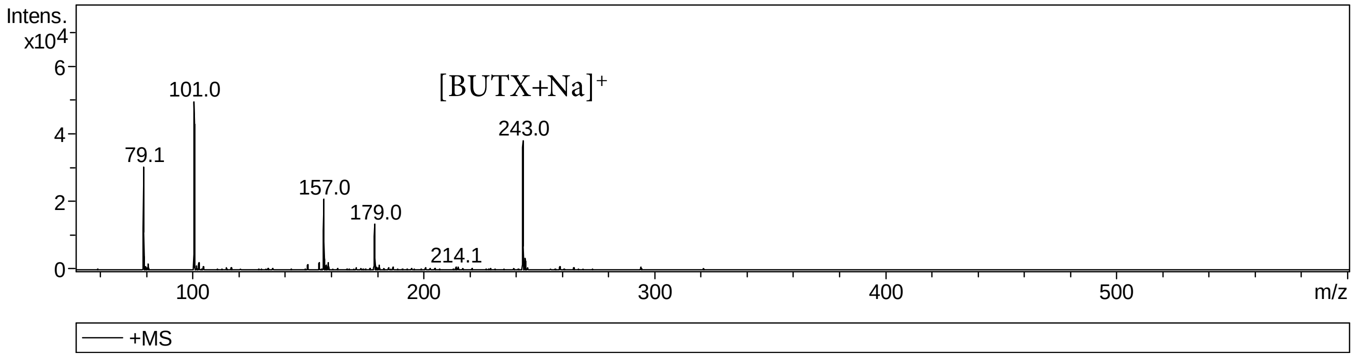

#	m/z	I
1	79.1	30291
2	80.1	934
3	81.1	1692
4	101.0	49555
5	102.0	1156
6	103.0	2161
7	105.0	1095
8	115.0	583
9	117.0	590
10	121.0	343
11	133.0	506
12	135.0	495
13	150.0	1559
14	155.0	2108
15	156.0	288
16	157.0	20866
17	158.0	1341
18	159.0	2051
19	160.9	284
20	163.0	426
21	171.1	695
22	173.0	463
23	175.0	299
24	176.9	441
25	179.0	13513
26	180.0	903
27	181.0	1310
28	183.0	413
29	185.0	616
30	187.0	784
31	191.0	309
32	194.9	368
33	201.0	710
34	202.9	419
35	205.0	486
36	213.0	351

#	m/z	I
37	214.1	792
38	215.1	782
39	217.1	491
40	221.0	375
41	229.1	376
42	239.1	396
43	243.0	37987
44	244.0	3472
45	245.0	707
46	255.0	308
47	259.0	946
48	265.0	721
49	294.1	760
50	321.1	385

g) 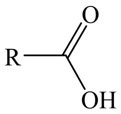

Acceptor: Nicotinic acid

Mass Spectrum List Report

Acquisition Parameter

Ion Source Type	ESI	Ion Polarity	Positive	Alternating Ion Polarity	off
Mass Range Mode	Std/Enhanced	Scan Begin	50 m/z	Scan End	800 m/z
Capillary Exit	7.0 Volt	Skimmer	28.0 Volt	Trap Drive	30.0
Accumulation Time	19111 µs	Averages	10 Spectra	Auto MS/MS	off

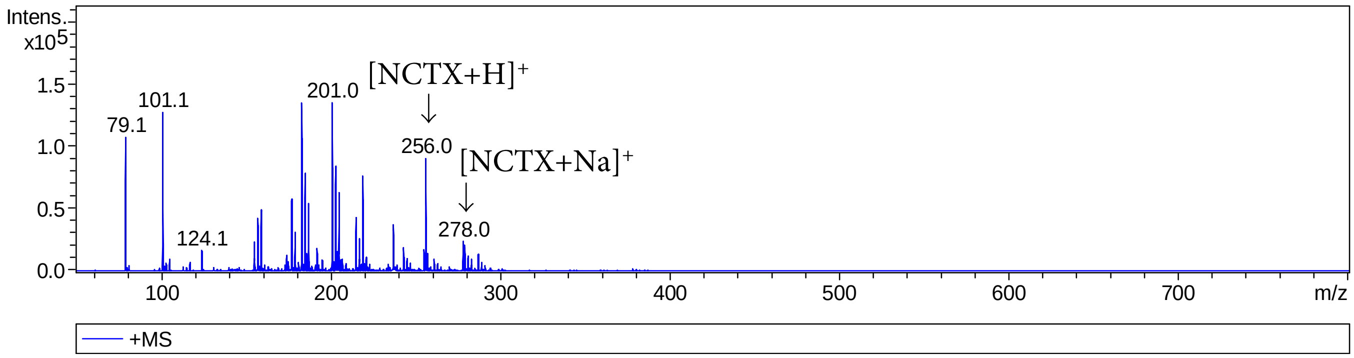

#	m/z	I
1	79.1	107126
2	101.1	126946
3	103.1	6107
4	105.0	9482
5	117.0	6831
6	124.1	16586
7	155.0	23474
8	157.1	42554
9	159.0	48844
10	174.0	12722
11	175.0	7357
12	177.0	57577
13	179.0	31186
14	181.1	6879
15	183.0	134741
16	185.0	78312
17	186.0	13857
18	187.0	54135
19	192.0	18309
20	195.0	9070
21	201.0	134836
22	202.0	7302
23	202.9	83840
24	204.0	15533
25	204.9	62708
26	206.0	9133
27	206.9	9468
28	209.0	5602
29	215.0	42687
30	217.0	25677
31	218.0	5532
32	219.0	76031
33	220.0	5596
34	220.9	11060
35	222.9	5452
36	237.0	36998

#	m/z	I
37	243.0	18608
38	245.0	9810
39	246.9	6572
40	255.0	17271
41	256.0	90116
42	257.0	14263
43	260.9	9610
44	263.0	5909
45	278.0	23774
46	279.0	20966
47	280.9	11909
48	282.9	9386
49	286.9	13744
50	288.9	6967

h) 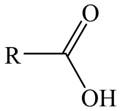

Acceptor: Pentanoic acid

Mass Spectrum List Report

Acquisition Parameter

Ion Source Type	ESI	Ion Polarity	Positive	Alternating Ion Polarity	off
Mass Range Mode	Std/Enhanced	Scan Begin	50 m/z	Scan End	600 m/z
Capillary Exit	7.0 Volt	Skimmer	40.0 Volt	Trap Drive	30.0
Accumulation Time	31758 µs	Averages	10 Spectra	Auto MS/MS	off

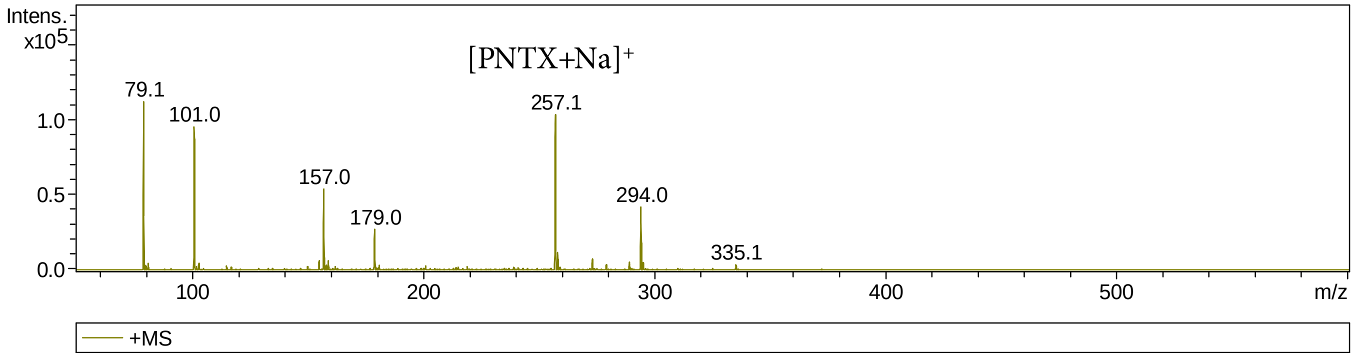

#	m/z	I
1	79.1	111825
2	80.1	2999
3	81.1	4464
4	101.0	95478
5	102.0	2476
6	103.0	4355
7	115.0	2594
8	117.0	1805
9	128.9	1149
10	135.0	1199
11	140.0	959
12	147.0	1029
13	150.0	2199
14	155.0	6244
15	157.0	53944
16	158.0	3041
17	159.0	5935
18	162.0	2153
19	163.0	948
20	177.0	1073
21	179.0	27152
22	180.0	1345
23	180.9	3234
24	189.0	1046
25	198.9	1201
26	201.0	2800
27	213.0	1147
28	214.1	1386
29	215.0	1766
30	217.0	1177
31	219.0	2299
32	235.0	1131
33	237.0	950
34	239.1	1762
35	241.0	1308
36	243.0	1179
37	255.0	1065
38	257.1	103533
39	258.1	11673
40	259.0	2022
41	272.0	970
42	273.1	7556
43	279.1	3466
44	289.1	5485
45	294.0	41902
46	295.0	5007
47	296.0	1014
48	310.0	1000
49	325.0	948
50	335.1	3632

i)

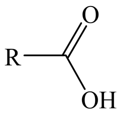

Acceptor: Octanoic acid

Mass Spectrum List Report

Acquisition Parameter

Ion Source Type	ESI	Ion Polarity	Positive	Alternating Ion Polarity	off
Mass Range Mode	Std/Enhanced	Scan Begin	50 m/z	Scan End	1200 m/z
Capillary Exit	1.0 Volt	Skimmer	40.0 Volt	Trap Drive	30.0
Accumulation Time	7316 µs	Averages	10 Spectra	Auto MS/MS	off

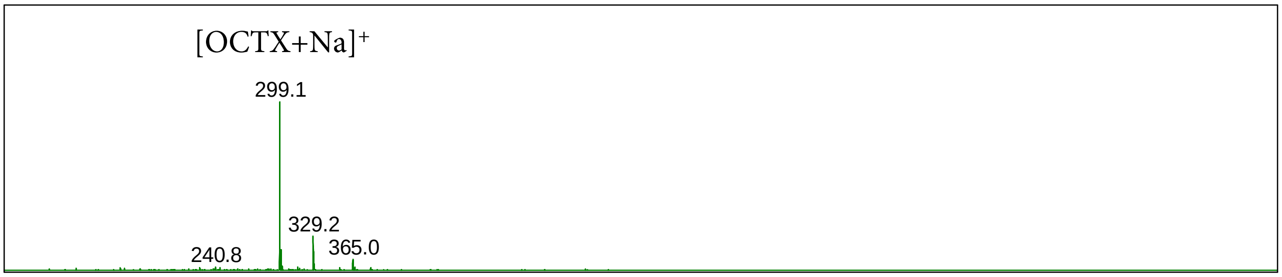

+MS

#	m/z	I
1	91.0	2292
2	105.0	2065
3	114.9	3318
4	132.8	1186
5	134.9	1309
6	148.8	1244
7	154.9	4456
8	158.8	4291
9	167.0	1992
10	172.9	2733
11	180.9	1940
12	183.0	1821
13	185.0	1220
14	186.0	1444
15	189.9	1036
16	197.0	1105
17	200.9	2058
18	202.1	1042
19	202.9	1606
20	204.0	1227
21	211.1	1076
22	213.0	1394
23	216.8	2752
24	220.9	2041
25	222.8	1064
26	223.0	1628
27	224.0	1066
28	226.8	4889
29	229.1	1483
30	230.8	2139
31	237.1	1949
32	239.0	2767
33	240.8	5981
34	243.0	1880
35	244.1	1337
36	244.9	4765

#	m/z	I
37	249.0	1334
38	251.1	1136
39	254.9	1374
40	256.9	1682
41	258.2	1368
42	260.9	3194
43	263.0	1330
44	265.0	1990
45	271.0	2897
46	275.9	1376
47	277.1	2030
48	279.1	3086
49	281.1	1059
50	286.3	1449
51	287.1	1531
52	288.3	2419
53	289.3	2990
54	291.1	2275
55	293.1	1572
56	297.0	1590
57	299.1	301725
58	300.1	36980
59	301.0	9013
60	302.1	2105
61	307.1	2840
62	308.1	1199
63	309.0	1163
64	310.1	1662
65	311.1	1397
66	313.2	1018
67	315.1	5793
68	316.0	1178
69	317.1	3468
70	319.1	1094
71	321.0	2682
72	324.0	1751

#	m/z	I
73	329.2	60911
74	330.2	11846
75	331.2	1652
76	337.0	1057
77	353.2	4479
78	354.1	1537
79	357.2	1770
80	365.0	19663
81	366.1	3536
82	367.1	6410
83	368.4	1068
84	369.1	1300
85	381.3	5069
86	382.4	1573
87	387.1	1262
88	393.1	1120
89	396.1	1239
90	409.1	1092
91	435.2	1619
92	441.2	1134
93	442.2	1141
94	517.7	1428
95	520.8	1236
96	538.2	1196
97	575.2	3179
98	577.3	1036
99	595.8	1109
100	766.4	994

j)

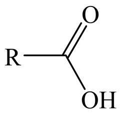

Acceptor: Cholic acid

Display Report: ZOOM

Acquisition Parameter

Ion Source Type	ESI	Ion Polarity	Positive	Alternating Ion Polarity	off
Mass Range Mode	Std/Enhanced	Scan Begin	50 m/z	Scan End	800 m/z
Capillary Exit	7.0 Volt	Skimmer	28.0 Volt	Trap Drive	40.0
Accumulation Time	10631 µs	Averages	10 Spectra	Auto MS/MS	off

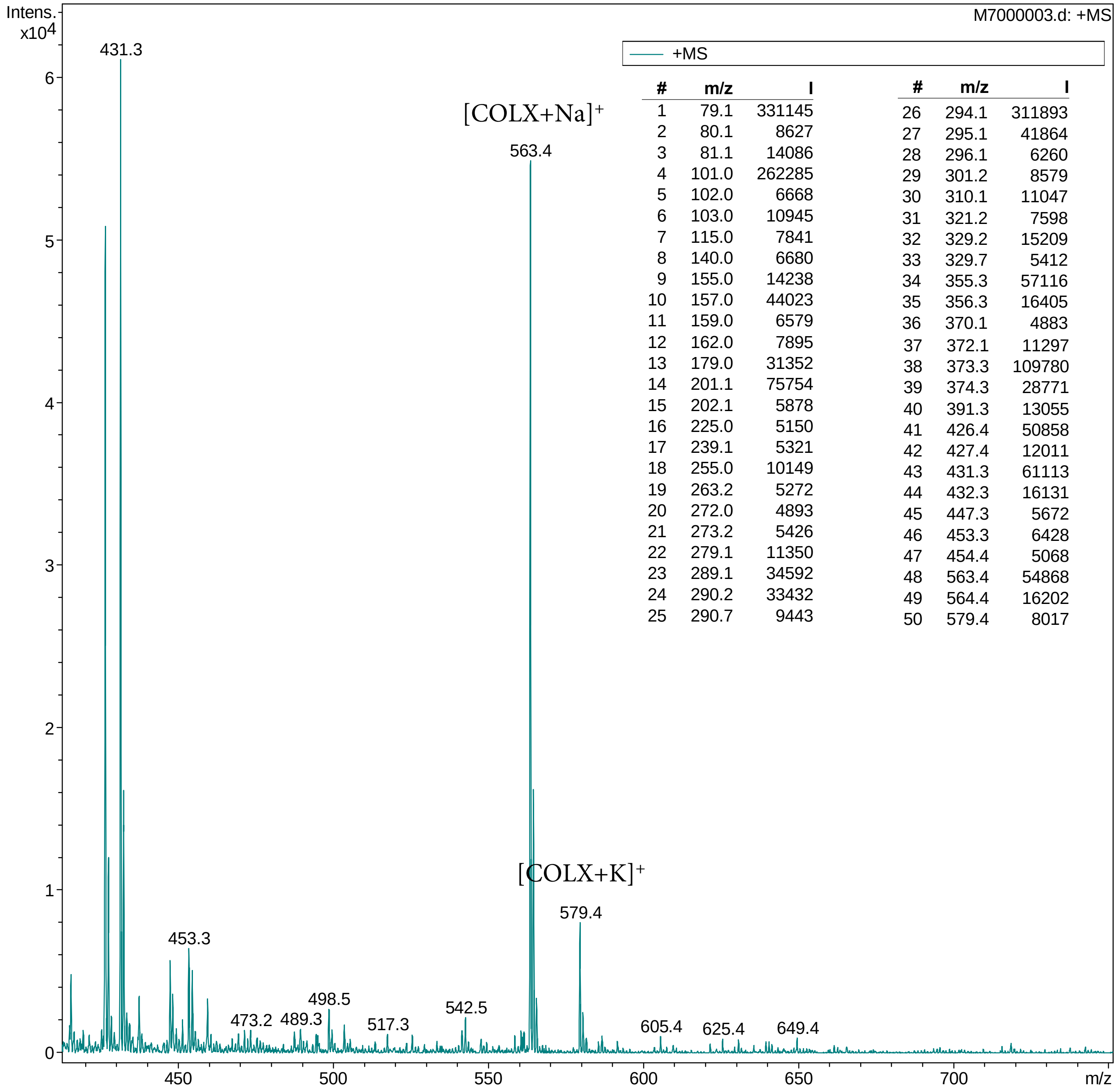

#	m/z	I	#	m/z	I
1	79.1	331145	26	294.1	311893
2	80.1	8627	27	295.1	41864
3	81.1	14086	28	296.1	6260
4	101.0	262285	29	301.2	8579
5	102.0	6668	30	310.1	11047
6	103.0	10945	31	321.2	7598
7	115.0	7841	32	329.2	15209
8	140.0	6680	33	329.7	5412
9	155.0	14238	34	355.3	57116
10	157.0	44023	35	356.3	16405
11	159.0	6579	36	370.1	4883
12	162.0	7895	37	372.1	11297
13	179.0	31352	38	373.3	109780
14	201.1	75754	39	374.3	28771
15	202.1	5878	40	391.3	13055
16	225.0	5150	41	426.4	50858
17	239.1	5321	42	427.4	12011
18	255.0	10149	43	431.3	61113
19	263.2	5272	44	432.3	16131
20	272.0	4893	45	447.3	5672
21	273.2	5426	46	453.3	6428
22	279.1	11350	47	454.4	5068
23	289.1	34592	48	563.4	54868
24	290.2	33432	49	564.4	16202
25	290.7	9443	50	579.4	8017

k) 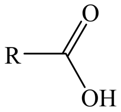

Acceptor: Gallic acid

Mass Spectrum List Report

Acquisition Parameter

Ion Source Type	ESI	Ion Polarity	Positive	Alternating Ion Polarity	off
Mass Range Mode	Std/Enhanced	Scan Begin	150 m/z	Scan End	1200 m/z
Capillary Exit	2.0 Volt	Skimmer	40.0 Volt	Trap Drive	35.0
Accumulation Time	8425 µs	Averages	7 Spectra	Auto MS/MS	off

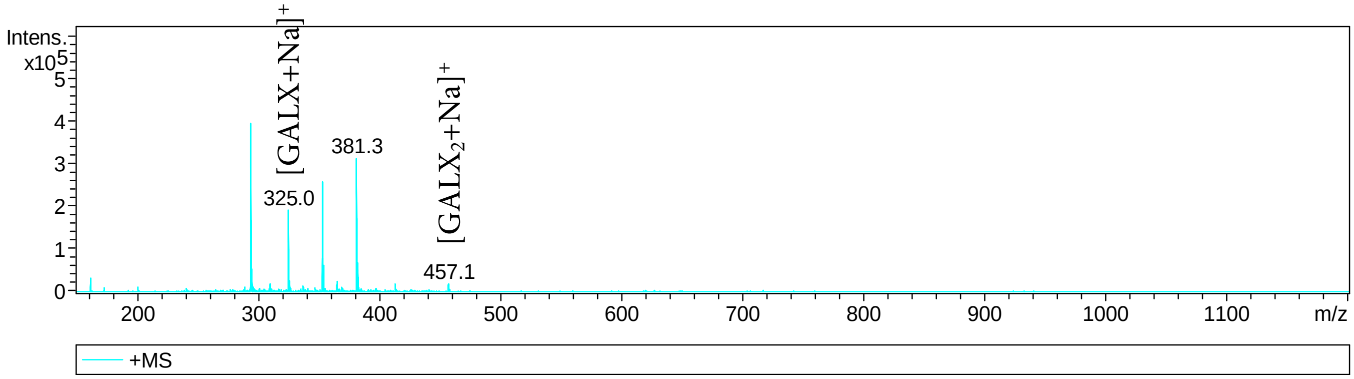

#	m/z	I
1	162.0	32282
2	173.0	9106
3	201.0	11476
4	241.1	7711
5	265.1	4796
6	279.1	5053
7	288.3	4693
8	289.1	10852
9	293.2	4969
10	294.0	394588
11	295.0	53038
12	296.0	11277
13	297.2	6833
14	301.1	8024
15	305.2	6667
16	309.2	6788
17	310.0	19226
18	311.2	6594
19	317.2	7189
20	319.2	5396
21	323.2	4835
22	325.0	192205
23	326.0	25675
24	327.0	8150
25	335.2	6207
26	337.2	13404
27	339.2	4855
28	341.0	8752
29	347.1	8837
30	351.2	5433
31	353.2	257571
32	354.2	62937
33	355.3	8746
34	365.2	25348
35	366.1	5182
36	367.2	6268
37	369.2	11638
38	370.2	5868
39	381.3	311447
40	382.3	68481
41	383.2	9851
42	385.0	6228
43	393.2	5053
44	397.3	8692
45	413.3	19156
46	414.3	5600
47	426.1	5189
48	441.2	4638
49	457.1	18014
50	458.1	5879

1)

Acceptor: Caffeic acid

Max. 657.5 counts.

-TOF MS: 1.000 to 4.000 min from QS22685 (recalibrated).wiff
a=3.57352301956509670e-004, t0=5.88566563165574910e+001 R;

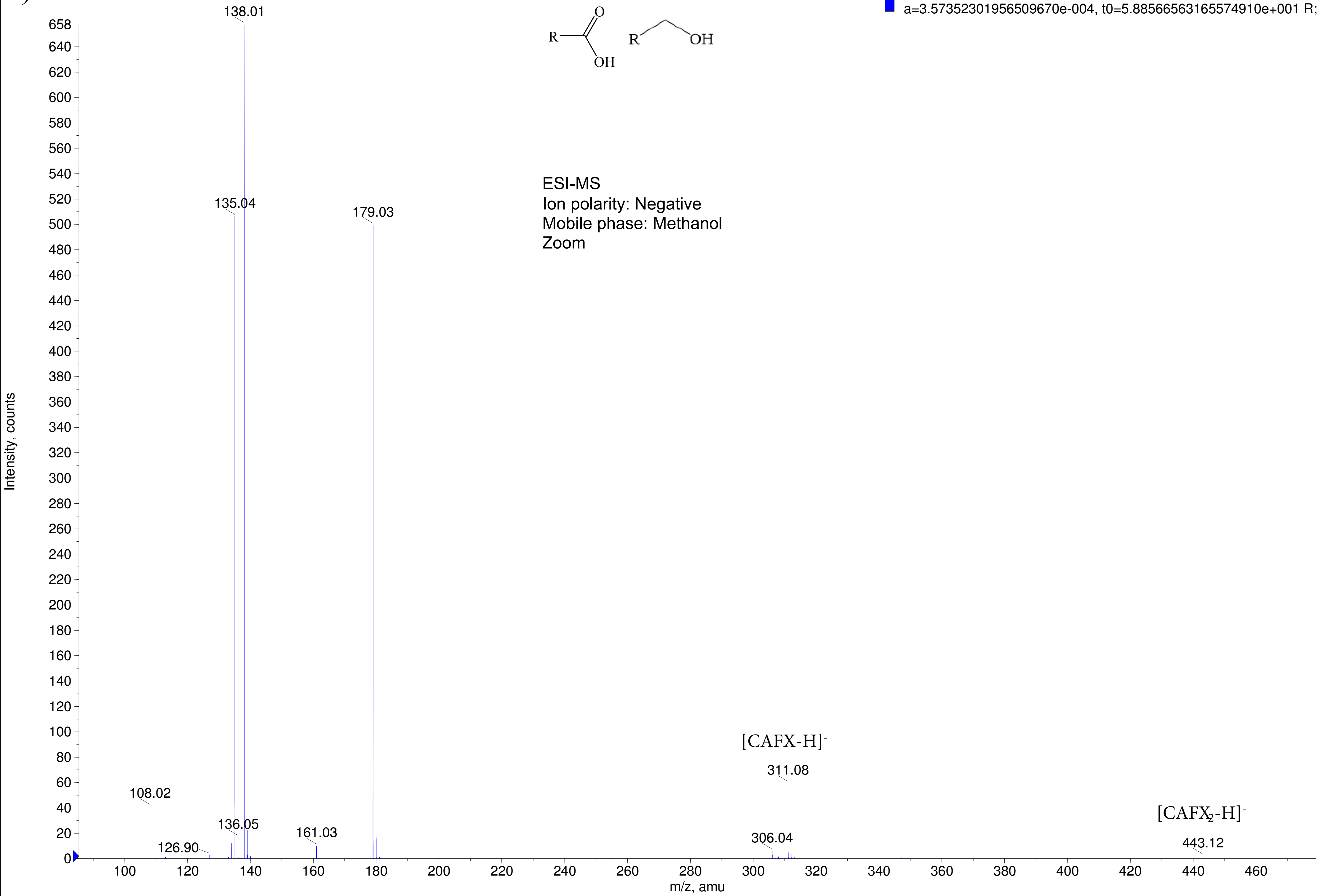

Peak List for " -TOF MS: 1.000 to 4.000 min from QS22685 (recalibrated).wiff a=3.57352301956509670e-004, t0=5.88566563165574910e+001 R;"

	Centroid mass (amu)	Centroid intensity	Charges	%Intensity
1	108.0214	62.7836	1	6.2816
2	109.0256	3.4936	1	0.3126
3	126.9048	3.6501	Undefined	0.4310
4	134.0368	17.6851	Undefined	1.8720
5	135.0420	593.6243	1	77.0004
6	136.0458	23.3103	1	2.5526
7	138.0131	777.5304	1	100.0000
8	139.0184	30.3766	1	3.4122
9	161.0266	14.9466	Undefined	1.5368
10	179.0284	606.8490	Undefined	75.9392
11	180.0332	26.7634	Undefined	2.7417
12	306.0412	11.3775	Undefined	0.9192
13	311.0770	92.7661	Undefined	9.0358
14	312.0815	6.4770	Undefined	0.5512
15	443.1221	3.8665	Undefined	0.3126

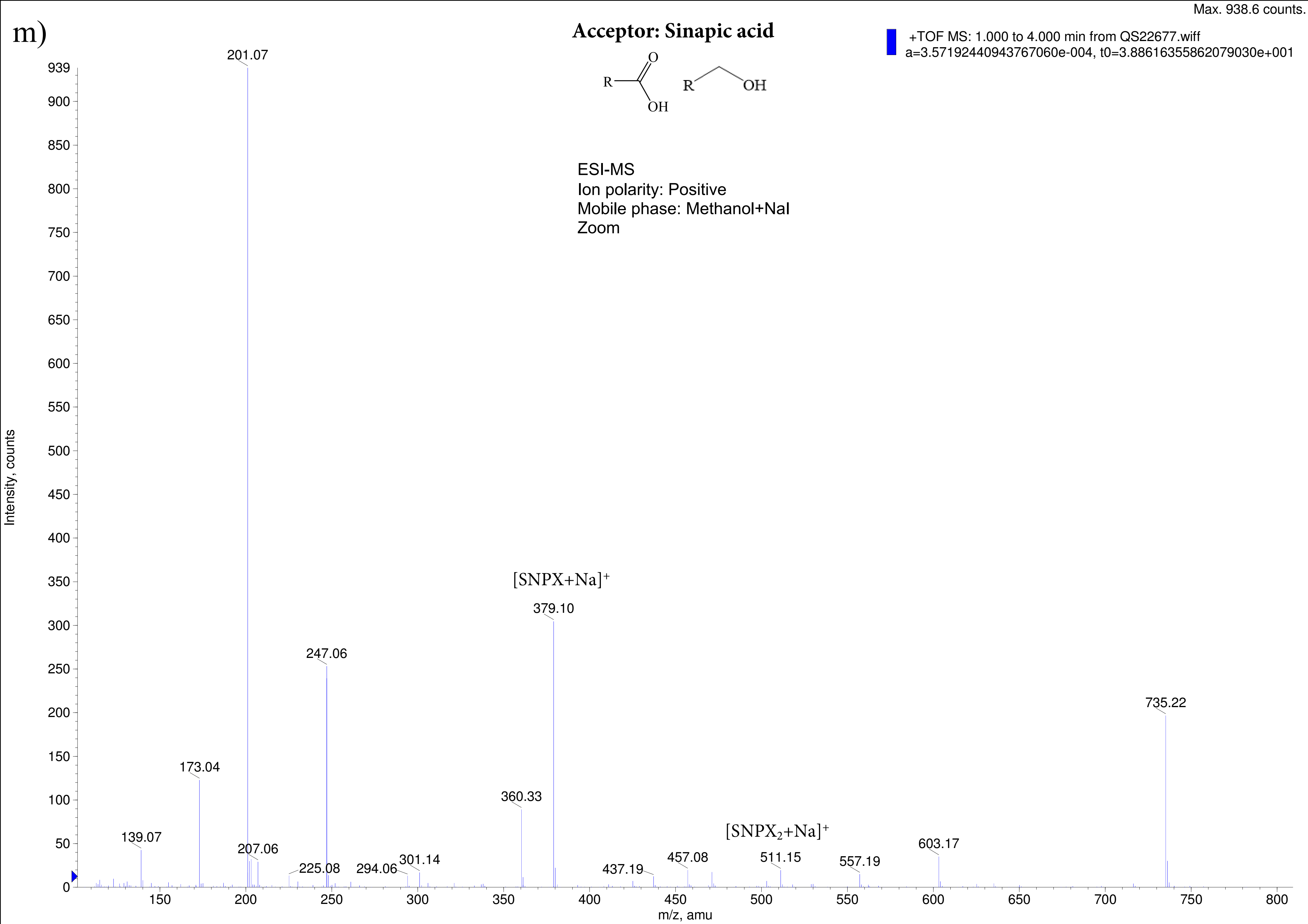
m)
Acceptor: Sinapic acid
Max. 938.6 counts.
+TOF MS: 1.000 to 4.000 min from QS22677.wiff
a=3.57192440943767060e-004, t0=3.88616355862079030e+001
R
O
OH
R
OH
ESI-MS
Ion polarity: Positive
Mobile phase: Methanol+NaI
Zoom
[SNPX+Na]+
[SNPX2+Na]+
201.07
379.10
247.06
735.22
173.04
360.33
139.07
207.06
225.08
294.06
301.14
437.19
457.08
511.15
557.19
603.17
Intensity, counts
m/z, amu

Peak List for " +TOF MS: 1.000 to 4.000 min from QS22677.wiff a=3.57192440943767060e-004, t0=3.88616355862079030e+001"

	Centroid mass (amu)	Centroid intensity	Charges	%Intensity
1	139.0739	54.6022	Undefined	4.5485
2	173.0428	165.1768	Undefined	13.0850
3	201.0712	1181.7974	Undefined	100.0000
4	202.0750	41.2081	1	3.2034
5	203.0668	46.9788	1	3.4430
6	207.0633	42.2376	Undefined	3.1145
7	225.0762	19.9033	Undefined	1.4133
8	247.0595	333.8379	1	26.9752
9	248.0618	19.9540	1	1.4704
10	294.0585	21.1252	Undefined	1.3921
11	301.1425	26.7431	Undefined	1.7989
12	360.3267	133.6234	Undefined	9.5042
13	379.1005	455.7956	Undefined	32.4384
14	380.1029	37.6317	Undefined	2.3752
15	437.1928	20.7993	Undefined	1.3274
16	457.0794	34.6924	Undefined	2.0991
17	471.1272	31.3389	Undefined	1.8583
18	511.1452	35.8361	Undefined	2.0832
19	557.1860	27.5847	Undefined	1.5940
20	603.1730	64.4567	Undefined	3.7450
21	735.2158	353.3407	Undefined	20.9534
22	736.2175	58.6151	Undefined	3.2105

n)

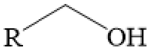

Acceptor: Quercetin

Mass Spectrum List Report

Acquisition Parameter

Ion Source Type	ESI	Ion Polarity	Negative	Alternating Ion Polarity	off
Mass Range Mode	Ultra Scan	Scan Begin	100 m/z	Scan End	1000 m/z
Capillary Exit	-10.0 Volt	Skimmer	-40.0 Volt	Trap Drive	44.0
Accumulation Time	110000 µs	Averages	14 Spectra	Auto MS/MS	off

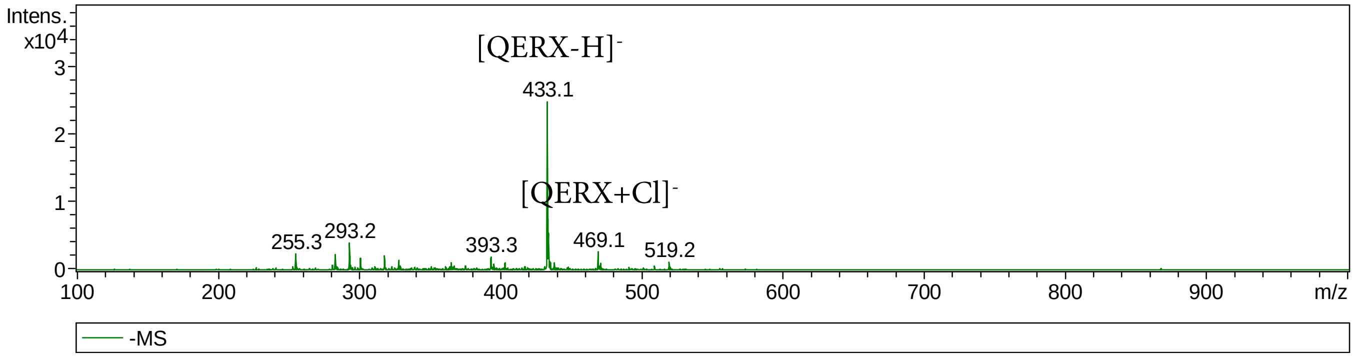

#	m/z	I
1	253.2	523
2	255.3	2423
3	256.3	326
4	269.2	323
5	281.3	725
6	283.3	2321
7	284.3	478
8	285.2	319
9	293.2	4007
10	294.2	663
11	297.2	408
12	301.1	1751
13	309.2	316
14	311.2	521
15	318.1	2083
16	323.2	466
17	325.2	349
18	328.2	1433
19	329.2	588
20	339.3	402
21	341.2	348
22	351.3	467
23	353.3	342
24	361.2	504
25	364.2	488
26	365.3	1119
27	366.2	402
28	367.3	556
29	375.3	636
30	393.3	1948
31	394.3	402
32	395.3	851
33	403.3	1118
34	417.1	544
35	419.2	318
36	431.2	485
37	433.1	24847
38	434.1	5472
39	435.1	1310
40	438.1	1046
41	439.2	340
42	448.1	406
43	469.1	2694
44	470.0	562
45	471.0	1006
46	491.0	387
47	501.1	329
48	509.1	565
49	519.2	1135
50	520.2	393

Acceptor: Quercetin

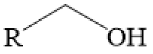

Mass Spectrum List Report

Acquisition Parameter

Ion Source Type	ESI	Ion Polarity	Negative	Alternating Ion Polarity	off
Mass Range Mode	Ultra Scan	Scan Begin	100 m/z	Scan End	1000 m/z
Capillary Exit	-10.0 Volt	Skimmer	-40.0 Volt	Trap Drive	44.0
Accumulation Time	20000 µs	Averages	14 Spectra	Auto MS/MS	off

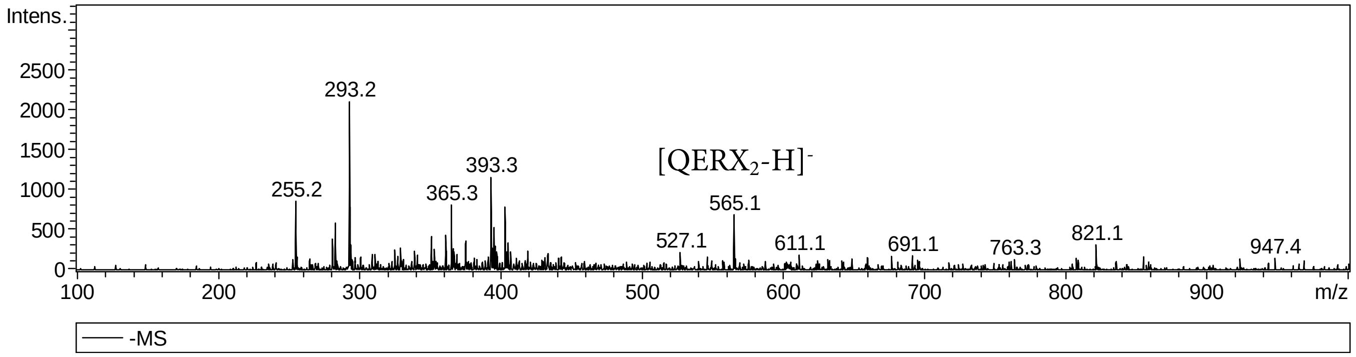

#	m/z	I
1	255.2	860
2	256.2	165
3	281.3	388
4	283.2	590
5	293.2	2098
6	294.2	314
7	297.2	159
8	301.1	164
9	309.2	195
10	311.2	197
11	325.2	251
12	327.3	168
13	329.2	278
14	339.2	237
15	341.2	184
16	351.2	418
17	353.2	260
18	361.2	438
19	365.3	811
20	366.4	269
21	367.3	225
22	369.2	193
23	375.3	364
24	381.3	145
25	391.3	164
26	393.3	1158
27	394.3	274
28	395.3	533
29	396.2	300
30	397.3	230
31	403.3	785
32	404.3	224
33	405.3	342
34	407.2	226
35	411.2	149
36	419.2	233
37	431.3	154
38	433.3	201
39	441.1	151
40	442.9	165
41	527.1	216
42	546.0	160
43	565.1	693
44	611.1	191
45	659.5	159
46	676.5	175
47	691.1	178
48	807.1	150
49	821.1	315
50	854.4	167

o)

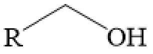

Acceptor: Silibinin

Mass Spectrum List Report

Acquisition Parameter

Ion Source Type	ESI	Ion Polarity	Negative	Alternating Ion Polarity	off
Mass Range Mode	Std/Enhanced	Scan Begin	200 m/z	Scan End	1200 m/z
Capillary Exit	-110.0 Volt	Skimmer	-40.0 Volt	Trap Drive	75.0
Accumulation Time	7086 µs	Averages	14 Spectra	Auto MS/MS	off

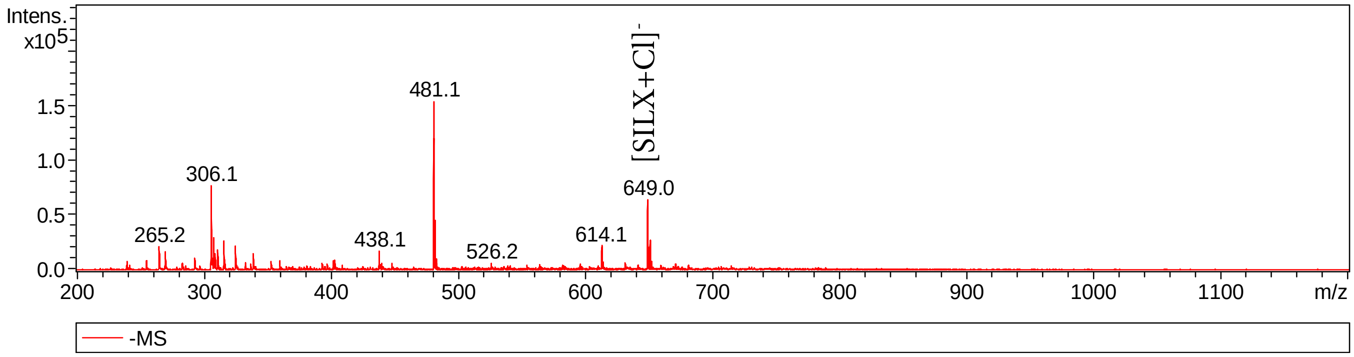

#	m/z	I	#	m/z	I
1	240.0	7732	37	564.1	4892
2	242.0	4341	38	582.1	4283
3	255.3	8645	39	596.2	5410
4	265.2	21152	40	613.1	22565
5	270.1	16502	41	614.1	7130
6	283.3	6017	42	631.4	6819
7	286.0	4011	43	641.4	4509
8	293.2	10707	44	649.0	64189
9	306.1	76860	45	650.0	20880
10	307.1	10863	46	651.0	26930
11	308.0	29224	47	652.0	7943
12	309.2	14730	48	659.4	4253
13	311.2	18169	49	671.0	5528
14	316.1	26571	50	681.0	4167
15	317.1	5215			
16	325.2	21787			
17	326.2	4480			
18	333.1	6394			
19	337.2	5369			
20	339.2	14610			
21	353.2	7531			
22	360.1	8238			
23	393.3	5917			
24	397.2	5669			
25	402.1	8115			
26	403.2	8705			
27	409.1	4255			
28	438.1	16969			
29	439.1	4746			
30	440.1	6268			
31	448.1	6220			
32	481.1	153151			
33	482.0	45012			
34	483.1	9983			
35	526.2	6041			
36	554.2	4484			

p)

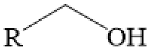

Acceptor: Phloretin

Mass Spectrum List Report

Analysis Info

Analysis Name	D:\Data\201901\PhLX0004.d	Acquisition Date	1/8/2019 1:18:21 PM
Method	ESI_Pos_Valentina_01.m	Operator	CAI
Sample Name	PhLX	Instrument	HCTultra PTM Discovery System
Comment	Directo. EN LIQUIDO + MeOH		

Acquisition Parameter

Ion Source Type	ESI	Ion Polarity	Negative	Alternating Ion Polarity	off
Mass Range Mode	Std/Enhanced	Scan Begin	100 m/z	Scan End	1500 m/z
Capillary Exit	-94.0 Volt	Skimmer	-40.0 Volt	Trap Drive	50.7
Accumulation Time	17655 µs	Averages	9 Spectra	Auto MS/MS	off

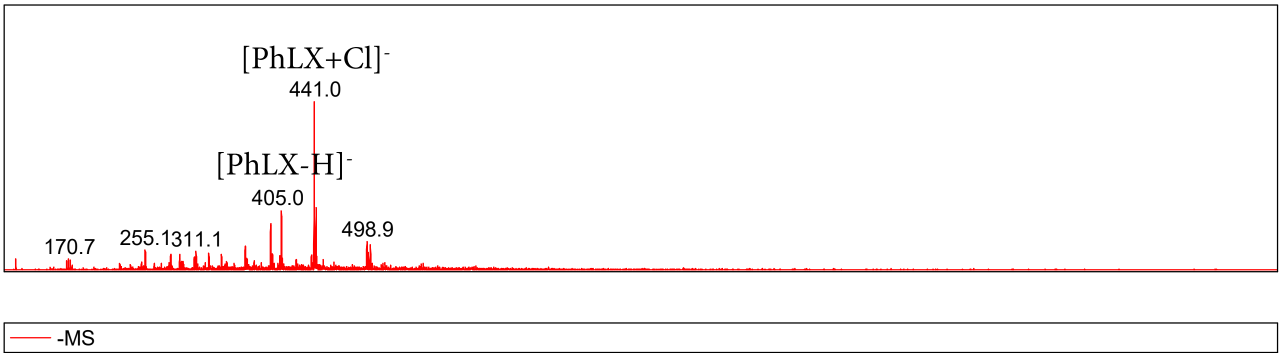

#	m/z	I	#	m/z	I	#	m/z	I
1	112.8	2416	37	375.0	2011	73	450.9	2309
2	168.6	2064	38	377.0	1083	74	452.0	962
3	170.7	2514	39	381.1	889	75	459.0	1090
4	172.7	2169	40	383.2	1592	76	462.9	1700
5	174.7	999	41	391.1	970	77	465.0	1018
6	227.1	1448	42	393.2	10389	78	468.0	875
7	238.9	1138	43	394.2	2742	79	482.9	1131
8	251.0	1757	44	395.2	3667	80	485.0	949
9	253.1	878	45	396.2	1068	81	498.9	6259
10	255.1	4531	46	397.1	1486	82	499.9	1902
11	265.0	1490	47	401.0	1445	83	500.9	3889
12	272.9	1542	48	402.1	932	84	501.9	1056
13	281.2	1568	49	403.1	3200	85	503.0	5555
14	283.2	3492	50	404.2	1458	86	503.9	1128
15	284.1	1105	51	405.0	13227	87	504.9	1499
16	293.1	3544	52	406.0	2614	88	507.0	918
17	295.1	1886	53	407.0	1405	89	514.9	1250
18	297.0	1840	54	417.0	896	90	516.9	1429
19	309.1	2926	55	421.1	2287	91	518.9	1677
20	311.1	4254	56	422.1	1111	92	519.0	1598
21	312.9	1285	57	423.0	1385	93	520.0	966
22	321.1	1584	58	425.0	1016	94	520.9	955
23	325.1	3721	59	426.9	1210	95	525.0	989
24	326.1	873	60	428.7	1029	96	556.8	927
25	329.0	916	61	428.9	1083	97	558.9	1354
26	335.1	964	62	435.0	999	98	560.9	1458
27	339.1	3419	63	437.9	3395	99	576.9	921
28	342.7	1230	64	439.0	1674	100	618.8	915
29	342.9	1273	65	440.0	1168			
30	344.8	1850	66	441.0	37464			
31	352.9	1459	67	442.0	8407			
32	365.2	5381	68	443.0	13892			
33	366.1	1352	69	443.9	3014			
34	367.1	2591	70	445.0	1123			
35	369.0	1213	71	446.9	1232			
36	373.0	959	72	448.0	1013			

q)

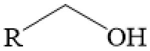

Acceptor: Methyl 4-hydroxybenzoate

Mass Spectrum List Report

Acquisition Parameter

Ion Source Type	ESI	Ion Polarity	Positive	Alternating Ion Polarity	off
Mass Range Mode	Ultra Scan	Scan Begin	100 m/z	Scan End	1000 m/z
Capillary Exit	10.0 Volt	Skimmer	40.0 Volt	Trap Drive	35.0
Accumulation Time	8042 µs	Averages	14 Spectra	Auto MS/MS	off

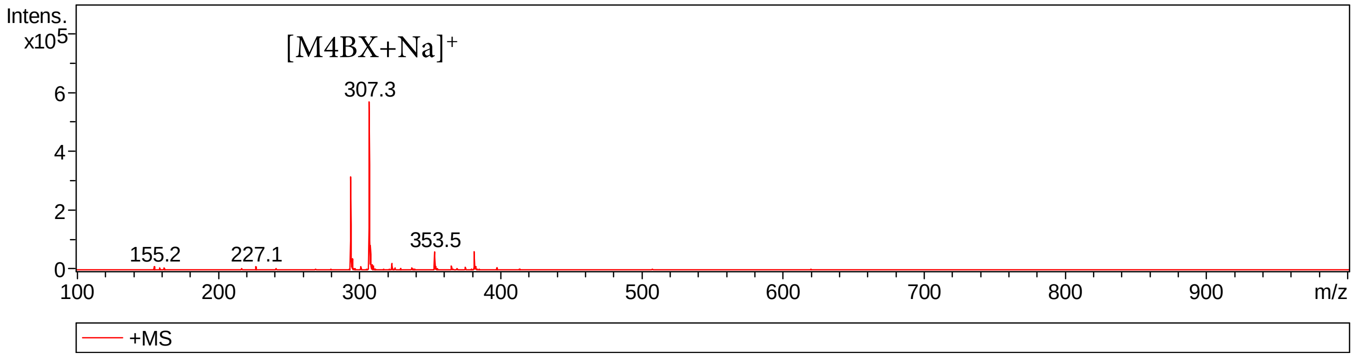

#	m/z	I
1	155.2	11289
2	159.1	6298
3	162.2	7765
4	217.0	4578
5	227.1	10665
6	241.3	5034
7	257.1	1803
8	265.3	1858
9	269.3	2863
10	280.2	4037
11	293.3	3009
12	294.2	314368
13	295.2	38325
14	296.2	4768
15	297.4	4891
16	301.4	10640
17	302.3	1776
18	304.5	1763
19	305.4	2324
20	307.3	569874
21	308.3	82517
22	309.3	17319
23	310.3	13667
24	311.3	2545
25	317.4	1920
26	321.4	2102
27	323.3	22168
28	324.3	3510
29	325.4	6689
30	329.4	4376
31	337.5	7206
32	338.4	2201
33	339.5	1924
34	353.5	61966
35	354.5	10676
36	355.5	4583

#	m/z	I
37	365.4	14182
38	366.4	3648
39	369.4	5290
40	375.3	8590
41	379.5	2296
42	381.5	61902
43	382.5	12547
44	383.5	4034
45	385.3	2591
46	397.5	7312
47	413.5	3549
48	414.4	1760
49	507.5	3035
50	619.6	2962

r)

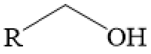

Acceptor: Pyrogallol

Mass Spectrum List Report

Acquisition Parameter

Ion Source Type	ESI	Ion Polarity	Negative	Alternating Ion Polarity	off
Mass Range Mode	Ultra Scan	Scan Begin	100 m/z	Scan End	1000 m/z
Capillary Exit	-10.0 Volt	Skimmer	-40.0 Volt	Trap Drive	35.0
Accumulation Time	20000 µs	Averages	14 Spectra	Auto MS/MS	off

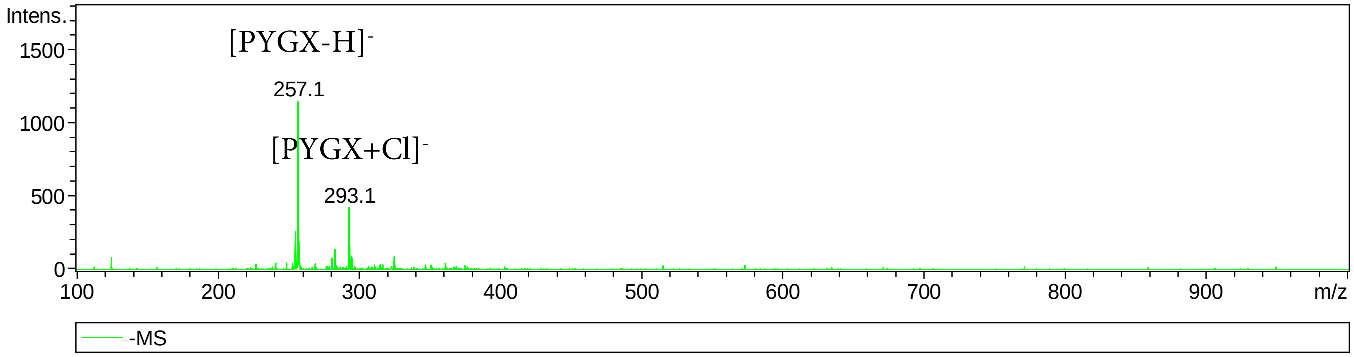

#	m/z	I
1	255.2	259
2	257.1	1145
3	258.0	139
4	283.3	135
5	293.1	424

s)

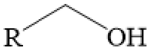

Acceptor: 2,6-Dihydroxynaphtalene

Mass Spectrum List Report

Acquisition Parameter

Ion Source Type	ESI	Ion Polarity	Negative	Alternating Ion Polarity	off
Mass Range Mode	Std/Enhanced	Scan Begin	100 m/z	Scan End	2800 m/z
Capillary Exit	-5.0 Volt	Skimmer	-40.0 Volt	Trap Drive	43.9
Accumulation Time	19759 µs	Averages	5 Spectra	Auto MS/MS	off

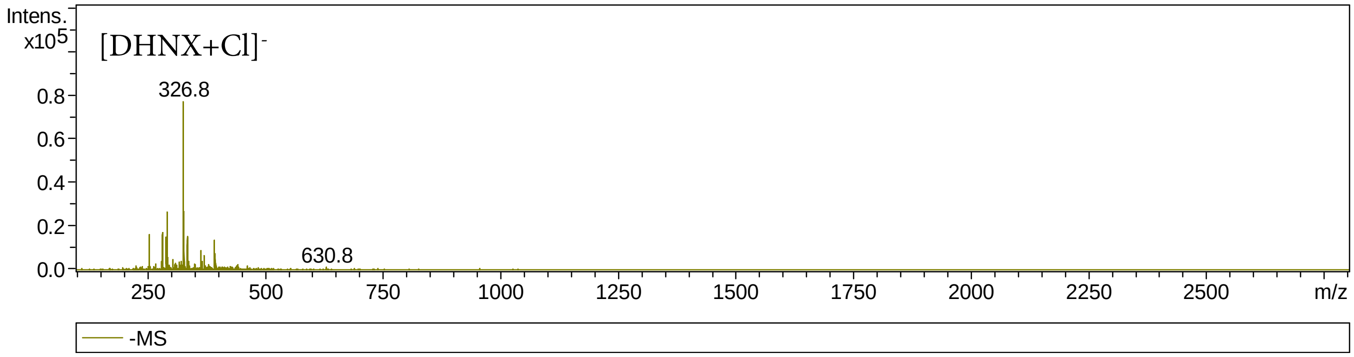

#	m/z	I
1	226.8	1988
2	255.0	16142
3	256.0	2028
4	264.8	1667
5	268.5	2648
6	268.9	1946
7	281.0	3821
8	283.0	17196
9	284.0	2363
10	290.8	15015
11	291.8	1613
12	292.9	26405
13	293.9	5616
14	294.9	1612
15	296.9	2288
16	305.8	4685
17	308.8	2389
18	309.0	2024
19	310.9	2939
20	312.9	2248
21	318.9	3654
22	321.0	2135
23	322.8	3944
24	324.9	2048
25	326.8	77084
26	327.8	13666
27	328.8	26924
28	329.8	3826
29	330.8	1613
30	335.8	15319
31	336.8	6808
32	338.9	3956
33	341.0	1872
34	350.8	2743
35	353.0	2507
36	365.0	8960

#	m/z	I
37	366.0	1875
38	366.9	4043
39	371.8	6449
40	373.8	1758
41	380.8	2470
42	383.0	1673
43	393.0	13743
44	394.0	3270
45	395.0	7553
46	396.9	1905
47	426.9	1606
48	440.6	1777
49	442.6	2519
50	462.8	1840

t)

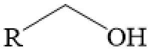

Acceptor: Pterostilbene

Mass Spectrum List Report

Acquisition Parameter

Ion Source Type	ESI	Ion Polarity	Positive	Alternating Ion Polarity	off
Mass Range Mode	Std/Enhanced	Scan Begin	100 m/z	Scan End	1000 m/z
Capillary Exit	5.0 Volt	Skimmer	40.0 Volt	Trap Drive	40.1
Accumulation Time	8034 µs	Averages	7 Spectra	Auto MS/MS	off

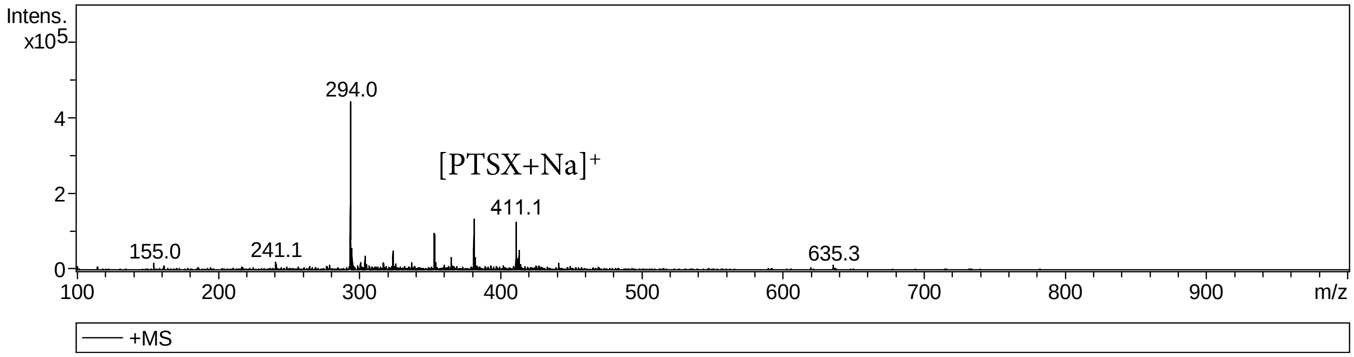

#	m/z	I
1	101.0	8982
2	155.0	17644
3	162.0	9929
4	241.1	21370
5	265.1	8578
6	277.1	9252
7	279.2	12180
8	293.2	8601
9	294.0	443150
10	295.0	56695
11	296.0	10824
12	299.1	11548
13	301.1	19060
14	304.2	35722
15	305.2	14055
16	307.2	10484
17	317.2	19962
18	319.3	8556
19	323.1	9062
20	324.0	48880
21	325.0	9683
22	326.0	16209
23	332.3	9632
24	337.2	19255
25	339.3	8754
26	353.3	96857
27	354.3	18499
28	360.3	13110
29	363.2	9095
30	365.2	32665
31	366.2	9108
32	367.2	8558
33	369.3	8433
34	381.3	133233
35	382.3	32843
36	383.3	11391

#	m/z	I
37	389.2	9211
38	393.3	10463
39	402.3	10024
40	409.2	9082
41	411.1	125850
42	412.2	29277
43	413.3	51063
44	414.3	14969
45	417.3	9224
46	425.3	10404
47	427.2	10027
48	441.3	17445
49	449.4	8410
50	635.3	11638

u)

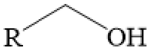

Acceptor: Phenol

Display Report: ZOOM

Acquisition Parameter

Ion Source Type	ESI	Ion Polarity	Positive	Alternating Ion Polarity	off
Mass Range Mode	Std/Enhanced	Scan Begin	100 m/z	Scan End	1200 m/z
Capillary Exit	5.0 Volt	Skimmer	40.0 Volt	Trap Drive	35.0
Accumulation Time	4713 µs	Averages	7 Spectra	Auto MS/MS	off

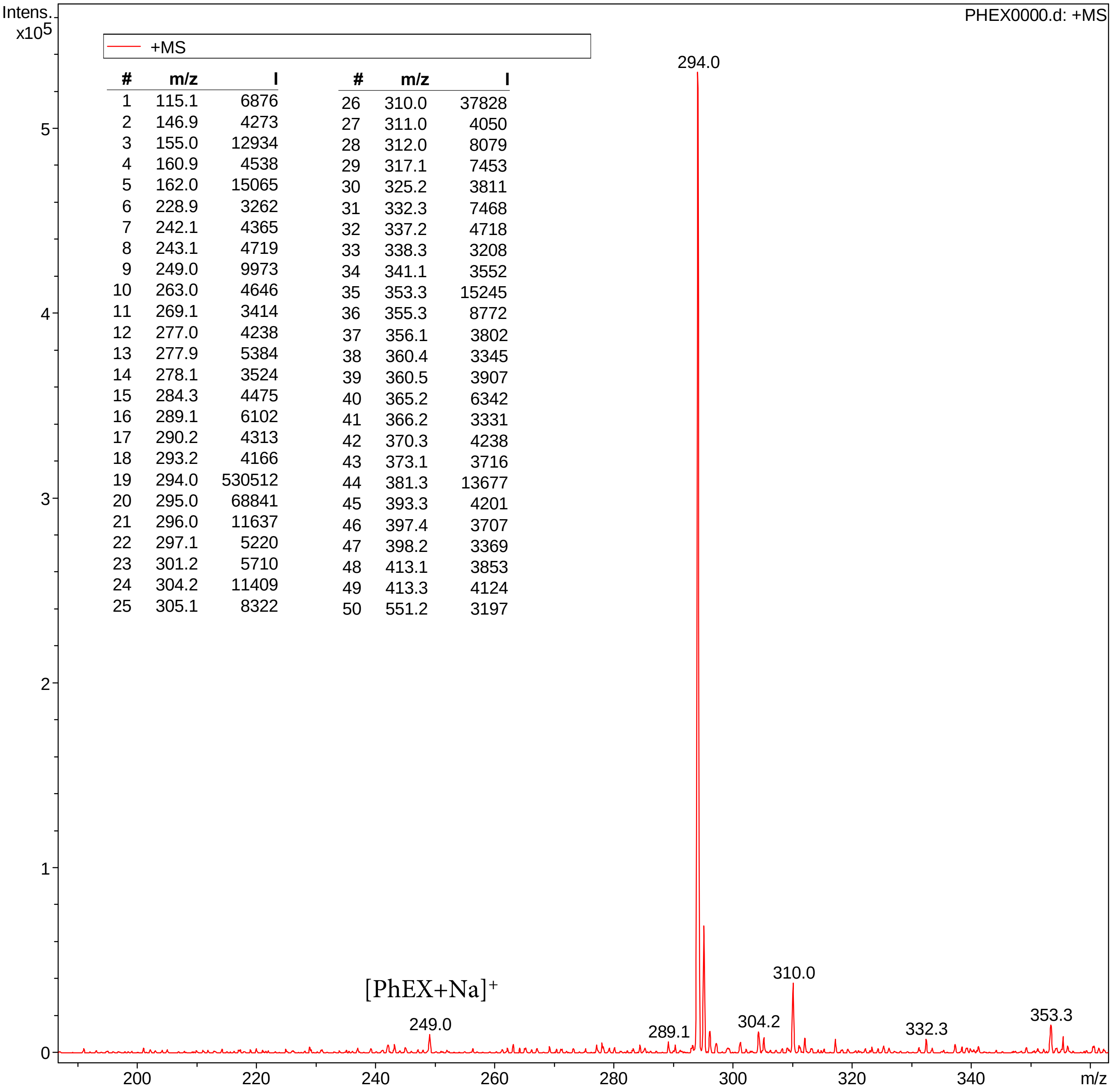

#	m/z	I	#	m/z	I
1	115.1	6876	26	310.0	37828
2	146.9	4273	27	311.0	4050
3	155.0	12934	28	312.0	8079
4	160.9	4538	29	317.1	7453
5	162.0	15065	30	325.2	3811
6	228.9	3262	31	332.3	7468
7	242.1	4365	32	337.2	4718
8	243.1	4719	33	338.3	3208
9	249.0	9973	34	341.1	3552
10	263.0	4646	35	353.3	15245
11	269.1	3414	36	355.3	8772
12	277.0	4238	37	356.1	3802
13	277.9	5384	38	360.4	3345
14	278.1	3524	39	360.5	3907
15	284.3	4475	40	365.2	6342
16	289.1	6102	41	366.2	3331
17	290.2	4313	42	370.3	4238
18	293.2	4166	43	373.1	3716
19	294.0	530512	44	381.3	13677
20	295.0	68841	45	393.3	4201
21	296.0	11637	46	397.4	3707
22	297.1	5220	47	398.2	3369
23	301.2	5710	48	413.1	3853
24	304.2	11409	49	413.3	4124
25	305.1	8322	50	551.2	3197

v)

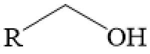

Donor: *p*NPAf Acceptor: Vanillin

Display Report: ZOOM

Acquisition Parameter

Ion Source Type	ESI	Ion Polarity	Positive	Alternating Ion Polarity	off
Mass Range Mode	Std/Enhanced	Scan Begin	50 m/z	Scan End	1200 m/z
Capillary Exit	1.0 Volt	Skimmer	40.0 Volt	Trap Drive	30.0
Accumulation Time	4177 µs	Averages	9 Spectra	Auto MS/MS	off

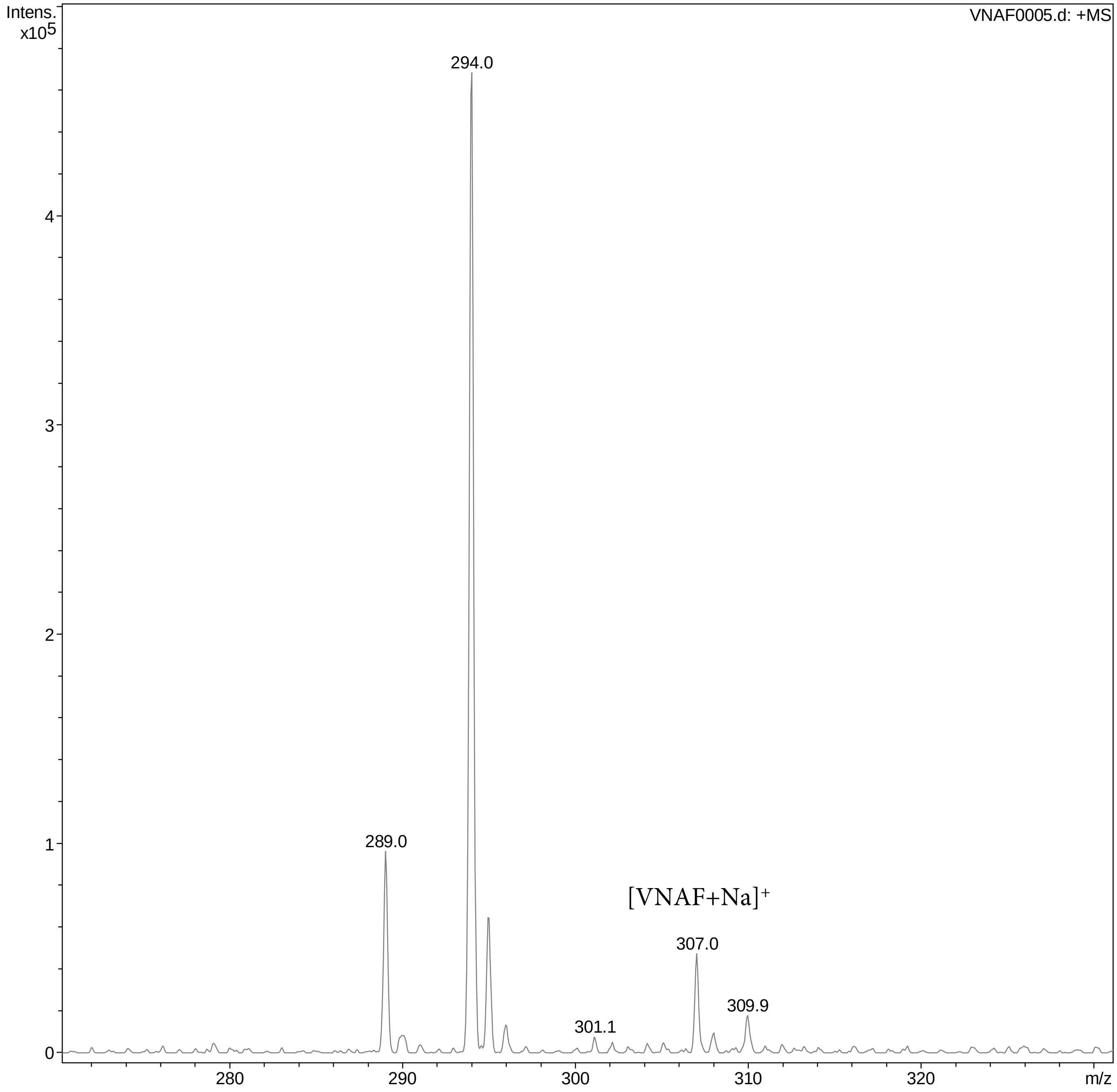

Bruker Daltonics DataAnalysis 3.4

Mass Spectrum List Report

+MS

#	m/z	I
1	200.1	3121
2	213.0	2140
3	214.0	2025
4	225.5	2323
5	236.0	2122
6	240.8	2349
7	252.8	2524
8	272.0	2502
9	274.1	2187
10	276.1	3330
11	278.0	2014
12	279.1	4586
13	280.0	2329
14	280.9	1950
15	281.1	2158
16	283.0	2660
17	289.0	96365
18	290.0	8414
19	291.0	3760
20	292.1	1904
21	292.9	2258
22	294.0	468381
23	294.5	3401
24	295.0	64666
25	296.0	13406
26	297.1	3136
27	300.1	2276
28	301.1	7624
29	302.1	5197
30	303.0	2828
31	304.1	4688
32	305.1	4883
33	305.3	1895
34	306.4	1986
35	307.0	47367
36	308.0	9528
37	309.0	2054
38	309.3	2536
39	309.9	17832
40	311.0	3343
41	311.9	4018
42	312.6	2313
43	313.2	3067
44	314.0	2562
45	316.1	3189
46	317.1	2007
47	319.2	3315
48	322.9	2737
49	324.2	2229
50	325.0	3014
51	325.9	3454
52	327.1	1968
53	330.1	2920
54	331.3	2218
55	333.9	3231
56	335.9	2026
57	337.1	3048
58	338.1	3484
59	339.0	3653
60	340.5	3276
61	341.2	2247
62	342.1	2110
63	344.1	2370
64	345.2	2475
65	346.9	1954
66	348.0	2612
67	349.2	3926
68	350.2	3251
69	351.0	1952
70	351.2	2898
71	352.0	9088
72	353.0	3905
73	353.3	7489
74	354.0	5951
75	355.1	4846
76	358.1	3293
77	359.3	4029
78	361.9	2921
79	362.0	2877
80	365.0	7669
81	369.1	4431
82	369.9	7556
83	372.0	2051
84	374.0	3163
85	381.2	3588
86	382.2	2482
87	383.1	2032
88	384.0	2037
89	391.2	1914
90	392.8	2168
91	407.1	2263
92	413.3	1956
93	414.2	1943
94	415.9	2210
95	424.1	1936
96	427.4	1916
97	450.3	2304
98	507.4	2399
99	565.1	3352
100	619.1	3028

Supplementary Figure 6. ESI-MS spectra of potential glycoconjugates from acids and phenolic acceptors. The clearest spectrum among positive or negative mode is shown for the potential glycoconjugate derived from each tested acceptor: L-glutamic acid (a), N-acetyl-L-glutamic acid (b), 5-aminolevulinic acid (c), acetic acid (d), propionic acid (e), butyric acid (f), nicotinic acid (g), pentanoic acid (h), octanoic acid (i), cholic acid (j), gallic acid (k), caffeic acid (l), sinapic acid (m), quercetin (n), silibinin (o), phloretin (p), methyl 4-hydroxybenzoate (q), pyrogallol (r), 2,6-dihydroxynaphtalene (s), pterostilbene (t), phenol (u), vanillin (v). The main product adducts are labelled. ACEX: xylosyl derivative of acetic acid; AVAX: xylosyl derivative of 5-aminolevulinic acid; BUTX: xylosyl derivative of butyric acid; CAFX: xylosyl derivative of caffeic acid; $CAFX_2$: dixylosyl derivative of caffeic acid; COLX: xylosyl derivative of cholic acid; DHNX: xylosyl derivative of 2,6-dihydroxynaphthalene; GALX: xylosyl derivative of gallic acid; $GALX_2$: dixylosyl derivative of gallic acid; GLUX: xylosyl derivative of L-glutamic acid; M4BX: xylosyl derivative of methyl 4-hydroxybenzoate; NCTX: xylosyl derivative of nicotinic acid; NGAX: xylosyl derivative of N-acetyl-L-glutamic acid; OCTX: xylosyl derivative of octanoic acid; PhEX: xylosyl derivative of phenol; PhLX: xylosyl derivative of phloretin; PNTX: xylosyl derivative of pentanoic acid; PRPX: xylosyl derivative of propionic acid; PTSX: xylosyl derivative of pterostilbene; PYGX: xylosyl derivative of pyrogallol; QUERX: xylosyl derivative of quercetin; $QUERX_2$: dixylosyl derivative of quercetin; SILX: xylosyl derivative of silibinin; SNPX: xylosyl derivative of sinapic acid; $SNPX_2$: dixylosyl derivative of sinapic acid; VANAF: arabinofuranosyl derivative of vanillin.

a)

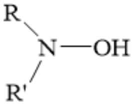

Acceptor: 6-Cl-1-Hydroxybenzotriazole

Mass Spectrum List Report

Acquisition Parameter

Ion Source Type	ESI	Ion Polarity	Positive	Alternating Ion Polarity	off
Mass Range Mode	Std/Enhanced	Scan Begin	100 m/z	Scan End	1000 m/z
Capillary Exit	93.3 Volt	Skimmer	40.0 Volt	Trap Drive	35.1
Accumulation Time	1222 µs	Averages	7 Spectra	Auto MS/MS	off

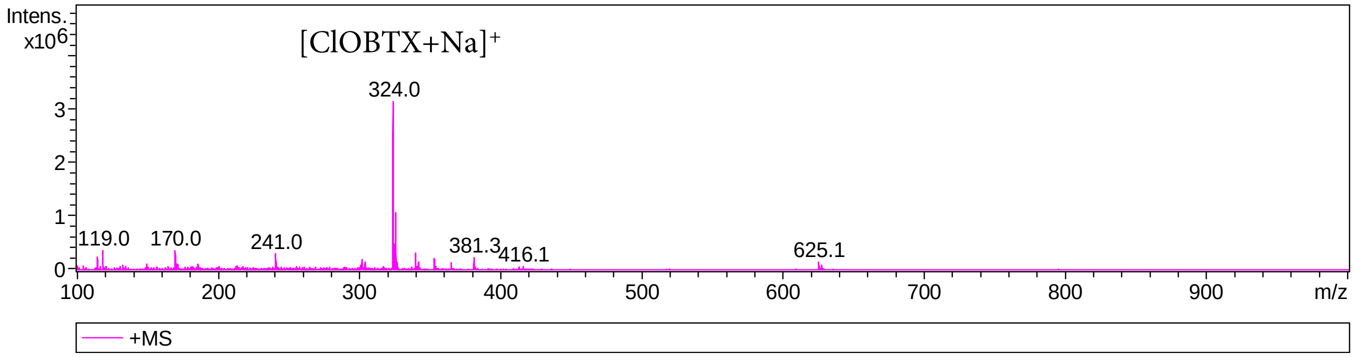

#	m/z	I	#	m/z	I
1	101.0	71549	37	327.0	147876
2	105.0	79499	38	337.1	53741
3	115.0	245812	39	340.0	319573
4	117.0	65650	40	341.0	67183
5	119.0	368856	41	342.0	142004
6	121.0	63089	42	353.2	224218
7	131.0	59696	43	354.2	59244
8	133.0	93560	44	365.1	138853
9	135.0	62771	45	381.3	230986
10	149.9	108433	46	382.2	57327
11	157.0	55753	47	413.2	53816
12	165.0	64131	48	416.1	61866
13	170.0	360960	49	625.1	143432
14	171.0	110991	50	627.1	86797
15	172.0	94424			
16	177.0	53729			
17	179.0	56940			
18	181.9	59134			
19	183.0	53743			
20	186.2	111133			
21	201.0	67142			
22	213.1	64040			
23	214.1	71723			
24	217.9	59267			
25	241.0	298459			
26	242.0	61547			
27	255.9	69093			
28	261.1	51867			
29	279.1	57881			
30	301.1	93341			
31	302.0	200434			
32	304.2	147660			
33	317.2	67391			
34	324.0	3140111			
35	325.0	476721			
36	326.0	1065177			

b)

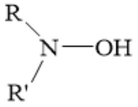

Acceptor: Violuric acid

Mass Spectrum List Report

Acquisition Parameter

Ion Source Type	ESI	Ion Polarity	Negative	Alternating Ion Polarity	off
Mass Range Mode	Std/Enhanced	Scan Begin	100 m/z	Scan End	1000 m/z
Capillary Exit	-93.2 Volt	Skimmer	-40.0 Volt	Trap Drive	43.2
Accumulation Time	3268 µs	Averages	8 Spectra	Auto MS/MS	off

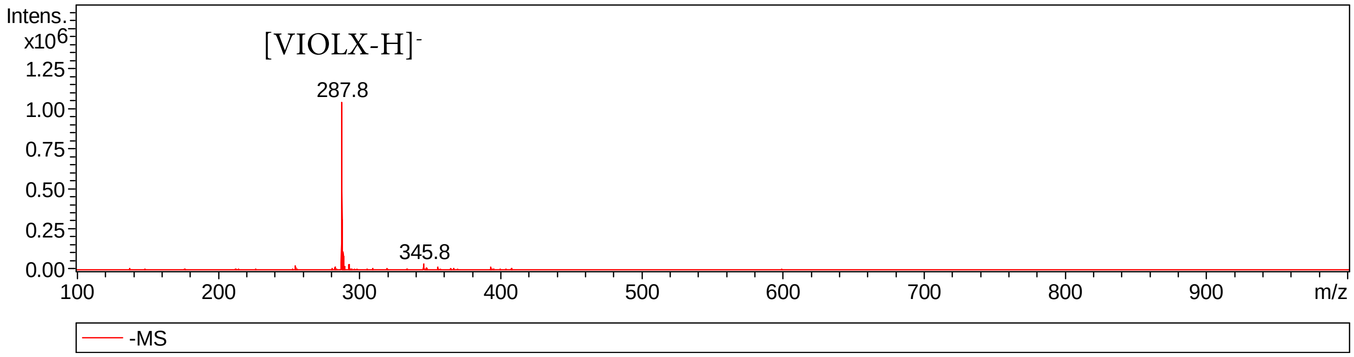

#	m/z	I	#	m/z	I
1	130.8	3186	37	353.0	2885
2	137.7	9475	38	355.8	15597
3	148.8	3478	39	357.7	4035
4	176.8	4339	40	365.0	7528
5	212.8	6859	41	367.0	7450
6	214.8	4162	42	369.8	4831
7	226.9	4013	43	393.0	16350
8	235.6	2798	44	394.0	4827
9	238.9	2946	45	395.0	6070
10	253.0	3663	46	399.8	4317
11	255.0	25648	47	403.7	3593
12	256.0	4749	48	407.8	9128
13	269.0	3281	49	599.0	6890
14	281.0	7461	50	910.0	2734
15	283.1	17344			
16	284.0	6574			
17	287.8	1040429			
18	288.8	114721			
19	289.8	20137			
20	290.7	3359			
21	292.9	33904			
22	293.9	4275			
23	294.8	3601			
24	296.9	5506			
25	298.8	3611			
26	305.8	4525			
27	309.8	7913			
28	319.8	10920			
29	328.8	3170			
30	329.0	2849			
31	333.9	6571			
32	345.8	38759			
33	346.8	5617			
34	347.8	14729			
35	351.0	2785			
36	352.8	2822			

c)

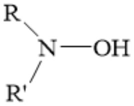

Acceptor: *N*-Hydroxyphthalimide

Mass Spectrum List Report

Acquisition Parameter

Ion Source Type	ESI	Ion Polarity	Positive	Alternating Ion Polarity	off
Mass Range Mode	Std/Enhanced	Scan Begin	100 m/z	Scan End	2200 m/z
Capillary Exit	93.2 Volt	Skimmer	40.0 Volt	Trap Drive	44.3
Accumulation Time	3575 µs	Averages	14 Spectra	Auto MS/MS	off

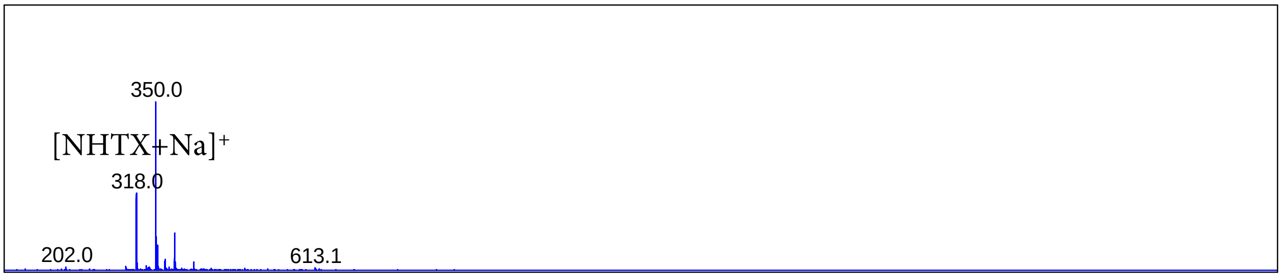

+MS

#	m/z	I
1	135.0	4400
2	194.9	4359
3	202.0	10179
4	241.0	5361
5	249.0	3326
6	301.1	12309
7	318.0	233872
8	319.0	27184
9	320.0	5771
10	325.1	3633
11	333.9	13972
12	335.0	3617
13	336.0	3986
14	337.2	7645
15	339.1	4480
16	339.9	10431
17	341.0	4086
18	350.0	509202
19	351.0	100757
20	352.0	15376
21	353.2	76114
22	354.2	13110
23	355.2	6030
24	358.0	4392
25	365.2	28558
26	366.0	32913
27	367.1	5693
28	368.0	6208
29	372.0	10665
30	375.9	4384
31	381.2	112321
32	382.2	25662
33	383.2	6630
34	385.2	3892
35	393.3	6508
36	397.1	4125
37	409.3	4606
38	413.2	25903
39	414.3	4487
40	425.2	4291
41	428.3	4880
42	430.0	5471
43	433.3	3577
44	441.2	6278
45	449.3	3564
46	489.3	3595
47	497.0	5855
48	535.4	4692
49	613.1	8790
50	619.5	4977

d)

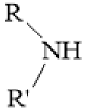

Acceptor: 5-Trifluoromethyl-2*H*-tetrazole

Mass Spectrum List Report

Acquisition Parameter

Ion Source Type	ESI	Ion Polarity	Positive	Alternating Ion Polarity	off
Mass Range Mode	Std/Enhanced	Scan Begin	50 m/z	Scan End	1200 m/z
Capillary Exit	2.0 Volt	Skimmer	40.0 Volt	Trap Drive	30.0
Accumulation Time	9410 µs	Averages	8 Spectra	Auto MS/MS	off

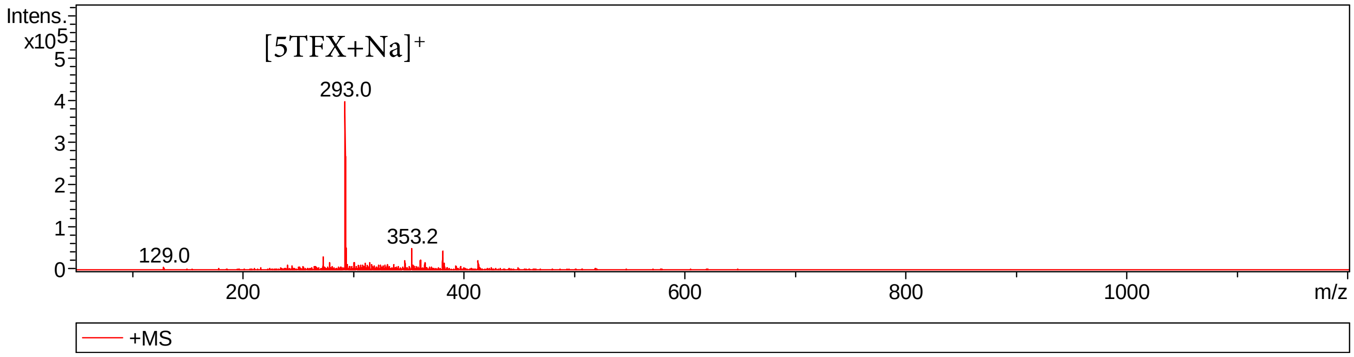

#	m/z	I	#	m/z	I	#	m/z	I
1	129.0	6694	37	302.1	4331	73	354.2	11071
2	217.0	4555	38	303.1	8267	74	355.2	10054
3	234.9	4582	39	304.2	4264	75	357.2	8738
4	238.0	3799	40	305.1	11311	76	359.1	6333
5	241.0	11568	41	306.1	5726	77	361.0	23138
6	245.0	8932	42	307.2	11259	78	363.2	4993
7	245.9	3993	43	308.1	5441	79	365.1	16763
8	251.1	6703	44	309.1	11567	80	366.2	4924
9	252.1	7020	45	310.1	5615	81	367.1	4615
10	255.0	8814	46	311.1	16230	82	369.1	6381
11	259.0	4242	47	312.2	4748	83	371.1	5851
12	262.9	4422	48	313.1	10947	84	372.1	4077
13	265.1	7521	49	314.2	3812	85	377.2	4430
14	266.1	7536	50	315.1	17098	86	381.2	44290
15	267.0	5834	51	317.1	12130	87	382.2	15020
16	267.1	5673	52	318.1	5968	88	383.2	5932
17	269.0	5033	53	319.1	9996	89	385.1	5811
18	271.1	4198	54	321.2	5950	90	393.2	9117
19	273.1	30046	55	323.1	10906	91	394.3	3846
20	274.1	7113	56	325.1	11370	92	397.2	7386
21	275.0	5307	57	327.2	10171	93	400.3	4651
22	277.1	6129	58	329.1	11532	94	407.0	4295
23	279.1	17162	59	331.2	13369	95	413.2	21099
24	280.2	4772	60	333.1	8230	96	414.2	6112
25	281.1	7762	61	335.1	4496	97	415.0	4457
26	283.0	5160	62	337.1	12852	98	425.2	4733
27	285.2	4052	63	338.1	5150	99	441.1	3969
28	287.0	6532	64	339.2	6769	100	449.3	5471
29	289.0	6827	65	341.1	8111			
30	291.0	5747	66	343.1	4695			
31	293.0	396767	67	345.1	6812			
32	294.0	52096	68	347.1	22178			
33	295.0	13204	69	348.1	4459			
34	297.1	7824	70	349.1	5191			
35	299.1	7942	71	351.0	7365			
36	301.1	16765	72	353.2	50040			

e)

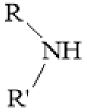

Acceptor: 4-Phenylurazole

Mass Spectrum List Report

Acquisition Parameter

Ion Source Type	ESI	Ion Polarity	Negative	Alternating Ion Polarity	off
Mass Range Mode	Std/Enhanced	Scan Begin	50 m/z	Scan End	1200 m/z
Capillary Exit	-1.0 Volt	Skimmer	-40.0 Volt	Trap Drive	25.0
Accumulation Time	9275 µs	Averages	9 Spectra	Auto MS/MS	off

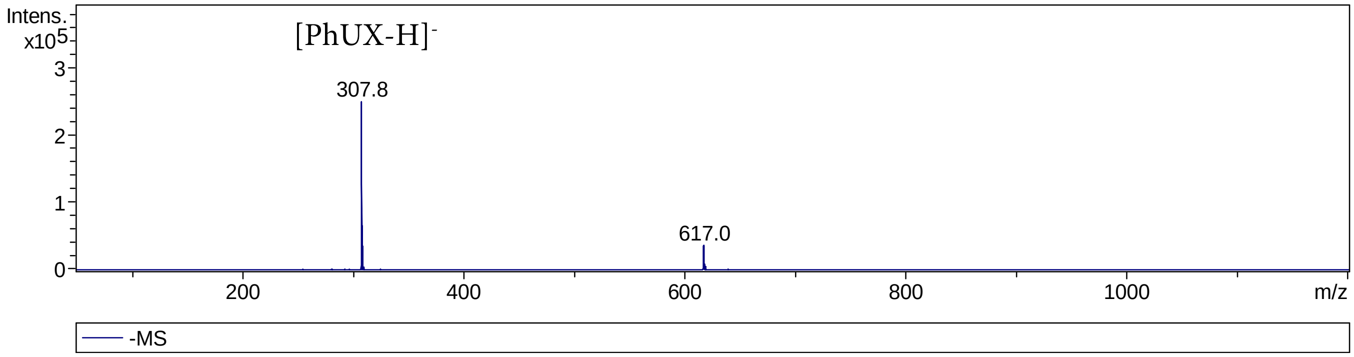

#	m/z	I
1	253.0	361
2	255.0	1368
3	265.0	329
4	279.1	282
5	281.0	1052
6	283.0	493
7	292.9	1072
8	295.0	260
9	297.0	1606
10	298.0	553
11	307.0	287
12	307.8	249414
13	308.8	35335
14	309.8	4183
15	310.7	486
16	310.9	802
17	320.9	342
18	325.0	1063
19	329.0	792
20	339.9	320
21	340.9	253
22	343.8	473
23	370.9	364
24	397.9	360
25	405.8	294
26	550.2	383
27	554.0	356
28	615.8	330
29	616.1	662
30	617.0	36092
31	618.0	9214
32	619.1	4936
33	620.0	583
34	620.9	293
35	639.1	1157
36	679.0	320
37	949.1	323

f)

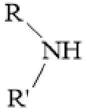

Acceptor: 3-Nitro-1*H*-1,2,4-triazole

Mass Spectrum List Report

Acquisition Parameter

Ion Source Type	ESI	Ion Polarity	Positive	Alternating Ion Polarity	off
Mass Range Mode	Std/Enhanced	Scan Begin	50 m/z	Scan End	1200 m/z
Capillary Exit	1.0 Volt	Skimmer	40.0 Volt	Trap Drive	25.0
Accumulation Time	9681 µs	Averages	9 Spectra	Auto MS/MS	off

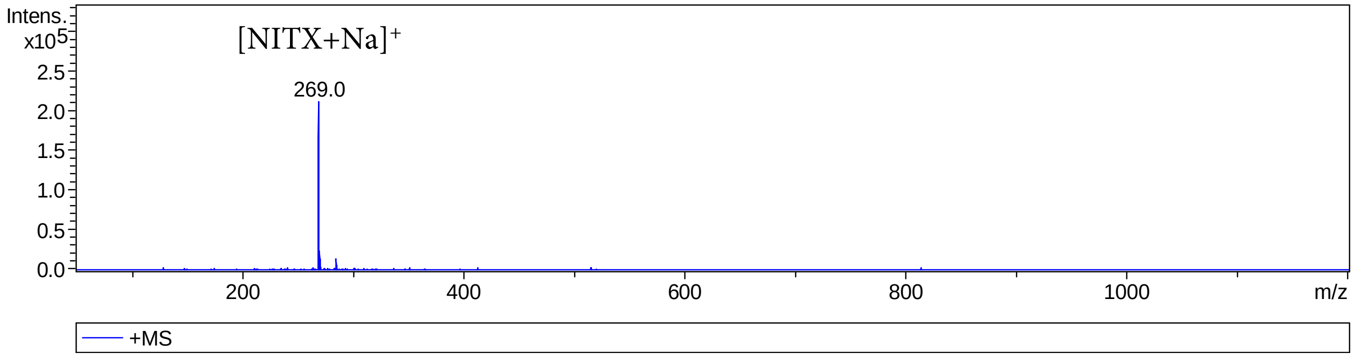

#	m/z	I
1	100.0	584
2	129.0	2409
3	147.9	2243
4	150.0	1318
5	172.0	1220
6	174.9	1562
7	183.0	594
8	188.9	565
9	195.0	1203
10	207.0	538
11	211.0	1600
12	212.8	802
13	213.9	1172
14	217.0	597
15	223.1	601
16	225.1	1132
17	227.1	1013
18	228.0	779
19	228.8	1069
20	235.0	1635
21	238.0	788
22	239.0	1350
23	240.9	2688
24	245.0	617
25	247.0	1289
26	249.0	631
27	253.0	700
28	255.0	655
29	256.1	905
30	263.0	711
31	264.1	3024
32	265.0	829
33	266.1	768
34	269.0	211563
35	269.9	23324
36	271.0	3190
37	273.0	656
38	274.2	2071
39	275.1	913
40	277.2	1518
41	279.1	1390
42	280.9	597
43	283.3	1830
44	284.2	1409
45	284.9	13888
46	285.9	1106
47	286.9	689
48	287.8	720
49	290.1	823
50	291.0	1184
51	293.0	495
52	293.3	1665
53	295.1	706
54	297.1	592
55	298.3	586
56	301.0	2008
57	302.1	2169
58	305.1	715
59	308.0	525
60	309.0	547
61	310.0	1524
62	311.1	531
63	313.1	1132
64	316.2	592
65	317.1	802
66	318.2	806
67	319.2	497
68	320.5	900
69	321.2	785
70	326.3	607
71	328.9	492
72	331.1	665
73	336.9	1599
74	341.2	646
75	347.1	1431
76	351.2	2320
77	352.9	525
78	365.0	1452
79	369.0	591
80	373.1	568
81	380.2	521
82	391.0	570
83	392.1	582
84	397.1	762
85	402.4	545
86	413.1	2450
87	418.0	519
88	418.3	569
89	425.0	574
90	429.3	539
91	442.0	600
92	453.0	562
93	515.1	3077
94	520.2	862
95	538.1	673
96	559.3	588
97	620.4	593
98	662.6	606
99	813.3	2424
100	844.8	560

g)

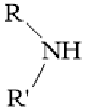

Acceptor: 5-Chlorobenzotriazole

Mass Spectrum List Report

Acquisition Parameter

Ion Source Type	ESI	Ion Polarity	Negative	Alternating Ion Polarity	off
Mass Range Mode	Std/Enhanced	Scan Begin	50 m/z	Scan End	1200 m/z
Capillary Exit	-4.0 Volt	Skimmer	-40.0 Volt	Trap Drive	30.0
Accumulation Time	14158 µs	Averages	9 Spectra	Auto MS/MS	off

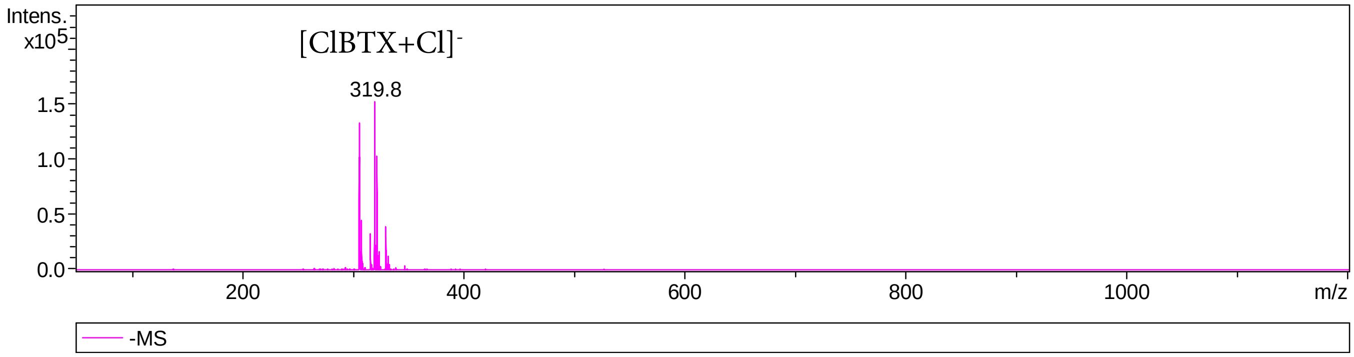

#	m/z	I
1	137.8	983
2	212.9	304
3	227.0	402
4	241.0	322
5	252.9	476
6	255.0	976
7	265.0	1624
8	265.9	620
9	266.9	447
10	269.9	628
11	271.0	596
12	272.5	590
13	273.0	812
14	275.1	365
15	277.0	502
16	279.1	451
17	281.1	575
18	283.1	1280
19	286.6	537
20	289.9	506
21	291.0	1066
22	293.1	1890
23	294.1	355
24	294.8	573
25	296.0	315
26	297.0	639
27	298.1	352
28	299.0	371
29	301.1	671
30	302.1	289
31	305.9	132991
32	306.9	16704
33	307.8	44877
34	308.8	6130
35	309.8	934
36	310.9	2191
37	312.0	319
38	313.0	301
39	314.9	445
40	315.9	32536
41	316.9	4956
42	317.9	1613
43	319.1	322
44	319.8	152251
45	320.8	22036
46	321.8	102556
47	322.8	12981
48	323.7	16497
49	324.8	3320
50	325.9	460
51	327.0	387
52	329.0	526
53	329.8	38772
54	330.8	5553
55	331.8	12669
56	332.8	5121
57	333.9	750
58	334.8	330
59	336.9	674
60	339.0	1795
61	340.0	442
62	340.9	302
63	346.8	3677
64	347.7	422
65	348.8	912
66	350.9	335
67	352.1	283
68	353.0	426
69	353.7	319
70	359.8	498
71	364.9	730
72	366.7	341
73	367.0	688
74	368.9	324
75	369.8	339
76	373.8	356
77	378.8	402
78	381.8	468
79	383.0	325
80	388.8	556
81	389.8	320
82	390.8	371
83	391.8	451
84	392.9	905
85	395.0	398
86	395.7	434
87	397.0	684
88	407.6	451
89	415.8	428
90	417.6	385
91	419.7	605
92	420.7	316
93	425.1	297
94	470.9	294
95	527.2	627
96	568.8	394
97	579.1	498
98	623.1	421
99	823.4	329
100	832.3	368

h) 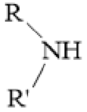

Acceptor: 1,2,4-Triazolo[4,3-a]pyridin-3(2*H*)-one

Mass Spectrum List Report

Acquisition Parameter

Ion Source Type	ESI	Ion Polarity	Positive	Alternating Ion Polarity	off
Mass Range Mode	Std/Enhanced	Scan Begin	50 m/z	Scan End	1200 m/z
Capillary Exit	1.0 Volt	Skimmer	40.0 Volt	Trap Drive	30.0
Accumulation Time	3922 µs	Averages	9 Spectra	Auto MS/MS	off

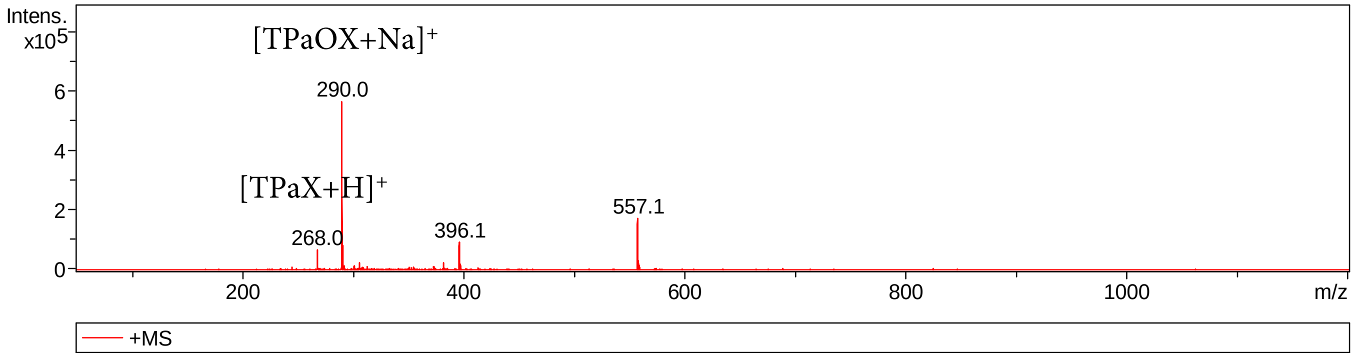

#	m/z	I	#	m/z	I	#	m/z	I
1	178.9	2874	37	319.3	4025	73	407.2	3815
2	213.0	3487	38	323.2	3020	74	413.2	8272
3	223.0	3595	39	324.0	2997	75	415.3	3173
4	234.1	2996	40	325.1	3628	76	424.2	4884
5	234.9	4174	41	327.2	2898	77	430.4	3383
6	241.0	3994	42	330.0	2936	78	440.1	3103
7	244.9	8998	43	331.1	3013	79	441.3	2841
8	249.0	5390	44	332.2	2787	80	452.2	2816
9	268.0	67148	45	333.1	5740	81	452.9	3163
10	269.1	5484	46	335.1	4003	82	457.1	3834
11	270.0	4780	47	337.2	3745	83	496.2	2824
12	273.1	3621	48	339.0	2942	84	513.4	3047
13	274.2	5162	49	341.3	4263	85	534.8	2767
14	279.1	4943	50	343.1	3901	86	535.1	2944
15	284.2	3052	51	345.3	2801	87	536.1	3615
16	287.0	3023	52	347.2	3386	88	557.1	172228
17	290.0	564481	53	349.0	3113	89	558.1	30552
18	291.0	82485	54	350.1	4210	90	559.1	8450
19	292.0	12818	55	351.1	8842	91	573.2	4862
20	295.1	3974	56	353.1	7089	92	574.0	5786
21	299.2	4506	57	355.1	8982	93	577.3	3178
22	301.1	12779	58	358.0	3807	94	597.5	3229
23	302.1	5768	59	365.1	4587	95	607.9	3653
24	303.2	2829	60	373.0	11859	96	634.2	2826
25	304.1	6038	61	374.2	4861	97	664.2	3065
26	305.2	5673	62	375.2	2926	98	688.7	4304
27	306.0	24530	63	381.3	4259	99	824.3	5244
28	307.2	5236	64	382.0	23407	100	1061.5	3257
29	308.0	6945	65	383.1	4048			
30	309.1	8653	66	384.1	2815			
31	310.1	3860	67	385.1	4139			
32	310.4	3652	68	392.3	4770			
33	312.1	3234	69	396.1	93670			
34	313.1	11027	70	397.1	20064			
35	317.2	4406	71	402.1	3598			
36	318.2	3732	72	402.3	4055			

i)

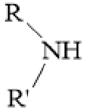

Acceptor: 1*H*-Benzotriazole

Mass Spectrum List Report

Acquisition Parameter

Ion Source Type	ESI	Ion Polarity	Positive	Alternating Ion Polarity	off
Mass Range Mode	Std/Enhanced	Scan Begin	50 m/z	Scan End	1200 m/z
Capillary Exit	4.0 Volt	Skimmer	40.0 Volt	Trap Drive	25.0
Accumulation Time	7688 µs	Averages	9 Spectra	Auto MS/MS	off

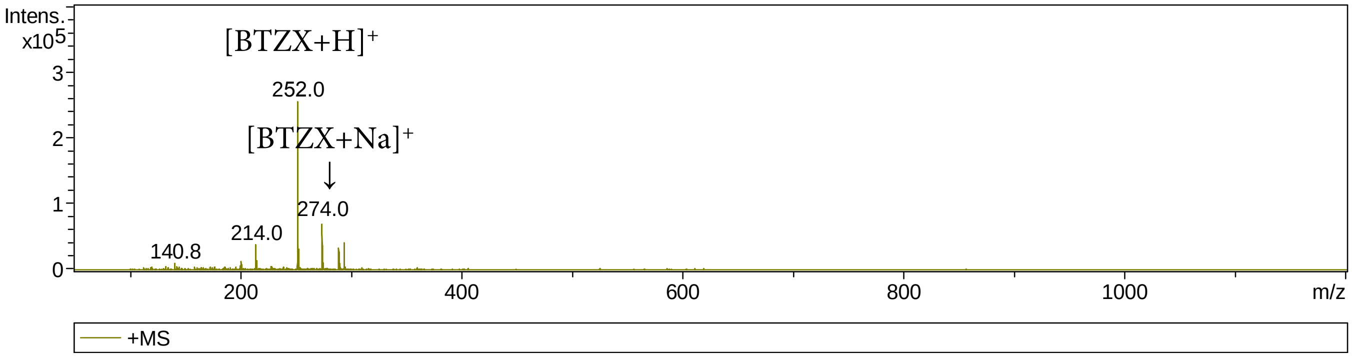

#	m/z	I
1	108.9	1790
2	112.9	2998
3	115.0	1847
4	116.9	1895
5	118.9	3596
6	119.9	4016
7	120.9	2760
8	130.8	1816
9	132.9	5694
10	134.9	3484
11	136.9	1590
12	140.8	9690
13	142.8	5147
14	143.9	1989
15	144.9	4435
16	146.9	2471
17	150.0	2551
18	152.8	1894
19	158.9	4063
20	160.9	3133
21	162.8	2567
22	164.8	3675
23	166.9	3605
24	168.9	2179
25	170.7	1777
26	172.9	4108
27	174.9	3110
28	176.9	4675
29	177.9	1728
30	185.0	2766
31	186.0	4290
32	186.9	1985
33	188.9	1883
34	191.0	3048
35	196.0	4555
36	200.0	5882
37	201.0	13240
38	202.1	2348
39	202.9	2030
40	204.8	1910
41	206.9	1798
42	213.0	2395
43	214.0	38217
44	215.0	13583
45	216.0	1684
46	216.9	2012
47	217.9	2120
48	218.8	1761
49	219.1	1709
50	223.9	1858
51	228.0	5339
52	229.0	3118
53	230.0	2444
54	231.0	2028
55	236.0	2367
56	239.0	4596
57	242.0	3093
58	242.9	2367
59	244.0	2524
60	247.0	1600
61	252.0	255409
62	253.0	31185
63	253.9	3667
64	255.0	2699
65	256.0	1612
66	259.1	1683
67	260.8	2698
68	264.0	2117
69	265.0	2198
70	266.0	1819
71	267.0	2156
72	269.0	2258
73	272.1	2249
74	273.0	1723
75	274.0	69719
76	274.9	11427
77	276.9	1987
78	278.1	1935
79	279.1	2462
80	289.0	33639
81	290.0	10420
82	290.9	1961
83	294.0	41156
84	295.0	3874
85	300.0	1660
86	310.0	3246
87	314.0	1814
88	316.1	2033
89	325.4	1669
90	352.0	1771
91	359.9	3665
92	361.9	1705
93	381.3	1728
94	398.1	1738
95	405.8	1837
96	525.1	2203
97	565.1	1743
98	585.9	1874
99	611.0	2027
100	618.8	2237

j)

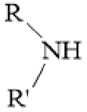

Acceptor: 1*H*-1,2,3-Triazole

Display Report: ZOOM

Acquisition Parameter

Ion Source Type	ESI	Ion Polarity	Negative	Alternating Ion Polarity	off
Mass Range Mode	Std/Enhanced	Scan Begin	50 m/z	Scan End	1200 m/z
Capillary Exit	-3.0 Volt	Skimmer	-40.0 Volt	Trap Drive	35.0
Accumulation Time	200000 µs	Averages	9 Spectra	Auto MS/MS	off

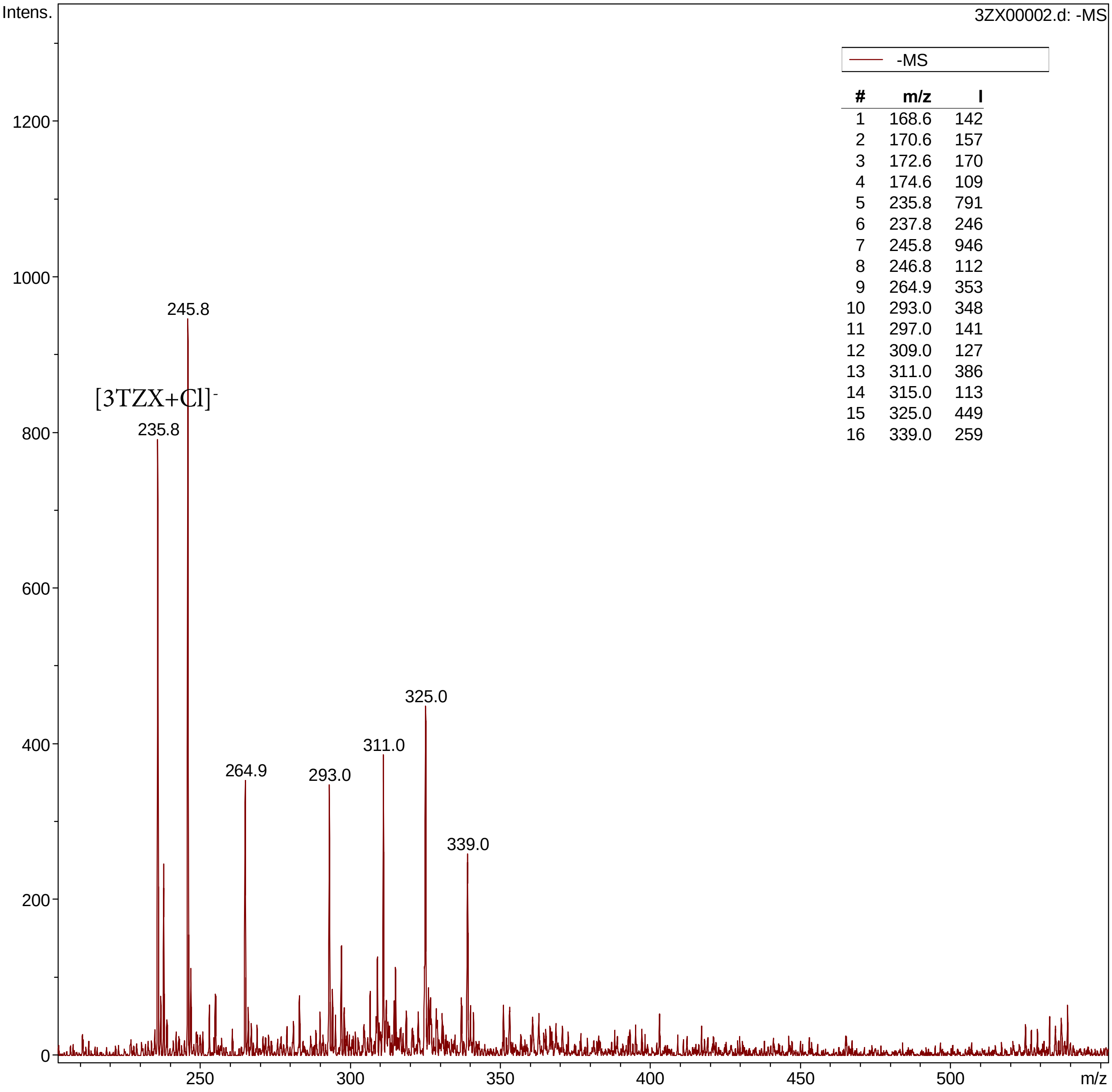

#	m/z	I
1	168.6	142
2	170.6	157
3	172.6	170
4	174.6	109
5	235.8	791
6	237.8	246
7	245.8	946
8	246.8	112
9	264.9	353
10	293.0	348
11	297.0	141
12	309.0	127
13	311.0	386
14	315.0	113
15	325.0	449
16	339.0	259

k)

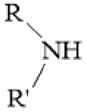

Acceptor: 1*H*-1,2,4-Triazole

Display Report

Acquisition Parameter

Ion Source Type	ESI	Ion Polarity	Negative	Alternating Ion Polarity	off
Mass Range Mode	Std/Enhanced	Scan Begin	50 m/z	Scan End	1200 m/z
Capillary Exit	-3.0 Volt	Skimmer	-40.0 Volt	Trap Drive	35.0
Accumulation Time	200000 µs	Averages	9 Spectra	Auto MS/MS	off

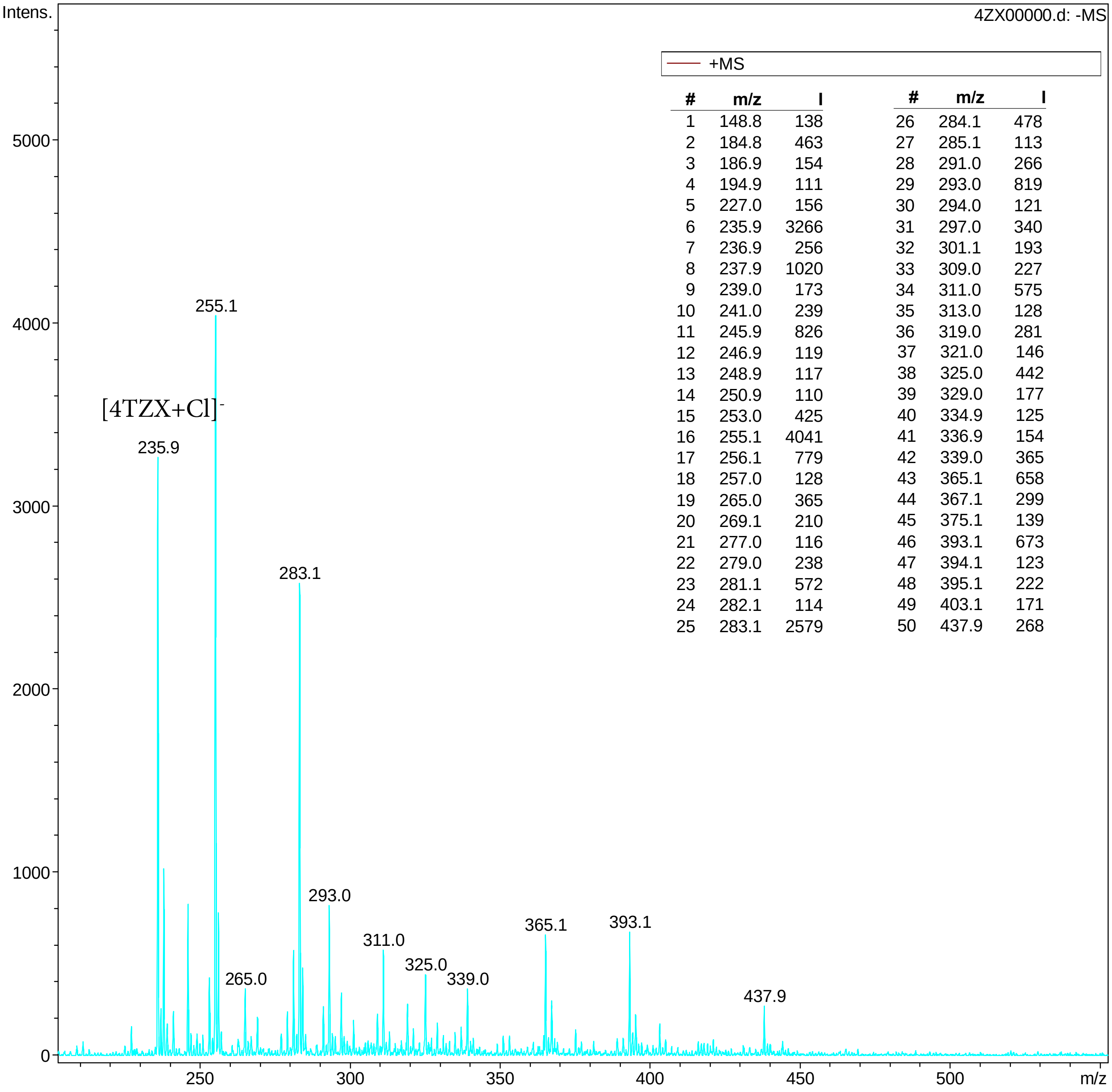

#	m/z	I	#	m/z	I
1	148.8	138	26	284.1	478
2	184.8	463	27	285.1	113
3	186.9	154	28	291.0	266
4	194.9	111	29	293.0	819
5	227.0	156	30	294.0	121
6	235.9	3266	31	297.0	340
7	236.9	256	32	301.1	193
8	237.9	1020	33	309.0	227
9	239.0	173	34	311.0	575
10	241.0	239	35	313.0	128
11	245.9	826	36	319.0	281
12	246.9	119	37	321.0	146
13	248.9	117	38	325.0	442
14	250.9	110	39	329.0	177
15	253.0	425	40	334.9	125
16	255.1	4041	41	336.9	154
17	256.1	779	42	339.0	365
18	257.0	128	43	365.1	658
19	265.0	365	44	367.1	299
20	269.1	210	45	375.1	139
21	277.0	116	46	393.1	673
22	279.0	238	47	394.1	123
23	281.1	572	48	395.1	222
24	282.1	114	49	403.1	171
25	283.1	2579	50	437.9	268

Supplementary Figure 7. ESI-MS spectra of potential glycoconjugates from triazole and tetrazole acceptors. The clearest spectrum among positive or negative mode is shown for the potential glycoconjugate derived from each tested acceptor: 6-Cl-1-hydroxybenzotriazole (a), violuric acid (b), N-hydroxyphthalimide (c), 5-trifluoromethyl-2*H*-tetrazole (d), 4-phenylurazole (e), 3-nitro-1*H*-1,2,4-triazole (f), 5-chlorobenzotriazole (g), 1,2,4-triazolo[4,3-*a*]pyridin-3(2*H*)-one (h), 1*H*-benzotriazole (i), 1*H*-1,2,3-triazole (j), 1*H*-1,2,4-triazole (k). The main product adducts are labelled. 3TZX: Xylosyl derivative of 1*H*-1,2,3-triazole; 4TZX: Xylosyl derivative of 1*H*-1,2,4-triazole; 5TFX: Xylosyl derivative of 5-trifluoromethyl-2*H*-tetrazole; BTZX: Xylosyl derivative of 1*H*-benzotriazole; ClBTX: Xylosyl derivative of 5-chlorobenzotriazole; ClOBTX: xylosyl derivative of 6-chloro-1-hydroxybenzotriazole; NHTX: Xylosyl derivative of *N*-hydroxyphthalimide; NITX: Xylosyl derivative of 3-nitro-1*H*-1,2,4-triazole; PhUX: Xylosyl derivative of phenylurazole; TPaOX: Xylosyl derivative of 1,2,4-triazolo[4,3-*a*]pyridin-3(2*H*)-one; VIOLX: Xylosyl derivative of violuric acid.

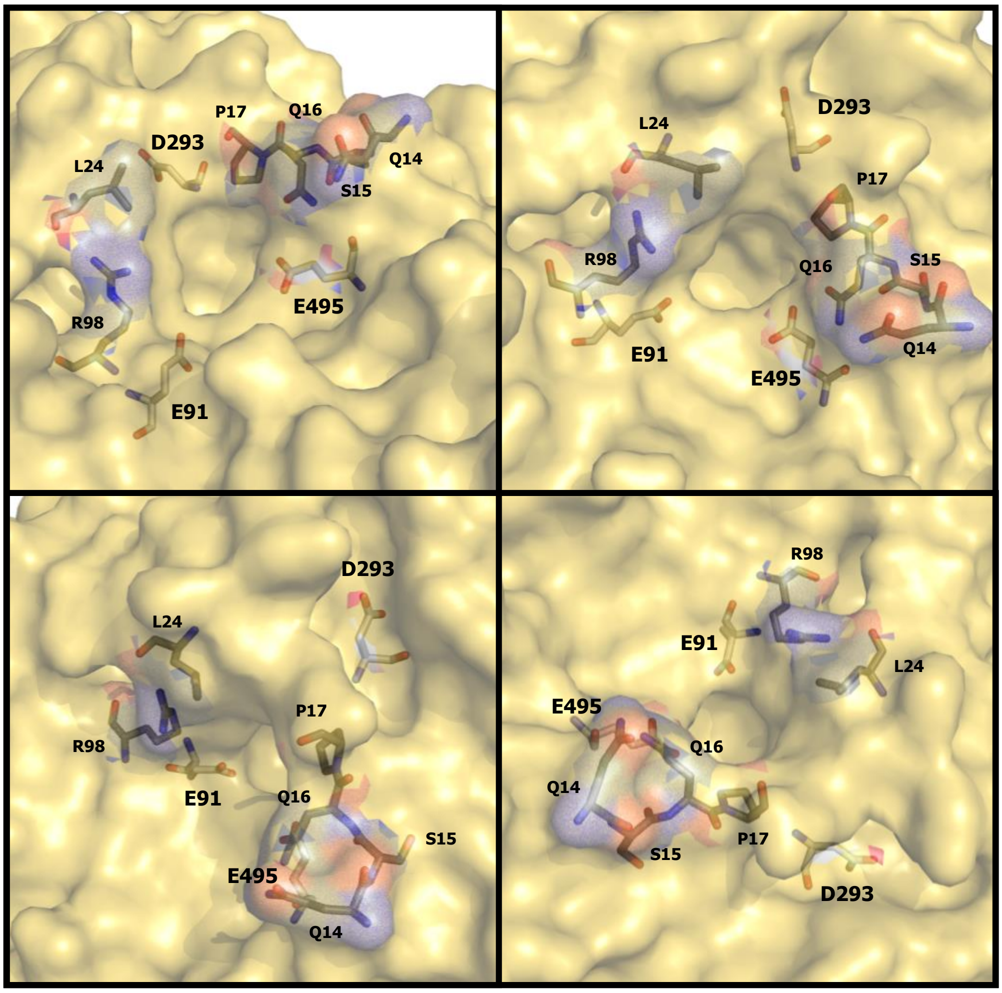

Supplementary Figure 8. Surface representation of rBxTW1-E495A active site. Surface of rBxTW1-E495A active site and the surrounding region is represented from four different perspectives. The catalytic nucleophile (D293), the catalytic acid/base (E495) and the third acid residue (E91) are represented together with some relevant residues (Q14, S15, Q16, P17, L24 and R98) delimiting subsite +1. rBxTW1 model was developed by the SWISS-MODEL tool as described in the Methods section. Images were generated using PyMOL Molecular Graphics System version 1.3.

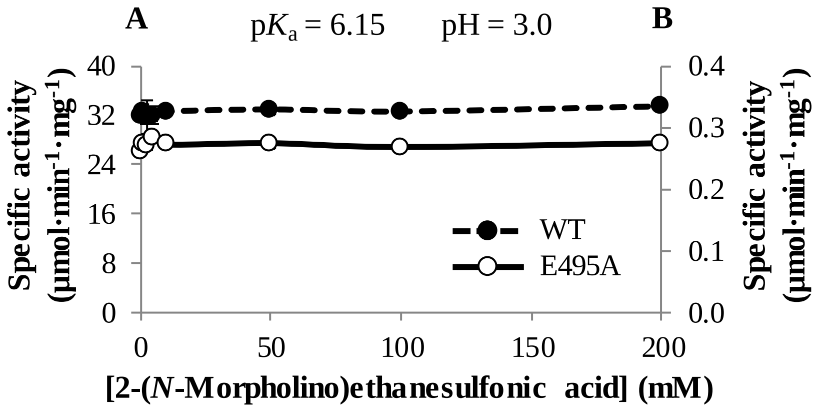

Supplementary Figure 9. Ligase activity response against MES. pK_a of the potential acceptor and reaction pH are indicated, the latter was controlled using 50 mM Gly-HCl buffer. Specific activities were determined spectrophotometrically against *p*NPX. Values for the wild type enzyme (WT) are referred to y-axis-A while y-axis-B corresponds to rBxTW1-E495A. Activity profiles are represented by a dashed line and black circles for the wild type enzyme and by a continuous line and white circles for rBxTW1-E495A. Mean values are shown together with the corresponding standard error (n = 2 independent experiments). Source data are provided as a Source Data file.

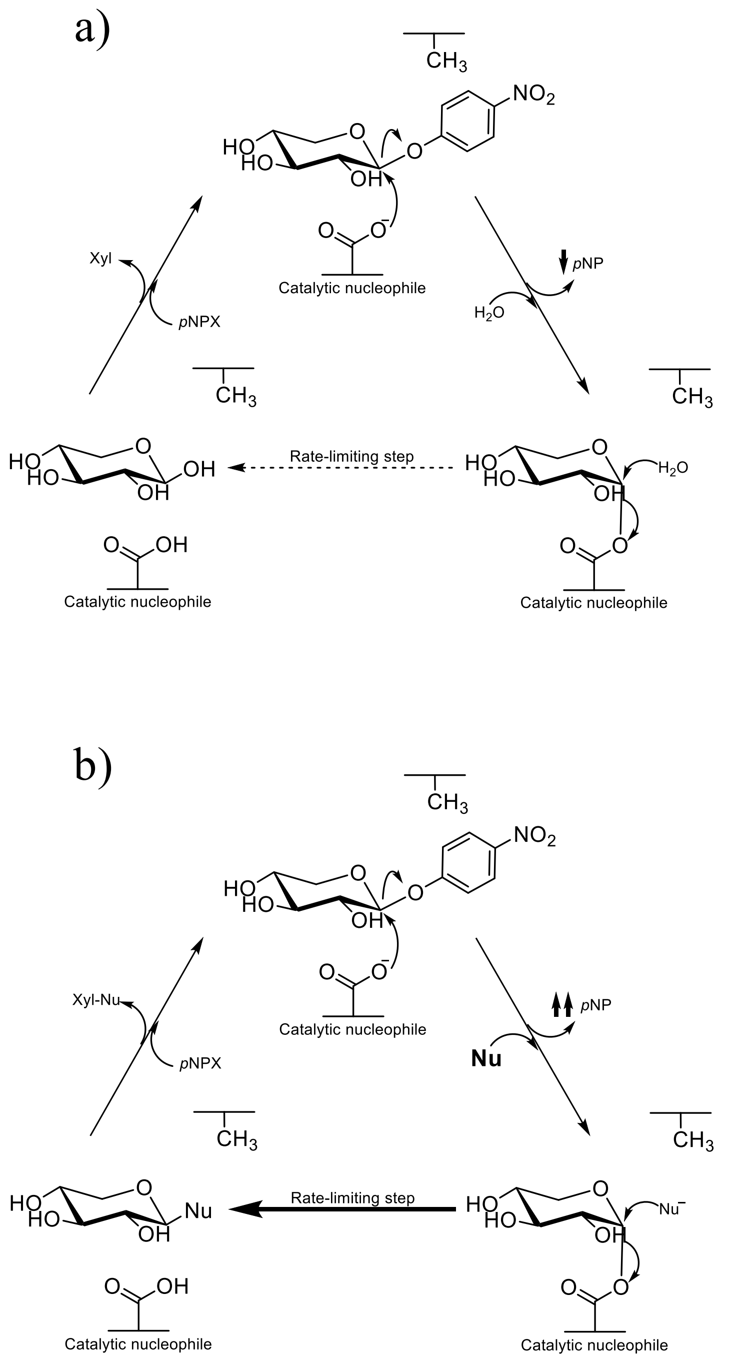

Supplementary Figure 10. Scheme of the rationale of ligase activity response assays. A) In the absence of a positive acceptor, the enzyme is trapped in the form of a glycosylated intermediate, which is hydrolyzed at a slow rate. B) In the presence of an acceptor able to perform a nucleophilic attack on the glycosyl-enzyme intermediate, the regeneration of the free enzyme is accelerated and consequently the cleavage of the *p*NPX donor increases. Nu: Nucleophilic acceptor; Xyl-Nu: Xylosyl derivative of the acceptor.

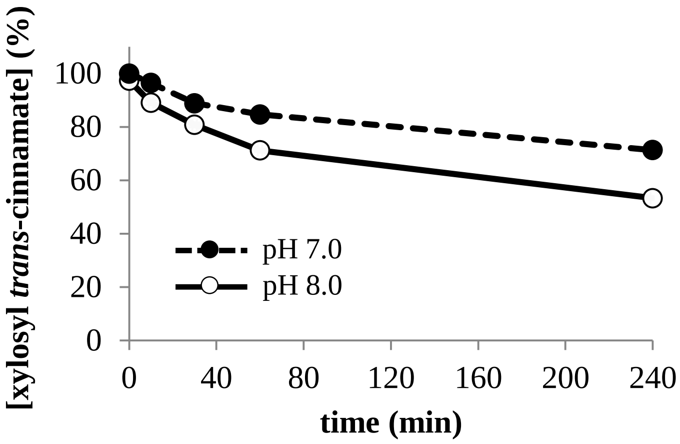

Supplementary Figure 11. Stability of xylosyl *trans*-cinnamate ester. Stability at pH 7 and 8 over time. Mean values are shown together with the corresponding standard error (n = 2 independent experiments). Source data are provided as a Source Data file.

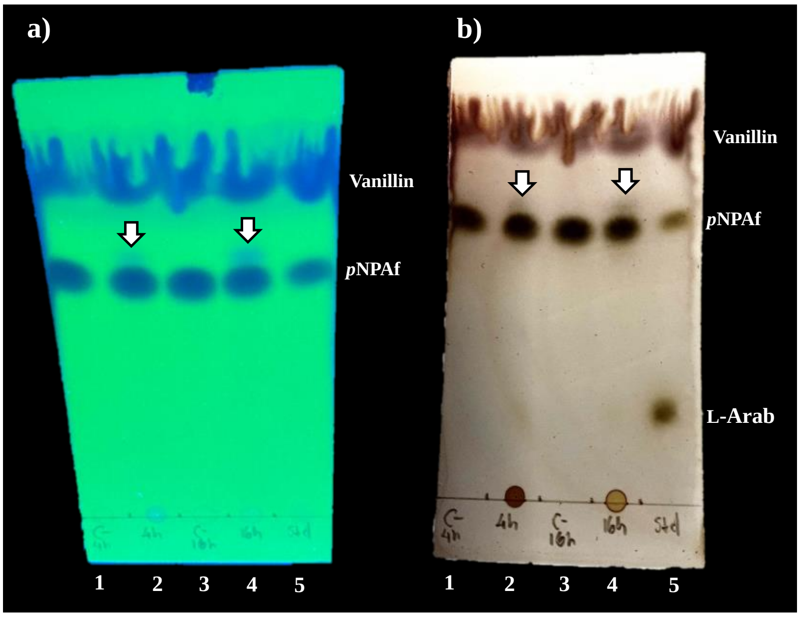

Supplementary Figure 12. Synthesis of vanillyl arabinofuranoside analyzed by TLC. The ability of rBxTW1-E495A to form an arabinofuranoside derivative of vanillin was assayed. Reaction mixtures containing 40 mM *p*NPAf and 25 mM vanillin were analyzed by TLC after 4 and 16 h. Solution C was selected as running buffer. Visualization was performed under 254 nm UV light (a) and using a treatment to reveal sugars and glycoconjugates (b). 1: No enzyme control at 4 h; 2: Complete reaction at 4 h; 3: No enzyme control at 16 h; 4: Complete reaction at 16 h; 5: Mixture of standards containing 30 mM L-arabinose, 15 mM *p*NPAf and 10 mM vanillin. L-Arab: L-Arabinofuranoside; *p*NPAf: 4-Nitrophenyl α-L-arabinofuranoside

SUPPLEMENTARY DISCUSSION

Up to now, the number of reported thioglycoligases has been very low and most of their wild-type counterparts display a narrow range of optimal activity from pH 6 to 8 (Supplementary Table 1). To the best of our knowledge, only the β-hexosaminidase SpHex is active at pH ≤ 3, although its activity becomes residual below 2.5. Therefore, the obtained mutants from rBxTW1 are not only the first reported examples of thioglycoligases from the GH3 family, but also the ones displaying the most acidic pH profile.

The catalytic acid/base glutamic acid residue in rBxTW1 was replaced for either alanine, glycine, or glutamine in order to assess the synthetic abilities of the mutant enzymes. Previously, a characterization of the hydrolytic properties was carried out (Supplementary Table 2), including activity assays in presence of formate and azide[24] (Supplementary Figure 1) for the three obtained mutants. rBxTW1-E495A was selected for going further with the glycosylation assays after showing both the highest hydrolytic k_{cat} and specific activity with the addition of formate and azide[24].

Surprisingly, kinetic determination revealed wide differences in turnover number depending on the selected buffer. Considering the double displacement mechanism, this variance indicated that the second step was the one limiting the reaction rate and pointed towards acetic acid as capable of disrupting the glycosyl-enzyme intermediate efficiently. As expected, a strong decrease in the reaction rate, between 30 to 340 times lower, was noticed for the three mutants when compared to the wild-type rBxTW1, which was reported to show values of k_{cat} and K_m against *p*NPX of 69.3 s^{-1} and 0.20 mM respectively in similar conditions[25].

Once selected, the capacity of rBxTW1-E495A to catalyze the synthesis of *S*-glycosides was tested using thiophenol, as a typical acceptor for thioglycoligases. The

results are shown in Supplementary Figure 2 and revealed the conventional behavior of a thioglycoligase, confirming the formation of the expected product. The assay also indicated that, as expected for *S*-glycosides, the product was not hydrolyzed during the reaction, even after complete consumption of the donor and the addition of fresh enzyme.

The list of acceptors of rBxTW1-E495A studied at the level of conversion yield (Figs. 4 and 5) was expanded by including hydroxycinnamic acids, a class of aromatic acids with the peculiarity of possessing phenolic hydroxyl groups in addition to the carboxylic acid. Because of this, the role as acceptors of *p*-coumaric and ferulic acids was analyzed in more detail by response surface methodology. This approach was also performed to assess the feasibility of addressing the synthesis towards one certain product among the three potential ones. Specific equations were determined from the experimental matrixes (Supplementary Tables 5 and 6) to model the synthesis of each xyloside (Supplementary Equations 1-6) and used to predict their respective conditions for optimal production. Supplementary Table 3 displays the highest production value in each case (theoretical and experimental), together with the acceptor conversion yields and the reaction conditions.

$$[p\text{–Coumaroyl xyloside 35}] = +13.24371 + 0.063618A - 0.474652B - 0.429097C - 0.115408D - 0.0767965E + 1.448 \cdot 10^{-3}AB - 4.552 \cdot 10^{-3}AC - 2.505 \cdot 10^{-3}AD - 6.865 \cdot 10^{-5}AE + 4.387 \cdot 10^{-3}BC - 4.038 \cdot 10^{-3}BD + 9.2242 \cdot 10^{-4}BE + 2.589 \cdot 10^{-3}CD - 7.4282 \cdot 10^{-4}CE + 1.435 \cdot 10^{-4}DE + 8.12 \cdot 10^{-4}A^2 + 5.348 \cdot 10^{-3}B^2 + 0.01785C^2 + 0.012344D^2 + 2.23187 \cdot 10^{-4}E^2 \quad \textbf{(1)}$$

$$[p\text{–Coumaroyl xyloside 36}] = -42.47449 + 0.361162A + 1.81566B + 2.39444C + 0.4174D + 0.122049E - 4.648 \cdot 10^{-3}AB + 0.026263AC + 6.86 \cdot 10^{-4}AD + 1.2190 \cdot 10^{-3}AE - 0.018562BC + 1.190 \cdot 10^{-3}BD - 1.5571 \cdot 10^{-3}BE + 0.020261CD - 1.1515 \cdot 10^{-3}CE + 3.6972 \cdot 10^{-4}DE - 5.228 \cdot 10^{-3}A^2 - 0.020941B^2 - 0.189492C^2 - 0.023225D^2 - 4.46619 \cdot 10^{-4}E^2 \quad \textbf{(2)}$$

$$[p\text{–Coumaroyl xyloside 37}] = -1.32626 + 0.016618A + 0.060532B + 0.161066C - 0.038399D + 9.386783 \cdot 10^{-3}E - 1.048 \cdot 10^{-3}AB + 4.197 \cdot 10^{-3}AC - 7.74 \cdot 10^{-4}AD + 1.6708 \cdot 10^{-4}AE + 6.41 \cdot 10^{-4}BC + 1.097 \cdot 10^{-3}BD - 2.917 \cdot 10^{-5}BE + 1.438 \cdot 10^{-3}CD - 6.4617 \cdot 10^{-4}CE - 6.422 \cdot 10^{-5}DE + 6.07 \cdot 10^{-4}A^2 - 8.00 \cdot 10^{-4}B^2 - 0.021517C^2 - 4.4 \cdot 10^{-5}D^2 - 4.94828 \cdot 10^{-5}E^2 \quad \textbf{(3)}$$

(4) $$[\text{Feruloyl xyloside 39}] = +9.84326 + 0.090276A - 0.344234B - 0.263390C - 0.036115D - 0.122384E + 1.400 \cdot 10^{-3}AB - 1.919 \cdot 10^{-3}AC - 3.903 \cdot 10^{-3}AD - 1.07 \cdot 10^{-4}AE + 3.189 \cdot 10^{-3}BC + 3.22 \cdot 10^{-4}BD + 6.24 \cdot 10^{-4}BE - 2.892 \cdot 10^{-3}CD - 4.93 \cdot 10^{-4}CE - 5.46 \cdot 10^{-4}DE - 3.08 \cdot 10^{-4}A^2 + 3.352 \cdot 10^{-3}B^2 - 0.045968C^2 + 4.305 \cdot 10^{-3}D^2 + 1.019 \cdot 10^{-3}E^2$$

(5) $$[\text{Feruloyl xyloside 40}] = -40.01301 + 0.449386A + 1.38235B + 3.289C + 1.06373D + 0.222167E - 6.539 \cdot 10^{-3}AB + 0.027684AC + 6.94 \cdot 10^{-4}AD + 9.62 \cdot 10^{-4}AE - 0.015254BC - 0.010410BD - 1.993 \cdot 10^{-3}BE + 5.364 \cdot 10^{-3}CD - 0.010008CE - 0.001378DE - 3.781 \cdot 10^{-3}A^2 - 0.013740B^2 - 0.267678C^2 - 0.028545D^2 - 8.08 \cdot 10^{-4}E^2$$

(6) $$[\text{Feruloyl xyloside 41}] = -7.49027 + 0.042148A + 0.272167B + 0.698395C + 0.103308D + 0.059673E - 1.608 \cdot 10^{-3}AB + 0.011936AC - 1.66 \cdot 10^{-4}AD + 6.06 \cdot 10^{-4}AE - 5.18 \cdot 10^{-4}BC + 9.02 \cdot 10^{-4}BD - 4.60 \cdot 10^{-4}BE - 0.012851CD - 3.276 \cdot 10^{-3}CE - 6.85 \cdot 10^{-4}DE + 1.50 \cdot 10^{-4}A^2 - 2.95 \cdot 10^{-3}B^2 - 0.080998C^2 - 4.991 \cdot 10^{-3}D^2 - 3.78 \cdot 10^{-4}E^2$$

A: [*p*NPX] (mM); B: Temperature (°C); C: [Enzyme] ($g \cdot L^{-1}$); D: [NaOH] (mM); E: time (min)

The models were validated based on the good agreement between predicted and experimental values of production, together with the analysis of variance tests (ANOVA) displayed in Supplementary Table 7. The optimal conditions predicted for the glycosylation of the carboxylic group required a minimal neutralization of the pH, whereas much higher concentration of NaOH was calculated to target the aromatic hydroxyl group. These differences reinforce the hypothesis that the ideal reaction pH should be close, but below the pK_a of the nucleophile, to allow both the approach of the neutral molecule and its deprotonation into the active site. Thus, a low pH benefits the formation of the xylose esters (*p*-coumaric and ferulic acids have pK_1 of 4.39 and 4.56 respectively) while an increase in basicity displaces the catalysis towards the glycosylation of the hydroxyl group (pK_2 of 8.37 and 8.65 for *p*-coumaric and ferulic acids, respectively). In addition, to exclude that potential instability of glycosyl esters would be the cause of the reduced yield at high pH, the stability of xylosyl *trans*-cinnamate was evaluated for 4 hours at pH 7 and 8. *Trans*-cinnamic acid was selected as acceptor for its similarity to *p*-coumaric and ferulic acids, although without hydroxyl groups that would complicate the interpretation of the assay. Supplementary Figure 11

shows that even though certain spontaneous degradation can be observed, it cannot explain the difference in yields attained for the hydroxycinnamic esters depending on NaOH concentration.

The results displayed in Supplementary Table 3 also indicate that the distance between carboxylic and hydroxyl groups, or the presence of the aromatic ring in between, seems to prevent the electrostatic repulsion caused by the negatively charged carboxylate presumably present under more basic conditions. In addition, higher yields were attained for the glycosylation of the hydroxyl group in comparison to the carboxylic acid. This was expected considering that the production of vanillin and EGCG xylosyl derivatives also surpassed the optimum value for *trans*-cinnamic acid, probably due to sugar esters being more susceptible to enzymatic hydrolysis by the thioglycoligase.

SUPPLEMENTARY METHODS

Characterization of mutants from rBxTW1

Kinetics of E495A, E495G and E495Q were evaluated against *p*NPX. The standard reaction was modified to use increasing substrate concentrations from 0.078 to 20 mM. The assay was performed at pH 5.0 both with 50 mM sodium acetate buffer and 50 mM sodium citrate buffer. An additional kinetic study in the same conditions was performed for rBxTW1-E495A against 4-nitrophenyl α-L-arabinopyranoside (*p*NPAp) using substrate concentrations from 0.156 to 40 mM and 50 mM sodium acetate buffer (pH 5.0). The activity data obtained were adjusted to a two-parameter rectangular hyperbola (Michaelis-Menten model) with SigmaPlot® software version 14.0 (Systat Software Inc.).

Ligase activity response assays were performed by adding the appropriate concentration of the mutants and the wild type rBxTW1 to a reaction mixture with 3.5 mM *p*NPX, 50 mM sodium 2-(*N*-morpholino)ethanesulfonate (MES) buffer (pH 5.5) and increasing concentrations of sodium formate from 0 to 5 M or sodium azide from 0 to 2 M. In order to avoid interferences in the assay, the pH of both nucleophile stock solutions was neutralized to 5.5 by adding the appropriate quantity of NaOH or HCl.

As standard thioglycoligase assay, the synthesis of phenyl β-D-thioxylopyranoside (PhTX) was carried out with 0.55 g/L E495A. *p*NPX was added as donor and assayed at 10 and 20 mM whereas thiophenol (PhT) was selected as acceptor and assayed at 40 mM and saturation. Methanol was added as co-solvent at 10 % (v/v). Reactions were incubated at 1,200 rpm and 40 ºC and samples were withdrawn after 0.2, 1, 2, 4 and 24 h to be analyzed by high-performance liquid chromatography (HPLC) as described below. After 24 h a dosage of fresh enzyme was added to the reactions at a final

concentration of 1.10 g/L. Samples were withdrawn and analyzed after additional 12 h to assess the hydrolysis of the *thio*-xyloside.

Production of xylosides from hydroxycinnamic acids

The glycosylation of *p*-coumaric and ferulic acids was studied by response surface methodology. A Central Composite Design (CCD) experimental matrix was generated using the Design-Expert® software version 10.0.1.0 (Stat-Ease Inc.). Concentration of *p*NPX, enzyme and NaOH, together with reaction time and temperature were selected as independent variables. Sodium hydroxide was employed to regulate the initial pH of the reaction. The design space was defined by assessing these parameters at three levels: low, middle, and high, determined by a one-factor-at-a-time method, although CCD also includes star points outside these limits. The concentration of the acceptor was fixed at 25 mM and the productions of each of the three possible glycoconjugates (two mono-xylosides and one di-xyloside) were registered as responses (Supplementary Table 5 and Supplementary Table 6). The software used these data to generate polynomial quadratic equations which integrate the effect of the independent variables on the response and can be used to predict the conditions of the maximal production for the selected xyloside.

The stability of xylosyl hydroxycinnamic esters at basic pH was studied using xylosyl *trans*-cinnamate as a simplified model. This ester was prepared enzymatically using 4 g/L rBxTW1-E495A, 40 mM *p*NPX as donor, 20 mM *trans*-cinnamic acid as acceptor and 50 mM Glycine-HCl (pH 3). Reaction was incubated at 1,200 rpm and 40 ºC for 20 min and stopped by heating at 100 ºC for 5 min. Concentration of xylosyl *trans*-cinnamate was determined by HPLC and considered as the initial value of the stability assay (100%). The stopped reaction was incubated at 40 ºC and pH 7.0 and 8.0 using 100

mM of sodium phosphate buffer. Residual concentration of the xylose ester was determined at 10 min, 30 min, 1 h and 4 h for both pH values.

Characterization of the identified glycoconjugates

2: ^{1}H NMR (600 MHz, D_2O) δ 5.41 (d, J = 7.7 Hz, 1H), 4.13 (dd, J = 8.9, 4.7 Hz, 1H), 3.9 (dd, J = 11.7, 5.4 Hz, 1H), 3.63 – 3.53 (m, 1H), 3.48 – 3.33 (m, 3H), 2.46 (q, J = 7.8 Hz, 2H), 2.14 – 2.07 (m, 1H), 1.94 (s, 3H), 1.93 – 1.87 (m, 1H).

^{13}C NMR (151 MHz, D_2O) δ 177.39, 173.92, 173.92, 94.74, 75.41, 71.7, 68.83, 65.77, 54.32, 30.51, 26.53, 21.61.

ESI-MS m/z: calcd. for $C_{12}H_{19}NO_9$ $[M-H]^-$:320.1, found: 319.9.

4: ^{1}H NMR (600 MHz, D_2O) δ 7.81 (d, J = 16.1 Hz, 1H), 7.62 (dd, J = 7.3, 1.9 Hz, 2H), 7.47 – 7.38 (m, 3H), 6.56 (d, J = 16.0 Hz, 1H), 5.57 – 5.50 (m, 1H), 3.95 (dd, J = 11.6, 5.4 Hz, 1H), 3.63 (ddd, J = 10.4, 8.3, 5.3 Hz, 1H), 3.55 – 3.47 (m, 2H), 3.41 (t, J = 11.0 Hz, 1H).

^{13}C NMR (151 MHz, D_2O) δ 167.14, 148.03, 133.81, 131.28, 129.08, 129.08, 128.55, 128.55, 116.16, 94.85, 75.42, 71.26, 69, 65.79.

ESI-MS m/z: calcd. for $C_{14}H_{16}O_6$ $[M+Na]^+$:303.1, found: 303.0.

6: ^{1}H NMR (600 MHz, D_2O) δ 5.40 (d, J = 7.9 Hz, 1H), 3.90 (dd, J = 11.6, 5.4 Hz, 1H), 3.59 (ddd, J = 10.4, 8.8, 5.4 Hz, 1H), 3.48 – 3.33 (m, 3H), 2.40 (t, J = 7.4 Hz, 2H), 1.84 (s, 1H), 1.56 (p, J = 7.1 Hz, 2H), 1.23 (dq, J = 7.2, 3.8, 3.3 Hz, 4H), 0.82 – 0.76 (m, 3H).

^{13}C NMR (151 MHz, D_2O) δ 175.48, 94.43, 75.26, 71.49, 68.66, 65.67, 33.45, 30.22, 23.27, 21.21, 14.27.

ESI-MS m/z: calcd. for $C_{11}H_{20}O_6$ $[M+Na]^+$:271.1, found: 271.1.

8: ^{1}H NMR (600 MHz, D_2O) δ 5.41 (d, J = 7.9 Hz, 1H), 3.92 (dd, J = 11.6, 5.4 Hz, 1H), 3.59 (td, J = 9.6, 5.4 Hz, 1H), 3.49 – 3.33 (m, 3H), 2.41 (t, J = 7.4 Hz, 2H), 1.56 (p, J = 7.3 Hz, 2H), 1.29 – 1.18 (m, 6H), 0.81 – 0.76 (m, 3H).

^{13}C NMR (151 MHz, D_2O) δ 175.49, 94.87, 75.7, 72.16, 69.26, 65.86, 33.59, 31.42, 28.32, 24.1, 21.29, 11.57.

ESI-MS m/z: calcd. for $C_{12}H_{22}O_6$ $[M+Na]^+$:285.1, found: 284.6.

10: ^{1}H NMR (600 MHz, D_2O) δ 9.72 (s, 1H), 7.55 – 7.48 (m, 2H), 7.23 (d, J = 8.3 Hz, 1H), 5.15 (d, J = 7.6 Hz, 1H), 3.95 (dd, J = 11.6, 5.4 Hz, 1H), 3.86 (s, 3H), 3.70 – 3.55 (m, 2H), 3.51 (t, J = 9.2 Hz, 1H), 3.47 – 3.40 (m, 1H).

^{13}C NMR (151 MHz, D_2O) δ 194.37, 150.7, 149.26, 131.31, 126.6, 115.12, 111.8, 100.21, 75.21, 72.67, 69.17, 65.74, 56.23.

ESI-MS m/z: calcd. for $C_{12}H_{22}O_6$ $[M+Na]^+$:307.1, found: 307.1.

11: ^{1}H NMR (600 MHz, D_2O) δ 9.72 (s, 1H), 7.57 – 7.50 (m, 2H), 7.26 (d, J = 8.3 Hz, 1H), 5.1 (d, J = 7.6 Hz, 1H), 3.98 – 3.78 (m, 7H), 3.74 (dd, J = 9.8, 3.5 Hz, 1H).

^{13}C NMR (151 MHz, D_2O) δ 195.52, 151.09, 149.1, 131.21, 126.74, 115.03, 111.54, 100.28, 72.12, 70.07, 68.03, 66.35, 55.99.

ESI-MS m/z: calcd. for $C_{12}H_{22}O_6$ $[M+Na]^+$:307.1, found: 307.0.

13: ^{1}H NMR (600 MHz, D_2O) δ 6.89 (s, 2H), 6.50 (s, 2H), 6.09 – 6.03 (m, 2H), 5.54 – 5.50 (m, 1H), 5.01 (s, 1H), 4.87 (d, J = 7.7 Hz, 1H), 3.85 (dd, J = 11.7, 5.4 Hz, 1H), 3.67 – 3.56 (m, 1H), 3.51 (dd, J = 9.4, 7.7 Hz, 1H), 3.41 (t, J = 9.2 Hz, 1H), 3.15 (t, J = 11.0 Hz, 1H), 2.98 (dd, J = 17.5, 4.5 Hz, 1H), 2.89 – 2.83 (m, 1H).

^{13}C NMR (151 MHz, D_2O) δ 165.8, 154.95, 148.53, 145.14, 135.99, 132.15, 129.5, 127.06, 109.7, 106.46, 103.8, 99.41, 96.07, 95.3, 77.18, 75.42, 73.33, 69.2, 69.04, 65.54, 25.13.

ESI-MS m/z: calcd. for $C_{27}H_{26}O_{15}$ $[M+Na]^+$:613.1, found: 613.2.

15: ^{1}H NMR (600 MHz, D_2O) δ 7.85 (dd, J = 24.9, 8.6 Hz, 2H), 7.75 (t, J = 7.8 Hz, 1H), 7.58 (dd, J = 8.6, 7.0 Hz, 1H), 5.82 (d, J = 9.1 Hz, 1H), 4.15 – 4.06 (m, 2H), 3.81 (td, J = 9.9, 5.5 Hz, 1H), 3.68 – 3.56 (m, 2H).

^{13}C NMR (151 MHz, D_2O) δ 132.2, 127.39, 114.4, 111.53, 86.8, 76.08, 71.19, 68.66, 68.03.

ESI-MS m/z: calcd. for $C_{11}H_{13}N_3O_5$ $[M+Na]^+$:290.1, found: 290.1.

17: ^{1}H NMR (600 MHz, D_2O) δ 7.58 (d, J = 4.0 Hz, 1H), 7.22 (d, J = 4.0 Hz, 1H), 5.95 (d, J = 9.1 Hz, 1H), 4.15 – 4.06 (m, 2H), 3.80 (ddd, J = 10.7, 9.2, 5.5 Hz, 1H), 3.68 – 3.55 (m, 2H).

^{13}C NMR (151 MHz, D_2O) δ 160.71, 131.53, 129.95, 129.47, 116.02, 89.83, 75.91, 71.8, 68.7, 68.01.

ESI-MS m/z: calcd. for $C_{10}H_{11}BrN_4O_4S$ $[M+Na]^+$:385.0, 387.0, found: 384.9, 386.9.

19: ^{1}H NMR (600 MHz, D_2O) δ 4.60 (d, J = 8.7 Hz, 1H), 3.95 (dd, J = 11.6, 5.5 Hz, 1H), 3.56 (ddd, J = 10.6, 9.1, 5.5 Hz, 1H), 3.41 – 3.28 (m, 2H), 3.17 (t, J = 9.0 Hz, 1H).

^{13}C NMR (151 MHz, D_2O) δ 90.9, 75.8, 72.74, 68.96, 66.9.

ESI-MS m/z: calcd. for $C_5H_9N_3O_4$ $[M+Na]^+$:198.0, found: 198.0.

21: ^{1}H NMR (600 MHz, D_2O) δ 5.46 (d, J = 8.9 Hz, 1H), 4.02 (dd, J = 11.5, 5.5 Hz, 1H), 3.86 (t, J = 9.1 Hz, 1H), 3.69 (ddd, J = 10.6, 9.3, 5.5 Hz, 1H), 3.60 – 3.47 (m, 2H).

^{13}C NMR (151 MHz, D_2O) δ 141.06, 133.28, 86.49, 76.29, 72.16, 68.96, 67.85.

ESI-MS m/z: calcd. for $C_7H_9Br_2N_3O_4$ $[M+Na]^+$:379.9, 381.9, 383.9, found: 379.8, 381.8, 383.8.

23: ^{1}H NMR (600 MHz, D_2O) δ 8.70 (d, J = 4.6 Hz, 1H), 8.33 (d, J = 8.5 Hz, 1H), 7.63 (dd, J = 8.5, 4.5 Hz, 1H), 6.00 (d, J = 9.2 Hz, 1H), 4.20 (t, J = 9.2 Hz, 1H), 4.13 (dd, J = 11.5, 5.6 Hz, 1H), 3.88 (ddd, J = 10.8, 9.2, 5.5 Hz, 1H), 3.72 – 3.59 (m, 2H).

^{13}C NMR (151 MHz, D_2O) δ 155.69, 149.56, 125.68, 123.95, 121.47, 87.66, 76.13, 71.43, 68.76, 68.02.

ESI-MS m/z: calcd. for $C_{10}H_{12}N_4O_4$ $[M+Na]^+$:275.1, found: 275.0.

25: ^{1}H NMR (600 MHz, D_2O) δ 7.68 (dd, J = 12.7, 7.8 Hz, 2H), 7.57 – 7.33 (m, 3H), 4.74 (d, J = 7.9 Hz, 1H), 3.82 – 3.73 (m, 1H), 3.56 – 3.44 (m, 1H), 3.32 (t, J = 9.1 Hz, 1H), 3.27 – 3.12 (m, 2H).

^{13}C NMR (151 MHz, D_2O) δ 130.88, 130.53, 128.23, 97.59, 75.19, 73.47, 69.1, 65.55.

ESI-MS m/z: calcd. for $C_{11}H_{15}O_7P$ $[M-H]^-$:289.0, found: 288.8.

27: ^{1}H NMR (600 MHz, D_2O) δ 4.88 (t, J = 7.5 Hz, 1H), 4.00 (dd, J = 11.7, 5.4 Hz, 1H), 3.76 – 3.62 (m, 1H), 3.51 (t, J = 9.2 Hz, 1H), 3.41 – 3.33 (m, 2H).

^{13}C NMR (151 MHz, D_2O) δ 98.35, 73.70, 75.57, 69.24, 65.57.

ESI-MS m/z: calcd. for $C_5H_{11}O_8P$ $[M-H]^-$:229.0, found: 228.8.

29: ^{1}H NMR (600 MHz, D_2O) δ 7.49 (dd, J = 7.3, 2.2 Hz, 2H), 7.38 – 7.30 (m, 3H), 4.66 (d, J = 9.7 Hz, 1H), 3.90 (dd, J = 11.4, 5.4 Hz, 1H), 3.53 (td, J = 9.9, 5.4 Hz, 1H), 3.40 (t, J = 9.0 Hz, 1H), 3.25 (q, J = 11.3, 10.3 Hz, 2H).

^{13}C NMR (151 MHz, D_2O) δ 132.08, 128.91, 88.06, 77.02, 71.67, 69.05, 68.76.

ESI-MS m/z: calcd. for $C_{11}H_{14}O_4S$ $[M+Cl]^-$:277.0, found: 276.8.

31: ^{1}H NMR (600 MHz, D_2O) δ 7.86 (d, J = 7.6 Hz, 2H), 7.75 (t, J = 7.5 Hz, 1H), 7.62 (t, J = 7.8 Hz, 2H), 4.57 (d, J = 9.5 Hz, 1H), 3.90 (dd, J = 11.4, 5.4 Hz, 1H), 3.60 (t, J = 9.2 Hz, 1H), 3.52 – 3.39 (m, 2H), 3.20 (t, J = 10.9 Hz, 1H).

^{13}C NMR (151 MHz, D_2O) δ 135.39, 129.56, 129.1, 91.29, 76.85, 69.31, 69.28, 68.31.

ESI-MS m/z: calcd. for $C_{11}H_{14}O_6S$ $[M+Na]^+$:297.0, found: 296.8.

33: ^{1}H NMR (600 MHz, D_2O) δ 7.63 – 7.57 (m, 2H), 7.39 – 7.29 (m, 3H), 4.86 (dd, J = 9.6, 0.8 Hz, 1H), 3.92 (dd, J = 11.4, 5.3 Hz, 1H), 3.58 – 3.43 (m, 1H), 3.37 (t, J = 8.9 Hz, 1H), 3.29 (ddd, J = 9.4, 8.7, 0.7 Hz, 1H), 3.25 – 3.18 (m, 1H).

^{13}C NMR (151 MHz, D_2O) δ 134.52, 129.4, 128.65, 126.54, 84.62, 77.02, 72.25, 69.86, 68.93.

ESI-MS m/z: calcd. $C_{11}H_{14}O_4Se$ $[M+Na]^+$:313.0, found: 313.0.

35: ^{1}H NMR (500 MHz, D_2O) δ 7.76 (d, J = 16.0 Hz, 1H), 7.56 – 7.49 (m, 2H), 6.91 – 6.84 (m, 2H), 6.38 (d, J = 15.9 Hz, 1H), 5.54 – 5.47 (m, 1H), 3.95 (dd, J = 11.5, 5.4 Hz, 1H), 3.66 – 3.59 (m, 1H), 3.55 – 3.46 (m, 2H), 3.4 (dd, J = 11.7, 10.3 Hz, 1H).

^{13}C NMR (126 MHz, D_2O) δ 147.95, 130.76, 115.84, 113.05, 94.71, 75.39, 71.85, 69.01, 65.83.

ESI-MS m/z: calcd. for $C_{14}H_{16}O_7$ $[M-H]^-$:295.1, found: 294.8.

36: ^{1}H NMR (500 MHz, D_2O) δ 7.60 – 7.53 (m, 3H), 7.08 (d, J = 8.2 Hz, 2H), 6.41 (d, J = 16.3 Hz, 1H), 5.08 (d, J = 5.3 Hz, 1H), 3.97 (dd, J = 11.9, 5.2 Hz, 1H), 3.66 (d, J = 8.7 Hz, 1H), 3.53 – 3.39 (m, 3H).

^{13}C NMR (126 MHz, D_2O) δ 144.25, 129.98, 117.65, 116.75, 100.27, 75.4, 72.74, 68.99, 65.18.

ESI-MS m/z: calcd. for $C_{14}H_{16}O_7$ $[M-H]^-$:295.1, found: 294.8.

37: ^{1}H NMR (500 MHz, D_2O) δ 7.77 (d, J = 16.0 Hz, 1H), 7.64 – 7.57 (m, 2H), 7.13 – 7.02 (m, 2H), 6.44 (d, J = 16.0 Hz, 1H), 5.57 – 5.49 (m, 1H), 5.13 – 5.04 (m, 1H), 3.96 (ddd, J = 11.6, 5.4, 1.9 Hz, 2H), 3.64 (dddd, J = 10.3, 8.9, 7.0, 5.1 Hz, 2H), 3.55 – 3.36 (m, 6H).

^{13}C NMR (126 MHz, D_2O) δ 147.39, 130.47, 116.75, 114.73, 100.17, 94.81, 75.47, 72.7, 71.85, 69.03, 65.82, 65.27.

ESI-MS m/z: calcd. for $C_{19}H_{24}O_{11}$ $[M+Na]^+$:451.1, found: 451.2.

39: ^{1}H NMR (600 MHz, D_2O) δ 7.66 (d, J = 16.0 Hz, 1H), 7.15 (d, J = 2.0 Hz, 1H), 7.07 (dd, J = 8.2, 1.9 Hz, 1H), 6.84 (d, J = 8.2 Hz, 1H), 6.35 (d, J = 15.9 Hz, 1H), 5.54 – 5.47 (m, 1H), 3.95 (dd, J = 11.6, 5.5 Hz, 1H), 3.80 (s, 3H), 3.67 – 3.59 (m, 1H), 3.54 – 3.46 (m, 2H), 3.41 (dd, J = 11.7, 10.3 Hz, 1H).

^{13}C NMR (151 MHz, D_2O) δ 167.71, 147.97, 147.6, 126.69, 123.76, 115.53, 113.18, 111.36, 94.77, 75.35, 71.9, 69.01, 65.8, 55.82.

ESI-MS m/z: calcd. for $C_{15}H_{18}O_8$ $[M-H]^-$:325.1, found: 324.9.

40: ^{1}H NMR (600 MHz, D_2O) δ 7.55 (d, J = 15.9 Hz, 1H), 7.21 (d, J = 2.0 Hz, 1H), 7.13 (dd, J = 8.4, 1.9 Hz, 1H), 7.07 (d, J = 8.4 Hz, 1H), 6.35 (d, J = 16.1 Hz, 1H), 5.04 (d, J = 7.7 Hz, 1H), 3.95 (dd, J = 11.6, 5.5 Hz, 1H), 3.82 (s, 3H), 3.69 – 3.62 (m, 1H), 3.58 – 3.46 (m, 2H), 3.39 (t, J = 11.0 Hz, 1H).

^{13}C NMR (151 MHz, D_2O) δ 171.28, 148.97, 147.19, 145.57, 129.76, 122.57, 116.57, 115.9, 111.6, 100.74, 75.41, 72.71, 69.07, 65.35, 55.89.

ESI-MS m/z: calcd. for $C_{15}H_{18}O_8$ $[M-H]^-$:325.1, found: 324.9.

41: ^{1}H NMR (600 MHz, D_2O) δ 7.71 (d, J = 16.0 Hz, 1H), 7.23 (s, 1H), 7.16 (d, J = 8.4 Hz, 1H), 7.09 (d, J = 8.3 Hz, 1H), 6.43 (d, J = 15.9 Hz, 1H), 5.53 (d, J = 7.0 Hz, 1H), 5.05 (d, J = 7.6 Hz, 1H), 3.95 (td, J = 12.1, 5.4 Hz, 2H), 3.82 (s, 3H), 3.65 (qd, J = 9.1, 8.7, 5.1 Hz, 2H), 3.58 – 3.46 (m, 4H), 3.4 (q, J = 11.8 Hz, 2H).

^{13}C NMR (151 MHz, D_2O) δ 167.42, 148.97, 147.58, 147.46, 129.38, 123.13, 115.84, 114.86, 111.72, 100.65, 94.81, 75.33, 72.65, 71.97, 69.03, 68.96, 65.55, 65.35, 55.94.

ESI-MS m/z: calcd. for $C_{20}H_{26}O_{12}$ $[M+Cl]^-$:493.1, found: 492.9.

SUPPLEMENTARY REFERENCES

1 Nieto-Domínguez, M. *et al.* Novel pH-stable glycoside hydrolase family 3 β-xylosidase from *Talaromyces amestolkiae*: An enzyme displaying regioselective transxylosylation. *Appl. Environ. Microbiol.* **81**, 6380-6392, (2015).

2 Han, Y. W. & Srinivasan, V. R. Purification and characterization of β-glucosidase of *Alcaligenes faecalis*. *J. Bacteriol.* **100**, 1355-1363, (1969).

3 Jahn, M., Marles, J., Warren, R. A. J. & Withers, S. G. Thioglycoligases: Mutant glycosidases for thioglycoside synthesis. *Angew. Chem. Int. Ed. Engl.* **42**, 352-354, (2003).

4 Zechel, D. L. *et al.* Mechanism, mutagenesis, and chemical rescue of a β-mannosidase from *Cellulomonas fimi*. *Biochemistry* **42**, 7195-7204, (2003).

5 Stoll, D., Stålbrand, H. & Warren, R. A. Mannan-degrading enzymes from *Cellulomonas fimi*. *Appl. Environ. Microbiol.* **65**, 2598-2605, (1999).

6 Salleh, H. M. *et al.* Cloning and characterization of *Thermotoga maritima* β-glucuronidase. *Carbohydr. Res.* **341**, 49-59, (2006).

7 Müllegger, J. *et al.* Thermostable glycosynthases and thioglycoligases derived from *Thermotoga maritima* β-glucuronidase. *ChemBioChem* **7**, 1028-1030, (2006).

8 McIntosh, L. P. *et al.* The p*K*a of the general acid/base carboxyl group of a glycosidase cycles during catalysis: A 13C-NMR study of *Bacillus circulans* xylanase. *Biochemistry* **35**, 9958-9966, (1996).

9 Armstrong, Z., Reitinger, S., Kantner, T. & Withers, S. G. Enzymatic thioxyloside synthesis: Characterization of thioglycoligase variants identified from a site-saturation mutagenesis library of *Bacillus circulans* xylanase. *ChemBioChem* **11**, 533-538, (2010).

10 Kim, Y. H. *et al.* Effect on product specificity of cyclodextrin glycosyltransferase by site-directed mutagenesis. *IUBMB Life* **41**, 227-234, (2008).

11 Shim, J.-H. *et al.* Improvement of cyclodextrin glucanotransferase as an antistaling enzyme by error–prone PCR. *Protein Eng. Des. Sel.* **17**, 205-211, (2004).

12 Li, C., Ahn, H. J., Kim, J. H. & Kim, Y. W. Transglycosylation of engineered cyclodextrin glucanotransferases as *O*-glycoligases. *Carbohydr. Polym.* **99**, 39-46, (2014).

13 Mark, B. L. *et al.* Structural and functional characterization of *Streptomyces plicatus* β-*N*-acetylhexosaminidase by comparative molecular modeling and site-directed mutagenesis. **273**, 19618-19624, (1998).

14 Tegl, G. *et al.* Facile formation of β-thioGlcNAc linkages to thiol-containing sugars, peptides, and proteins using a mutant GH20 hexosaminidase. *Angew. Chem. Int. Ed. Engl.* **58**, 1632-1637, (2019).

15 Rolfsmeier, M. & Blum, P. Purification and characterization of a maltase from the extremely thermophilic crenarchaeote *Sulfolobus solfataricus*. *J. Bacteriol.* **177**, 482-485, (1995).

16 Kim, Y. W. *et al.* Expanding the thioglycoligase strategy to the synthesis of α-linked thioglycosides allows structural investigation of the parent enzyme/substrate complex. *J. Am. Chem. Soc.* **128**, 2202-2203, (2006).

17 Okuyama, M., Mori, H., Chiba, S. & Kimura, A. Overexpression and characterization of two unknown proteins, YicI and YihQ, originated from *Escherichia coli*. *Protein Expr. Purif.* **37**, 170-179, (2004).

18 Wong-Madden, S. T. & Landry, D. Purification and characterization of novel glycosidases from the bacterial genus *Xanthomonas*. *Glycobiology* **5**, 19-28, (1995).

19 Kim, Y. W., Chen, H., Kim, J. H. & Withers, S. G. Catalytic properties of a mutant β-galactosidase from *Xanthomonas manihotis* engineered to synthesize galactosyl-thio-β-1,3 and -β-1,4-glycosides. *FEBS Lett.* **580**, 4377-4381, (2006).

20 Chlubnová, I. *et al.* Enzymatic synthesis of oligo-d-galactofuranosides and l-arabinofuranosides: from molecular dynamics to immunological assays. *Org. Biomol. Chem.* **8**, 2092-2102, (2010).

21 Almendros, M. *et al.* Exploring the synthetic potency of the first furanothioglycoligase through original remote activation. *Org. Biomol. Chem.* **9**, 8371-8378, (2011).

22 Ficko-Blean, E., Stubbs, K. A., Nemirovsky, O., Vocadlo, D. J. & Boraston, A. B. Structural and mechanistic insight into the basis of mucopolysaccharidosis IIIB. *Proc. Natl. Acad. Sci. USA* **105**, 6560-6565, (2008).

23 Tshililo, N. O. *et al.* The α-thioglycoligase derived from a GH89 α-*N*-acetylglucosaminidase synthesises α-*N*-acetylglucosamine-based glycosides of biomedical interest. *Adv. Synth. Catal.* **359**, 663-676, (2017).

24 Wang, Q., Trimbur, D., Graham, R., Warren, R. A. J. & Withers, S. G. Identification of the acid/base catalyst in *Agrobacterium faecalis* β-glucosidase by kinetic analysis of mutants. *Biochemistry* **34**, 14554-14562, (1995).

25 Nieto-Domínguez, M. *et al.* Enzymatic fine-tuning for 2-(6-hydroxynaphthyl) β-D-xylopyranoside synthesis catalyzed by the recombinant β-xylosidase BxTW1 from *Talaromyces amestolkiae*. *Microb. Cell Fact.* https://doi.org/10.1186/s12934-12016-10568-12936 (2016).
