# Supplementary material for: Thioglycoligase derived from fungal GH3 β-xylosidase is a multi-glycoligase with broad acceptor tolerance
Source: Nat Commun. 2020 Sep 25;11:4864. doi: 10.1038/s41467-020-18667-3 (PMC7519651; doi:10.1038/s41467-020-18667-3)
Supplement: Supplementary file 3 — Description of Additional Supplementary Files [file 41467_2020_18667_MOESM3_ESM.pdf]

**Descriptions of Additional Supplementary Files**

**Supplementary Data 1**

**Description:** List of assayed compounds and conditions in the acceptor screening of rBxTW1-E495A. Data include the pKa of the acceptors together with the source of the value(s). In addition, the concentration of the acceptor, the selected co-solvent and the eluent for the TLC analysis are reported.

**Supplementary Data 2**

**Description:** Summary of conditions for the monitoring and purification by HPLC of a series of glycosides obtained using rBxTW1-E495A. The file includes information on the column, the solvents, the running procedure and the suitable wavelength to detect the acceptor and the corresponding gl ycoconjugates.
