# Supplementary material for: Thioglycoligase derived from fungal GH3 β-xylosidase is a multi-glycoligase with broad acceptor tolerance
Source: Nat Commun. 2020 Sep 25;11:4864. doi: 10.1038/s41467-020-18667-3 (PMC7519651; doi:10.1038/s41467-020-18667-3)
Supplement: Supplementary file 6 — Reporting Summary [file 41467_2020_18667_MOESM6_ESM.pdf]

Corresponding author(s): Manuel Nieto-Domínguez, María Jesús Martínez

Last updated by author(s): Aug 13, 2020

Reporting Summary

Nature Research wishes to improve the reproducibility of the work that we publish. This form provides structure for consistency and transparency in reporting. For further information on Nature Research policies, see our Editorial Policies and the Editorial Policy Checklist.

Statistics

For all statistical analyses, confirm that the following items are present in the figure legend, table legend, main text, or Methods section.

n/a	Confirmed	
☐	☒	The exact sample size (*n*) for each experimental group/condition, given as a discrete number and unit of measurement
☐	☒	A statement on whether measurements were taken from distinct samples or whether the same sample was measured repeatedly
☐	☒	The statistical test(s) used AND whether they are one- or two-sided<br>*Only common tests should be described solely by name; describe more complex techniques in the Methods section.*
☒	☐	A description of all covariates tested
☒	☐	A description of any assumptions or corrections, such as tests of normality and adjustment for multiple comparisons
☐	☒	A full description of the statistical parameters including central tendency (e.g. means) or other basic estimates (e.g. regression coefficient) AND variation (e.g. standard deviation) or associated estimates of uncertainty (e.g. confidence intervals)
☐	☒	For null hypothesis testing, the test statistic (e.g. *F*, *t*, *r*) with confidence intervals, effect sizes, degrees of freedom and *P* value noted<br>*Give P values as exact values whenever suitable.*
☒	☐	For Bayesian analysis, information on the choice of priors and Markov chain Monte Carlo settings
☒	☐	For hierarchical and complex designs, identification of the appropriate level for tests and full reporting of outcomes
☒	☐	Estimates of effect sizes (e.g. Cohen's *d*, Pearson's *r*), indicating how they were calculated

Our web collection on statistics for biologists contains articles on many of the points above.

Software and code

Policy information about availability of computer code

Data collection	Bruker's TopSpin 2.1, DataAnalysis 3.4 (Bruker Daltonics), Masshunter Data Acquisition B.05.01 (Agilent Technologies), Masshunter Qualitative Analysis B.07.00 (Agilent Technologies).
Data analysis	Design-Expert® 10.0.1.0 (Stat-Ease Inc.), SigmaPlot® 14.0 (Systat Software Inc.), SWISS-MODEL (available from https://swissmodel.expasy.org/), PrimerX (avialable from http://www.bioinformatics.org/primerx/), PyMOL Molecular Graphics System version 1.3.

For manuscripts utilizing custom algorithms or software that are central to the research but not yet described in published literature, software must be made available to editors and reviewers. We strongly encourage code deposition in a community repository (e.g. GitHub). See the Nature Research guidelines for submitting code & software for further information.

Data

Policy information about availability of data

All manuscripts must include a data availability statement. This statement should provide the following information, where applicable:
- Accession codes, unique identifiers, or web links for publicly available datasets
- A list of figures that have associated raw data
- A description of any restrictions on data availability

Data supporting the findings of this work are available within the paper and its Supplementary Information files and from the corresponding author upon reasonable request. A reporting summary for this Article is available as a Supplementary Information file. The source data underlying Figs. 3, 4, 5, 6, as well as Supplementary Table 2 and Supplementary Figures 1, 2, 6 and 8 are available as a Source Data file. Protein crystal structures were obtained from Protein Data Bank (PDB: 3U4A; PDB: 2X40; PDB :4I3G; PDB: 3ZYZ; PDB: 3AC0; PDB: 5A7M). The nucleotide sequence of bxtw1 is publicly available at Genbank under the accession no. KP119719.

Field-specific reporting

Please select the one below that is the best fit for your research. If you are not sure, read the appropriate sections before making your selection.

☒ Life sciences ☐ Behavioural & social sciences ☐ Ecological, evolutionary & environmental sciences

For a reference copy of the document with all sections, see nature.com/documents/nr-reporting-summary-flat.pdf

Life sciences study design

All studies must disclose on these points even when the disclosure is negative.

Sample size	The study was performed with purified enzymes, so a sample size as related to population studies is not applicable to this study.
Data exclusions	A few points corresponding to the highest values of substrate concentration were remove form the kinetics calculations displayed in Supplementary Table 2. Those values are indicated in the Source Data file and were excluded because showing clear substrate inhibition. No other data were excluded.
Replication	Only 1 replicate was performed when the experiment only aimed to screen the presence or absence of the expected product (screening reactions and ESI-MS experiments). The rest of the experiments were done in duplicate or triplicate. Reported measurements were taken from individual samples as indicated in the methods section (independent experiments). The data presented represent averages of independent experiments; all experimental data are included in these averages (no data exclusions). All attempts at replication were successful.<br><br>The experiments of glycosylation of phosphoric acid in Fig. 5 are the exception. These experiments were done only once by continuously measuring the corresponding sample over time, therefore there are no standard deviations. Due to the disadvantages of this acceptor commented in the manuscript (unknown application and high concentration and long reaction time required) the goal of the experiment was simply to verify that the product was formed and subsequently consumed in the assay conditions. Due to the testing of multiple timepoints and two different conditions, the results clearly answer the former scientific question.
Randomization	This is study was performed with purified enzymes, so randomization in independent groups was not applicable to this study.
Blinding	This study was performed with purified enzymes, therefore blinding tests were not performed as it was no relevant to this study.

Reporting for specific materials, systems and methods

We require information from authors about some types of materials, experimental systems and methods used in many studies. Here, indicate whether each material, system or method listed is relevant to your study. If you are not sure if a list item applies to your research, read the appropriate section before selecting a response.

Materials & experimental systems

n/a	Involved in the study
☒	☐ Antibodies
☐	☒ Eukaryotic cell lines
☒	☐ Palaeontology and archaeology
☒	☐ Animals and other organisms
☒	☐ Human research participants
☒	☐ Clinical data
☒	☐ Dual use research of concern

Methods

n/a	Involved in the study
☒	☐ ChIP-seq
☒	☐ Flow cytometry
☒	☐ MRI-based neuroimaging

Eukaryotic cell lines

Policy information about cell lines

Cell line source(s)	Pichia pastoris GS115 strain (Invitrogen). It is derived from the wild-type strain NRRL-Y 11430 (Northern Regional Research Laboratories). It has a mutation in the histinol dehydrogenase gene (HIS4) and was generated by nitrosoguanidine mutagenesis at Phillips Petroleum Co. It is the most frequently used Pichia strain for heterologous protein production
Authentication	*Describe the authentication procedures for each cell line used OR declare that none of the cell lines used were authenticated.*
Mycoplasma contamination	*Confirm that all cell lines tested negative for mycoplasma contamination OR describe the results of the testing for mycoplasma contamination OR declare that the cell lines were not tested for mycoplasma contamination.*
Commonly misidentified lines (See ICLAC register)	*Name any commonly misidentified cell lines used in the study and provide a rationale for their use.*
